# Supplementary material for: Phylogenomic profiles of whole-genome duplications in Poaceae and landscape of differential duplicate retention and losses among major Poaceae lineages
Source: Nat Commun. 2024 Apr 17;15:3305. doi: 10.1038/s41467-024-47428-9 (PMC11024178; doi:10.1038/s41467-024-47428-9)
Supplement: Supplementary file 1 — Supplementary Information [file 41467_2024_47428_MOESM1_ESM.pdf]

Phylogenomic Profiles of Whole-Genome Duplications in Poaceae and Landscape of Differential Duplicate Retention and Losses among Major Poaceae Lineages

Taikui Zhang, Weichen Huang, Lin Zhang, De-Zhu Li, Ji Qi, Hong Ma

Supplementary Information file

This pdf file contains:

Supplementary Figures 1-57

Supplementary References

Supplementary Figures

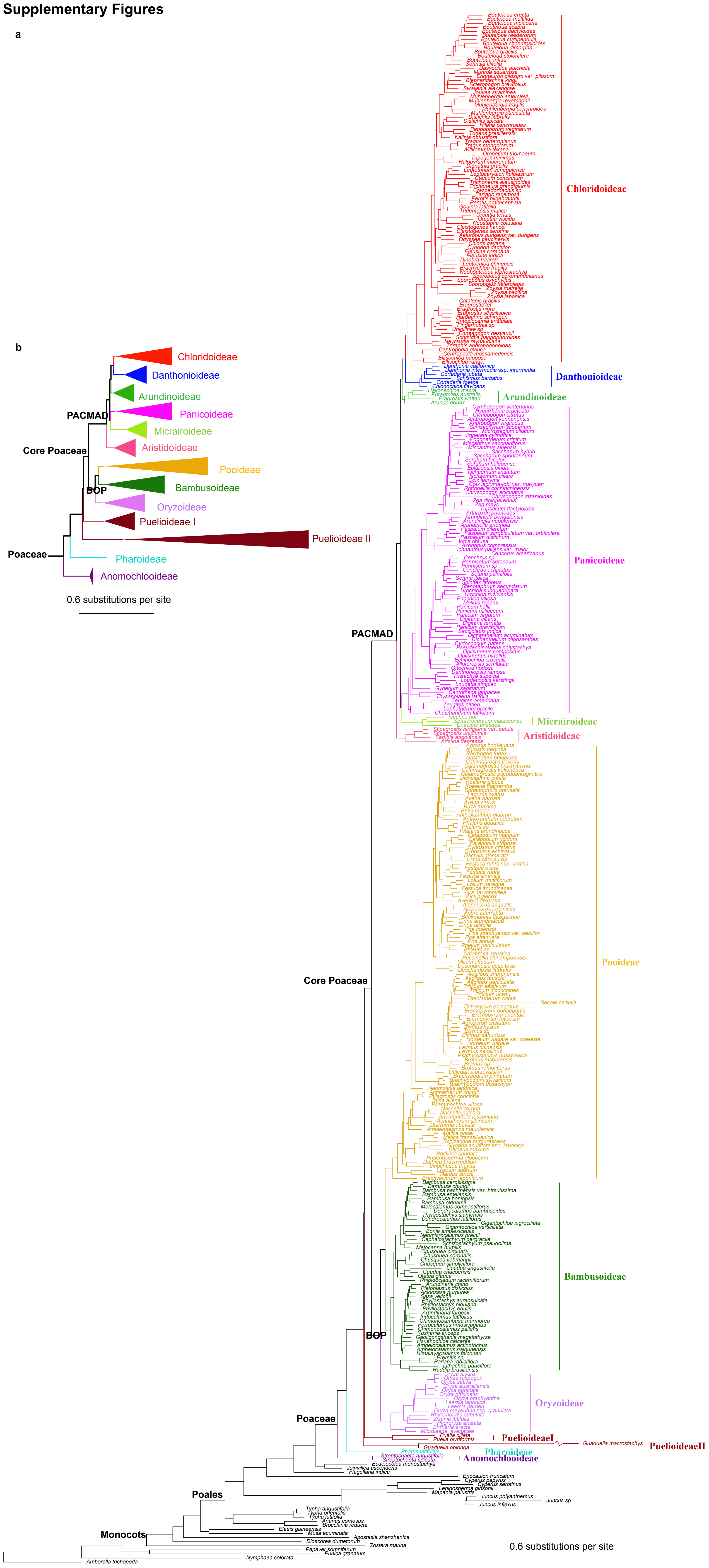

Supplementary Figure 1 Summary of phylogenetic relationships among Poaceae species
(**a**) An ML phylogenetic tree with color branches depicting relationships among grasses. Source data are provided as a Source Data file. (**b**) Cladogram simplified from the part a illustrating grass subfamilial relationships. The color code of branching is as part a. Bars in (a) and (b) indicate the average substitutions per site as shown in each part.

a

Summary of fossils for dating

Fossil	MRCA	Time (Mys)	Type	References
F1	CG Angiosperms	209 (>1.87<2.67)*	Pollen grains	1
F2	CG Monocots	113	A whole monocotyledonous plant	2
F3	CG Poaceae	101	Silicified epidermal pieces and phytoliths	3
F4	CG Core Poaceae	55	Inflorescence, spikelet and pollen	4
F5	CG Oryzoideae	66	Epidermis and phytolith	5
F6	CG Pooideae	40	Phytolith	6
F7	CG Chloridoideae	19	Phytolith	7
F8	SG Chusquea	35	Phytolith	7
F9	SG Leersia	30.44	Inflorescence	8
F10	SG Stipeae	34	Fruits	9
F11	SG Zingiberales	77	Seeds	5
F12	CG Eudicots	125	Tricolpate pollen grains	10

* The dated divergence time (209 Mys) is used as a sencondary constraint to fix the crown group of angiosperms in baseml.

b

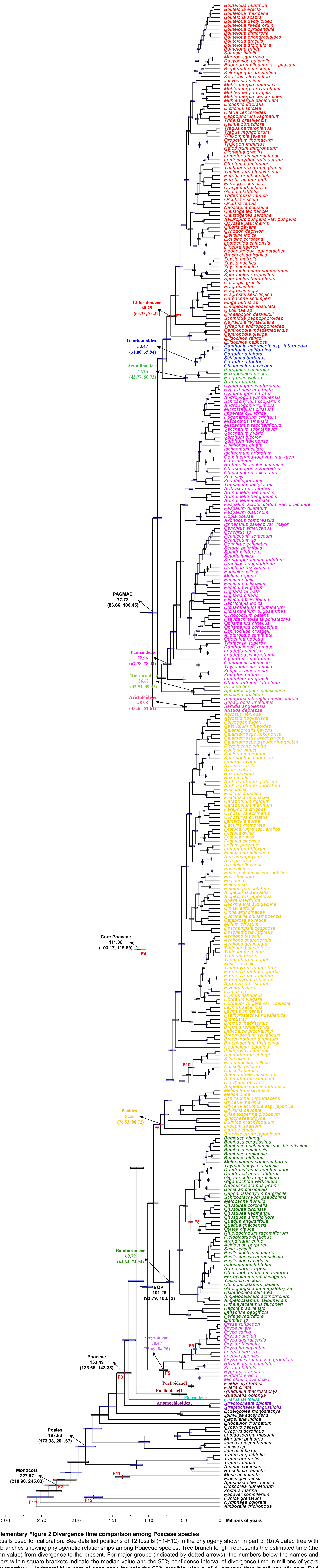

Supplementary Figure 2 Divergence time comparison among Poaceae species
(**a**) Fossils used for calibration. See detailed positions of 12 fossils (F1-F12) in the phylogeny shown in part b. (**b**) A dated tree with color branches showing phylogenetic relationships among Poaceae species. Tree branch length represents the estimated time (the median value) from divergence to the present. For major groups (indicated by dotted arrows), the numbers below the names and numbers within square brackets indicate the median value and the 95% confidence interval of divergence time in millions of years ago, respectively. Horizontal blue bars at each node indicate the 95% credible interval of divergence time in millions of years. Red points (F1-F12) at nodes indicate the constrained nodes with fossils or the previously reported divergence time as summarized in part a. Source data are provided as a Source Data file.

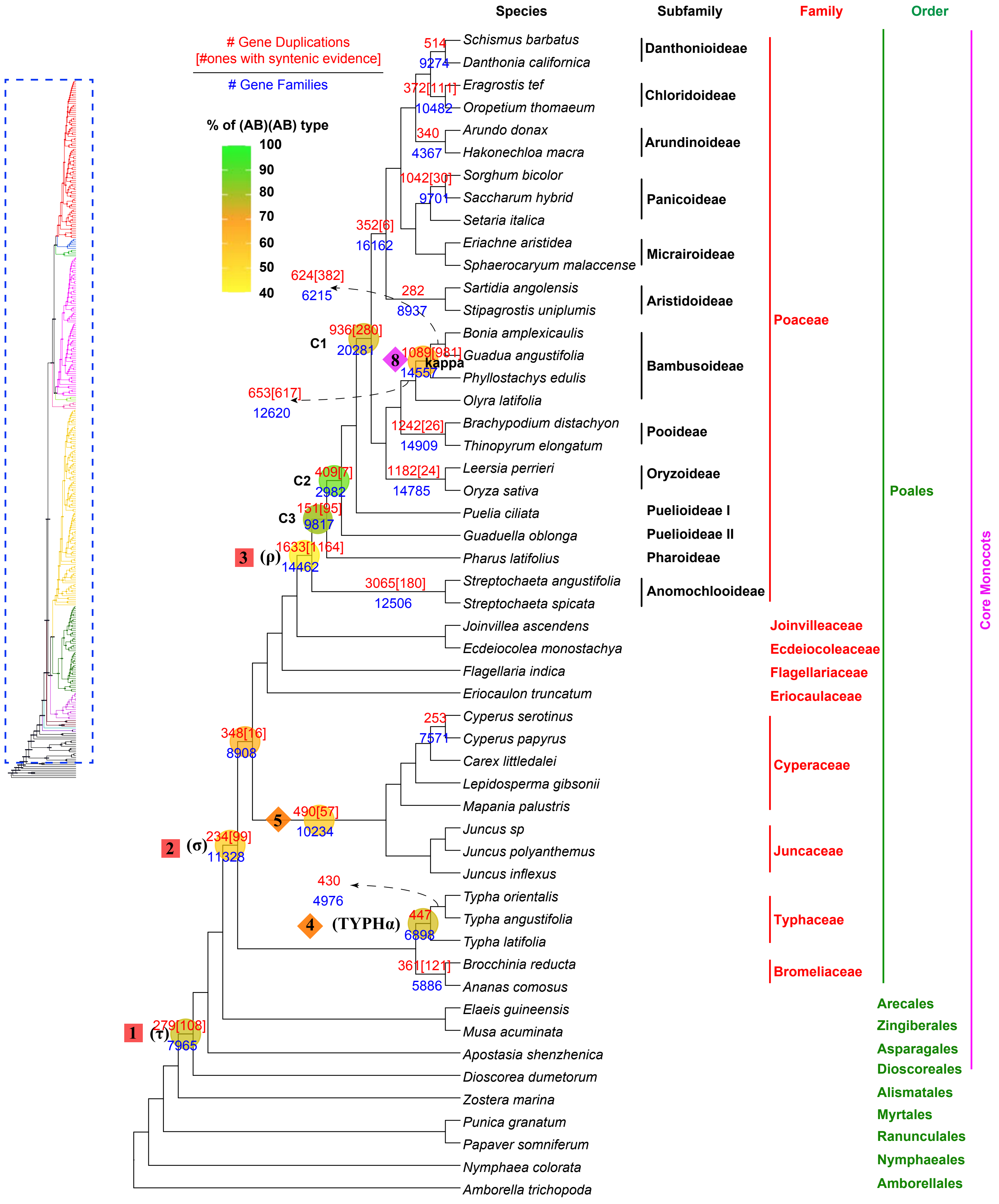

Supplementary Figure 3 WGDs and GD bursts in Poales detected by phylogenomic analyses via Tree2GD

(left) Cladogram simplified from Supplementary Fig. 2, with branching in the dotted box depicting relationships in Poales; (right) cladogram, an expanded view of the left box, with red numbers above branches and blue numbers below the branches representing the number of GDs and GFs, respectively. Numbers in square brackets represent the numbers of GDs matched by syntenic genes from at least one genome sequenced species. Circle above branch indicates the percentage of GDs in the (AB)(AB) retention type (with two duplicates retained in both of the sister lineages A and B); percentages are indicated in the color scale shown at top-left. Six WGDs are highlighted by the same WGD numbers and symbols in Figure 1 and are supported by comparison of GDs numbers, number of GDs with (AB)(AB) type, and those matching syntenic genes. These WGDs include tau shared by core monocots (#1), sigma shared by Poales (#2), rho shared by Poaceae (#3), the *Typha* WGD (#4), WGD shared by Cyperaceae and Juncaceae (#5), and kappa shared by woody bamboos (#8). In addition, there is a cluster of 3,065 GDs that are mapped at the MRCA of *Streptochaeta angustifolia* and *S. spicate* and include 180 GDs matching syntenic genes in *S. angustifolia*, suggesting a large-scale gene duplication event in *Streptochaeta*. Three other GD clusters (C1-C3) are mapped in Poaceae and discussed in Fig. 2. Source data are provided as a Source Data file.

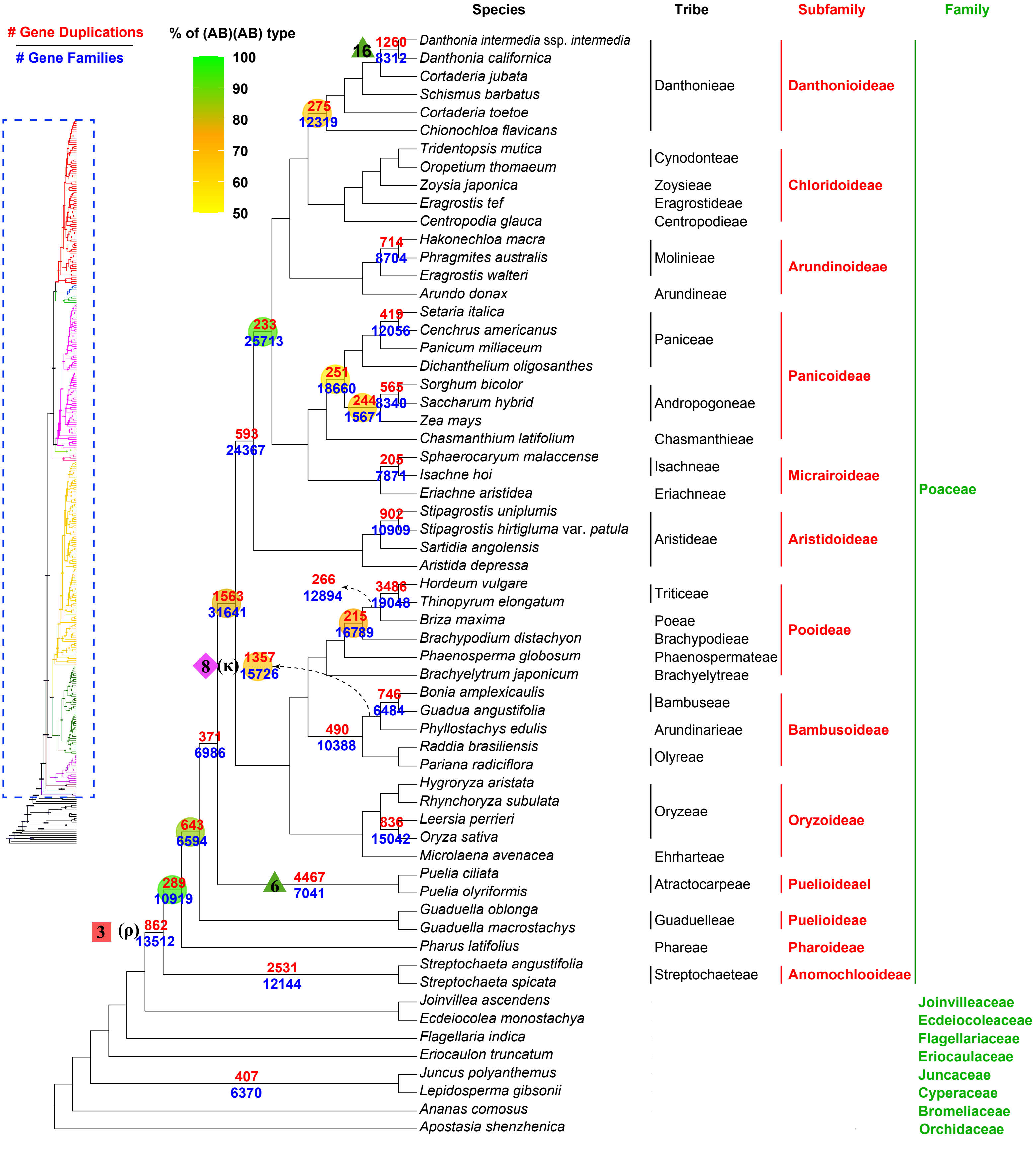

Supplementary Figure 4 WGDs and GD bursts in Poaceae detected by phylogenomic analyses via Tree2GD
Meanings of numbers and symbols in the phylogeny are same as those in Supplementary Fig. 3. In addition to the rho event (#3), three WGDs are proposed here, including the *Puelia* WGD (#6), kappa (#8), and the *Danthonia* WGD (#16). Source data are provided as a Source Data file.

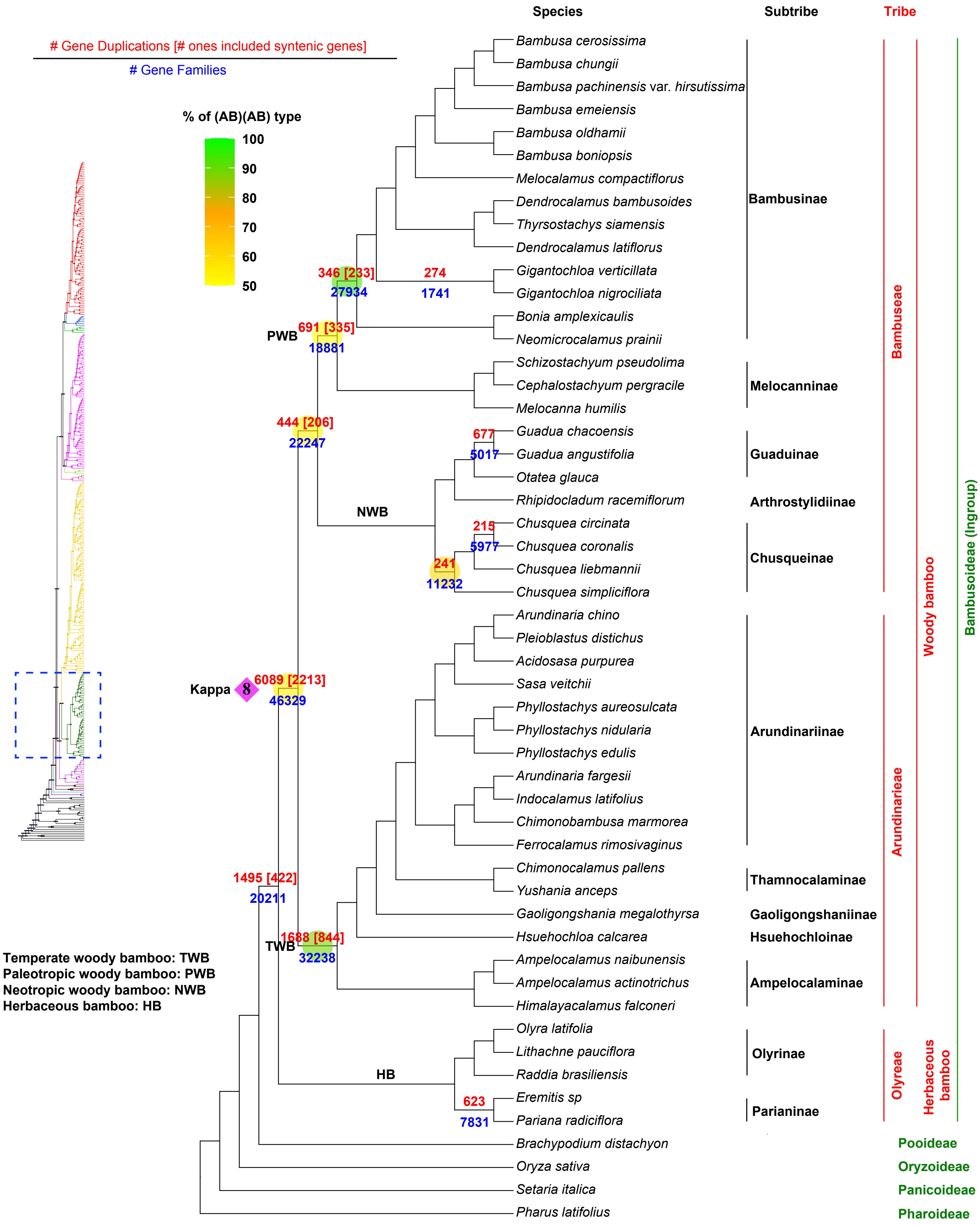

Supplementary Figure 5 WGD and GD bursts in Bambusoideae detected by phylogenomic analyses via Tree2GD
Meanings of numbers and symbols in the phylogeny are same as those in Supplementary Fig. 3. Woody bamboo WGD (#8; Kappa) is shared by the Bambuseae and Arundinarieae tribes. Source data are provided as a Source Data file.

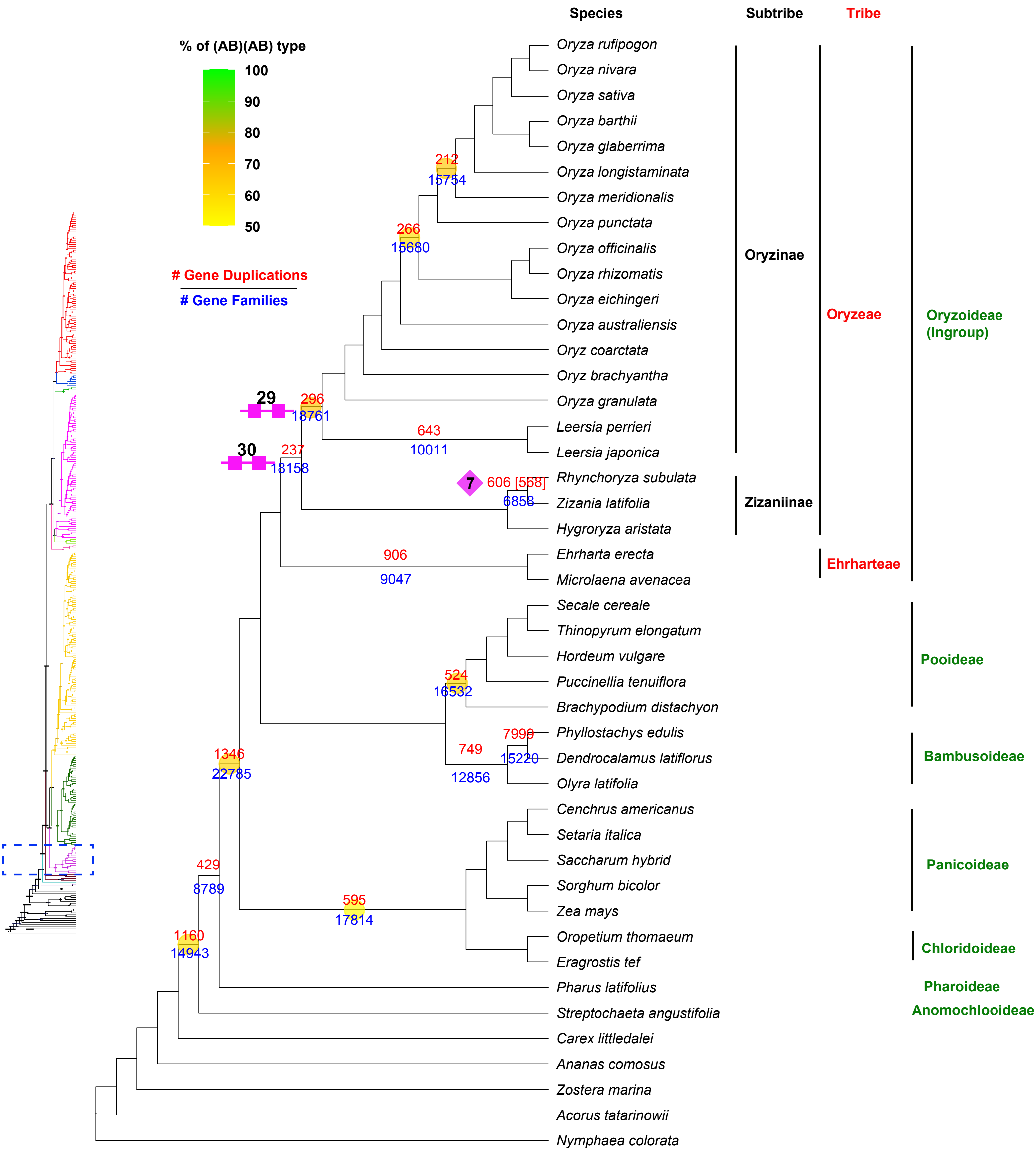

Supplementary Figure 6 WGD and GD bursts in Oryzoideae detected by phylogenomic analyses via Tree2GD

The Oryzoideae phylogeny is referred to the cladogram in Supplementary Fig. 41. Meanings of numbers and symbols in the phylogeny are same as those in Supplementary Fig. 3. Meanings of the circles, numbers, and the WGD signal in the phylogeny are same as those in Supplementary Fig. 3. The WGD (#7) in *Zizania latifolia* genome is also shared by *Rhynchoryza subulata* (See expanded discussion in Supplementary Fig. 20a). In addition, two clusters of tandem duplications (#29 and 30) are proposed in Oryzeae. Source data are provided as a Source Data file.

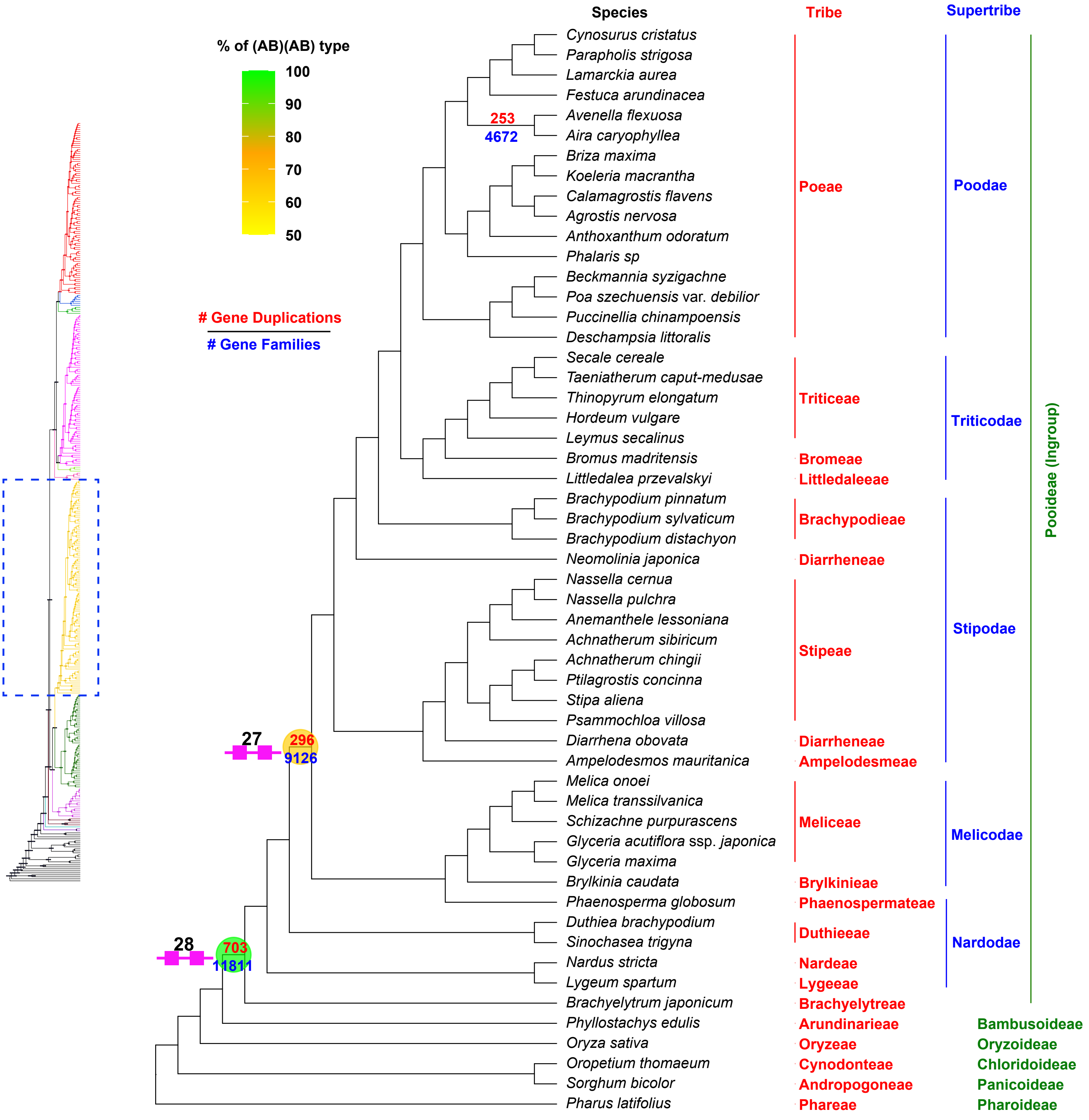

Supplementary Figure 7 GD bursts in Pooideae detected by phylogenomic analyses via Tree2GD

In the Pooideae phylogeny supertribes are assigned according to the recent grass classification (Soreng et al.[11]). Meanings of numbers and symbols in the phylogeny are same as those in Supplementary Fig. 3. In particular, paralogs from *Brachypodium distachyon* genome contribute to 167 of the 296 GDs mapped at the MRCA of Poeae and Meliceae tribes (#27) and to 191 of the 703 GDs mapped at Pooideae (#28). The tandem duplicates in *B. distachyon* match 20 of the 167 GDs and 14 of the 191 GDs. We proposed two possible tandem duplication clusters (#27 and 28) with evolutionary implications in Pooideae. Source data are provided as a Source Data file.

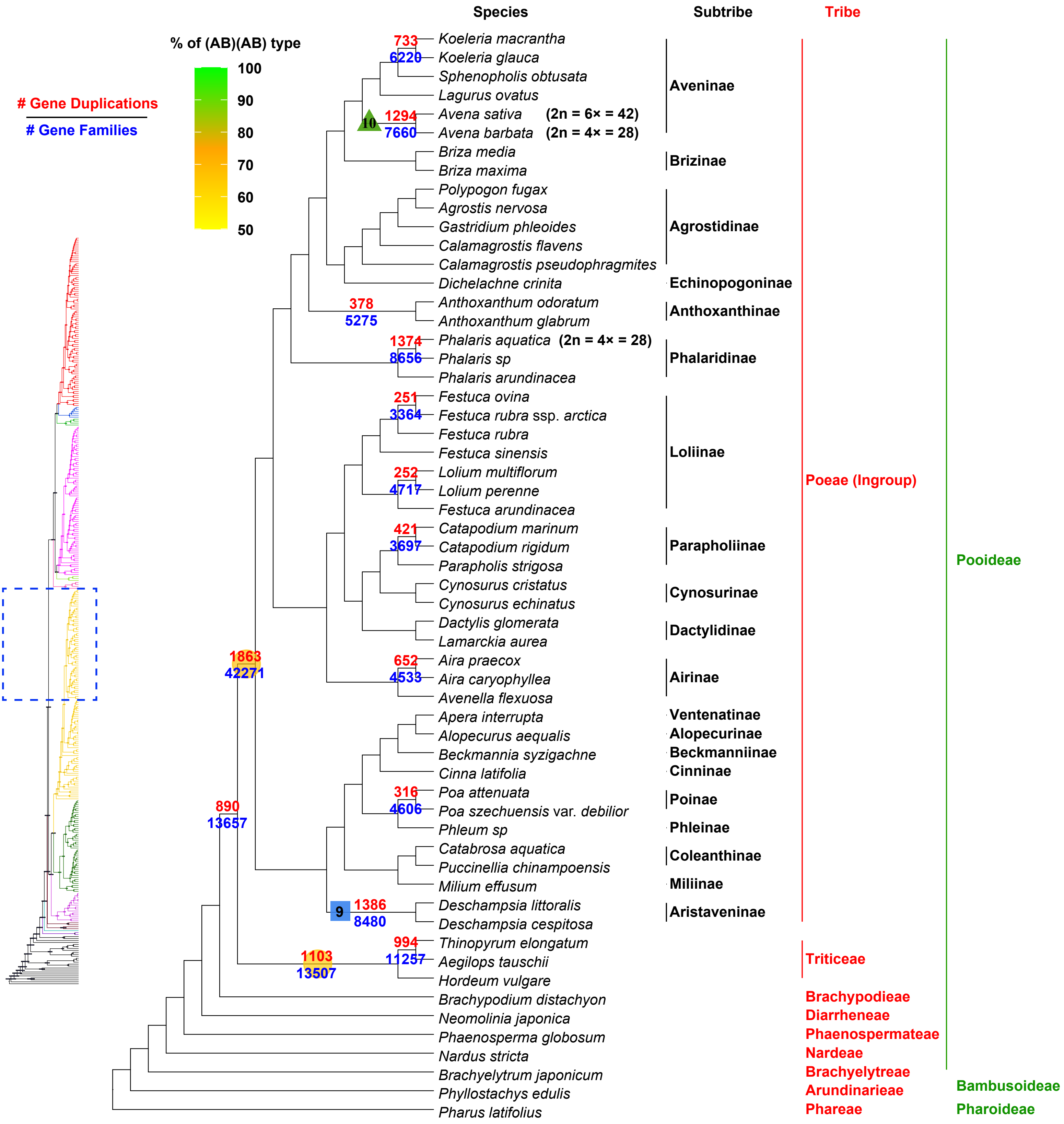

Supplementary Figure 8 WGDs and GD bursts in tribe Poeae of Pooideae detected by phylogenomic analyses via Tree2GD Meanings of numbers and symbols in the phylogeny are same as those in Supplementary Fig. 3. Two GD clusters are proposed as WGD events, including the *Avena* WGD (#10) and the *Deschampsia* WGD (#9). In addition, 1,863 GDs are mapped at the MRCA of Poeae and imply that ancient gene duplications possibly contributed to the diversification of this tribe. Another cluster of 1,374 GDs shared by *Phalaris aquatica* (2n=4x=28; A) and its close relative (B) include 633 GDs of the (AB)A type implicate a putative progenitor for the allotetraploidization in *P. aquatica* that can be further estimated by sequencing genomes of these species. Source data are provided as a Source Data file.

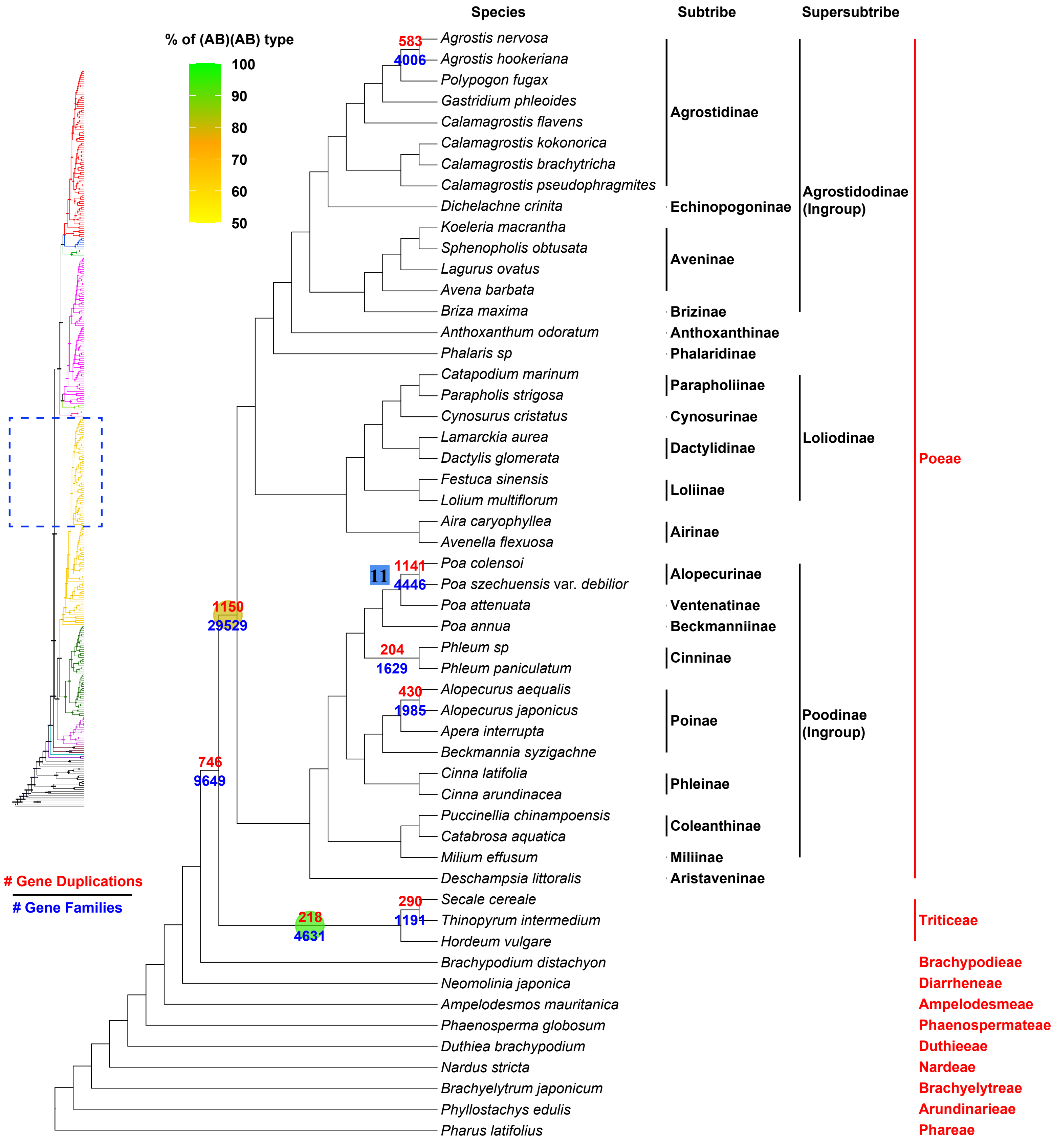

Supplementary Figure 9 WGDs and GD bursts in supersubtribes Agrostidodinae and Poodinae of the Poeae tribe detected by phylogenomic analyses via Tree2GD

Meanings of numbers and symbols in the phylogeny are same as those in Supplementary Fig. 3. A cluster of 1,141 GDs mapped at the MRCA of *Poa colensoi* and *P. szechuensis var debilior* is proposed as a WGD event (#11), which is supported by the recent phylotranscriptomic result[12]. Source data are provided as a Source Data file.

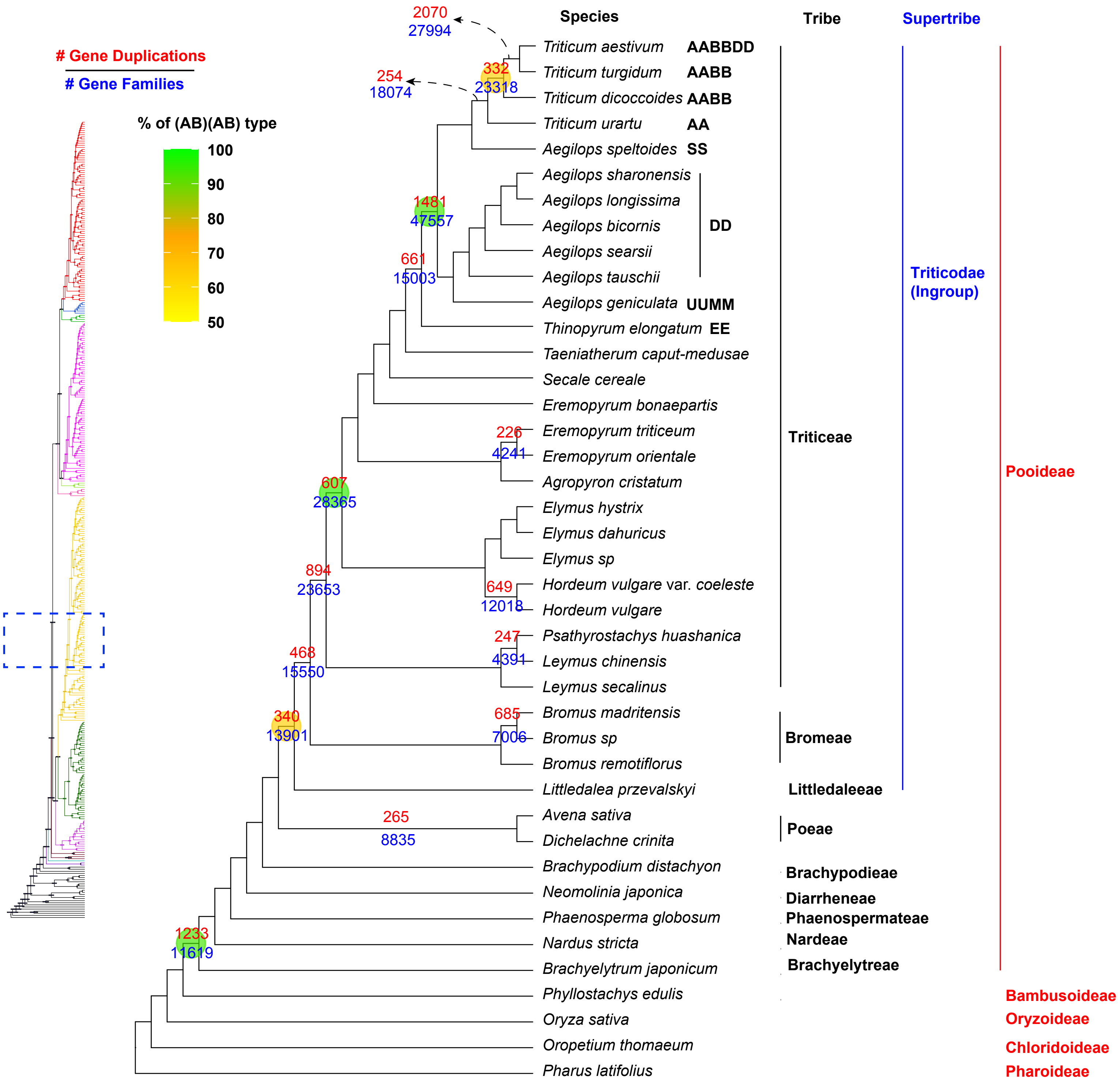

Supplementary Figure 10 GD bursts in suptribe Triticodae of Pooideae detected by phylogenomic analyses via Tree2GD Meanings of numbers and symbols in the phylogeny are same as those in Supplementary Fig. 3. A cluster of 1,481 GDs mapped at the MRCA of *Triticum* and *Aegilops* is consistent with the ancient hybridization in *Triticum/Aegilops*[12-14]. Source data are provided as a Source Data file.

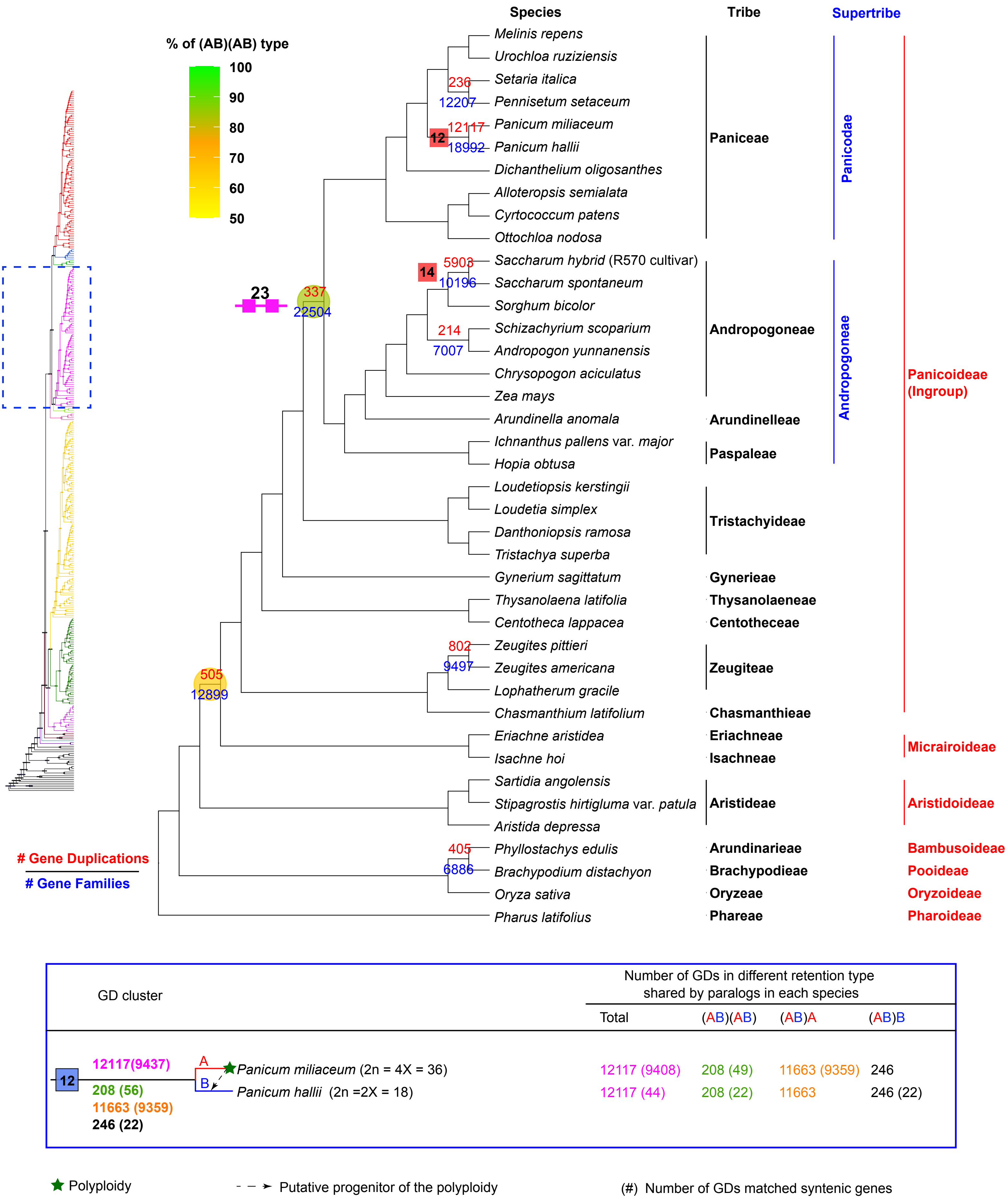

Supplementary Figure 11 WGDs and GD bursts in Panicoideae detected by phylogenomic analyses via Tree2GD
Meanings of numbers and symbols in the phylogeny are same as those in Supplementary Fig. 3. WGDs in this group include the event (#12) in *Panicum* and the event (#14) in *Saccharum*. See discussion of the GD cluster 23 in Supplementary Fig. 12. (below) Summary of the GDs in different retention type for #12. (AB)(AB) means a pattern of retention of two copies from the A and B lineages. (AB)A means a type of the retention of two copies from the A lineage and retention of only one copy from the B lineage. (AB)B means a type of the retention of two copies from the B lineage and retention of only one copy from the A lineage. Right numbers show the number of GDs contributed by the paralogs from each species in different retention types. The number in round parentheses represents the number of GDs matched by syntenic genes. Numbers of GDs in the (AB)(AB), (AB)A, (AB)B, and sum of the above 3 types are showed in green, orange, black, and purple colors, respectively. The number above the root branch represents the total number of GDs. Numbers below the branch represent the number of GDs in different retention types. About 96.3% of GDs mapped at the MRCA of *P. miliaceum* and *P. hallii* are retained in (AB)A type, indicating introgressions between the two *Panicum* species. It is probable that *P. hallii* might be one possible progenitor leading to the tetraploid *P. miliaceum*. Source data are provided as a Source Data file.

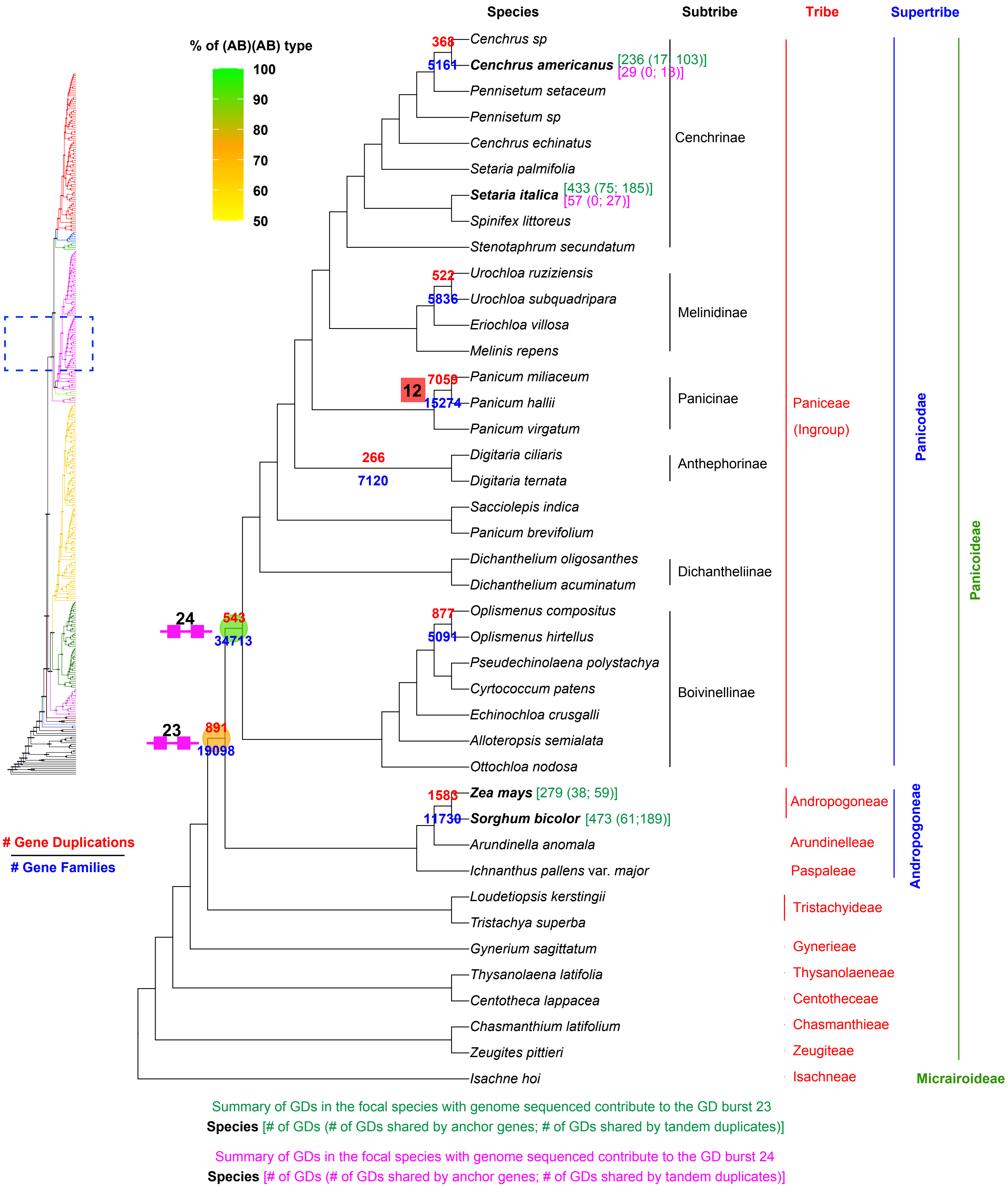

Supplementary Figure 12 WGD and GD bursts in supertribe Panicodae of Panicoideae detected by phylogenomic analyses via Tree2GD

Meanings of numbers and symbols in the phylogeny are same as those in Supplementary Fig. 3. The *Panicum* WGD (#12) is identified as a known WGD event. Paralogs from *Cenchrus americanus* and *Setaria italica* contribute to 29 and 57 GDs of a cluster of 543 GDs mapped at Panicodae, respectively. 13 of the 29 GDs and 27 of the 57 GDs are anchored in tandem duplicated regions, implying an ancient SSD event (#24) contributing to the GD cluster at Panicodae. Similar to the Panicodae GD cluster, paralogs from *C. americanus*, *S. italica*, *Sorghum bicolor*, and *Zea mays* contribute to 236, 433, 473 and 279 GDs of a GD cluster mapped at the MRCA of Andropogonodae and Panicodae, respectively. 103 of the 236 GDs, 185 of the 433 GDs, 189 of the 473 GDs and 59 of the 279 GDs are detected as tandem duplicates, showing an ancient SSD event (#23) prior to the divergence of Andropogonodae and Panicodae. Source data are provided as a Source Data file.

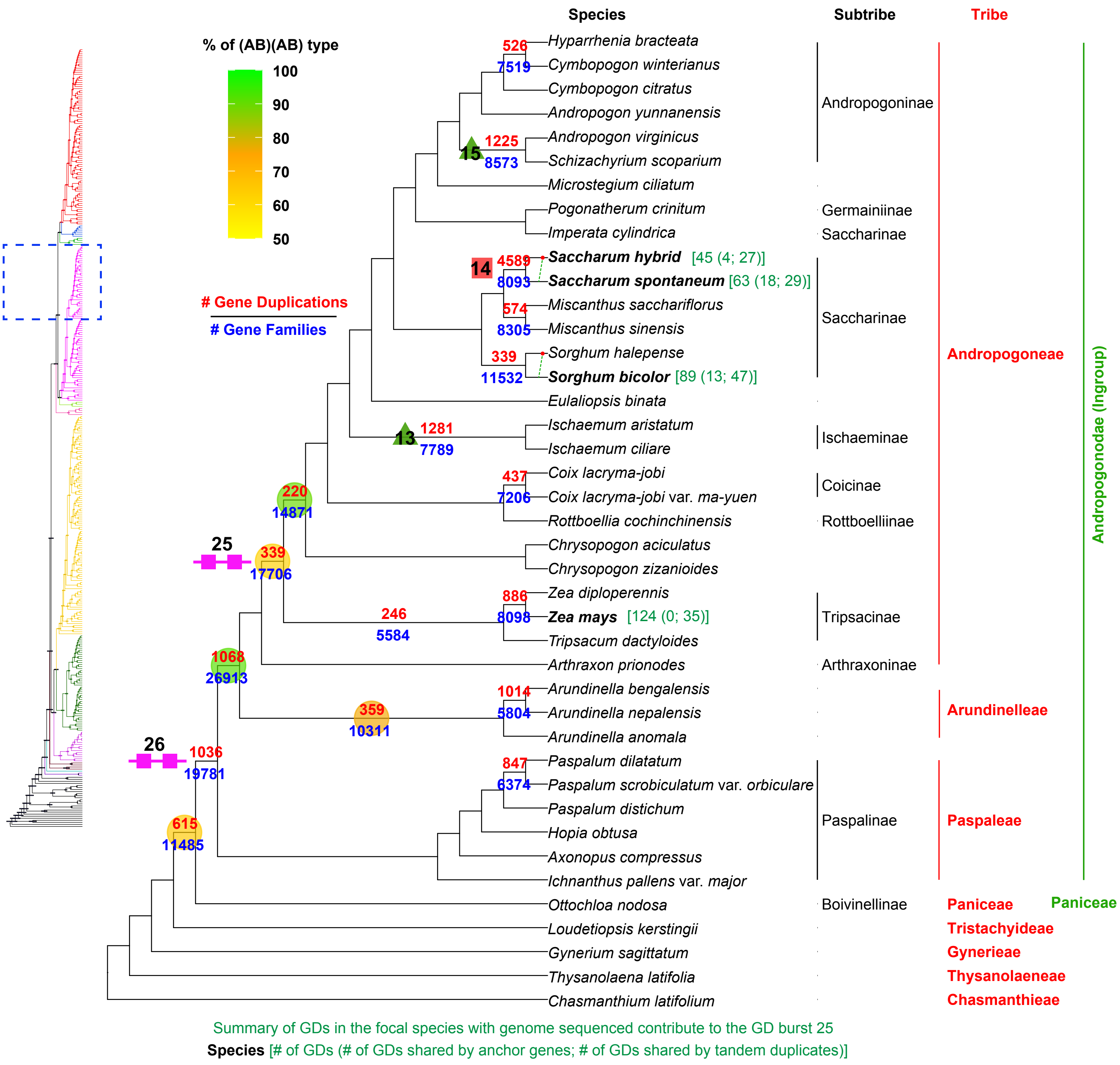

Supplementary Figure 13 WGDs and GD bursts in supertribe Andropogoneae of Panicoideae detected by phylogenomic analyses via Tree2GD

Meanings of numbers and symbols in the phylogeny are same as those in Supplementary Fig. 3. WGDs identified in this group include the *Ischaemum* WGD (#13), the *Saccharum* WGD (#14) and the WGD shared by *Andropogon virginicus* and *Schizachyrium scoparium* (#15). In addition, paralogs from *Sorghum bicolor* contribute to 89 GDs of 339 GDs mapped at the Andropogoneae tribe; among the 89 GDs, 13 GDs match syntenic gene pairs and 47 ones match tandem repeats. Analyses of tandem repeats mapped at Andropogoneae also detected 35 GDs from *Zea mays*, 29 GDs from *Saccharum spontaneum*, and 27 GDs from *Saccharum hybrid*. It seems possible that ancient SSD event (#25) likely contributed to the GD burst at Andropogoneae. Similarly, paralogs from *S. bicolor* contribute to 113 GDs of the cluster of 1036 GDs mapped at Andropogonodae and paralogs of tandem repeats correspond to 41 GDs of the 113 GDs, implying ancient SSD events at Andropogonodae (#26). Source data are provided as a Source Data file.

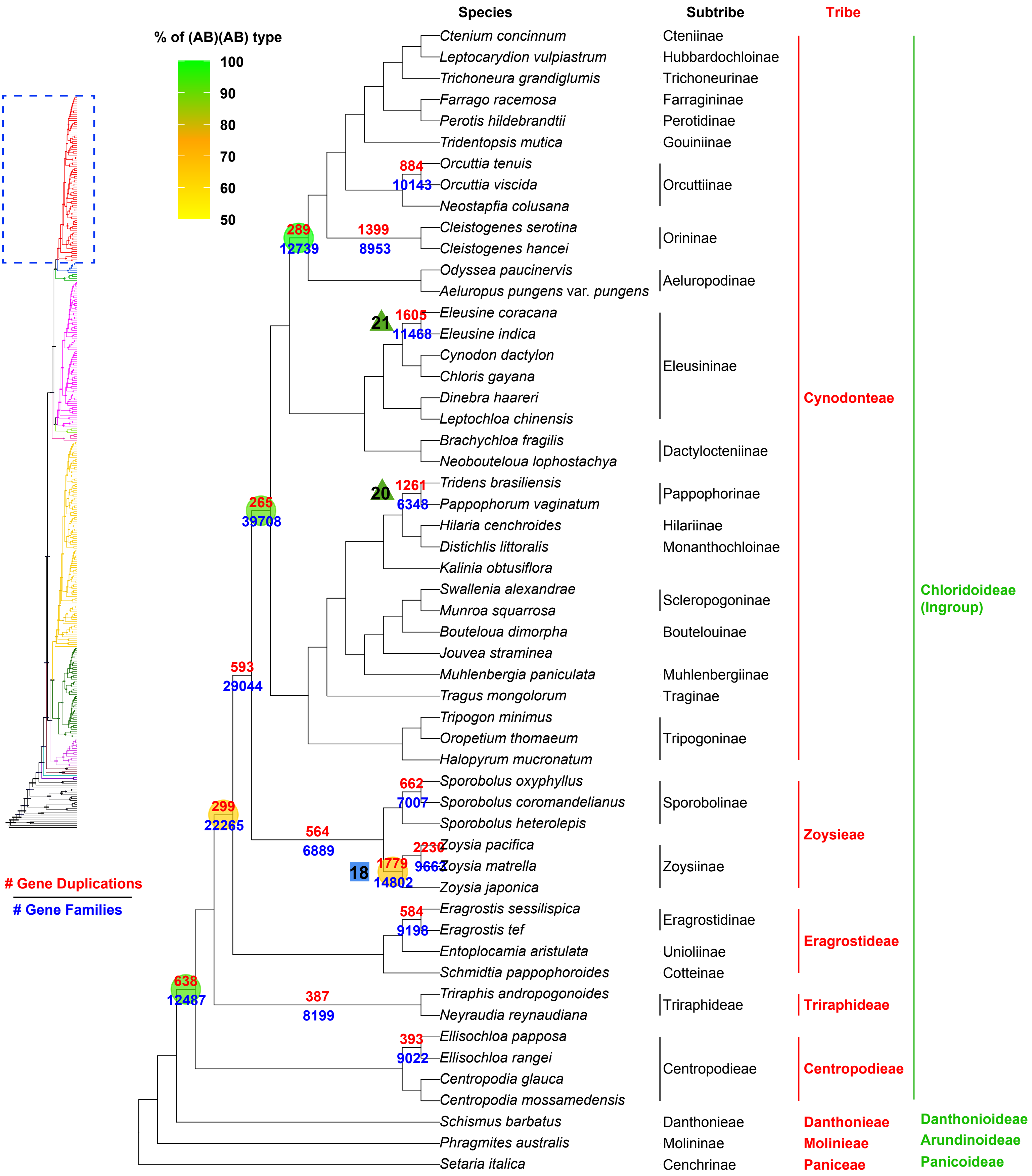

Supplementary Figure 14 WGDs and GD bursts in Chloridoideae detected by phylogenomic analyses via Tree2GD
Meanings of numbers and symbols in the phylogeny are same as those in Supplementary Fig. 3. WGDs detected in this group include the *Zoysia* WGD (#18), the WGD shared by *Tridens brasiliensis* and *Pappophorum vaginatum* (#20), and the *Eleusine* WGD (#21). Source data are provided as a Source Data file.

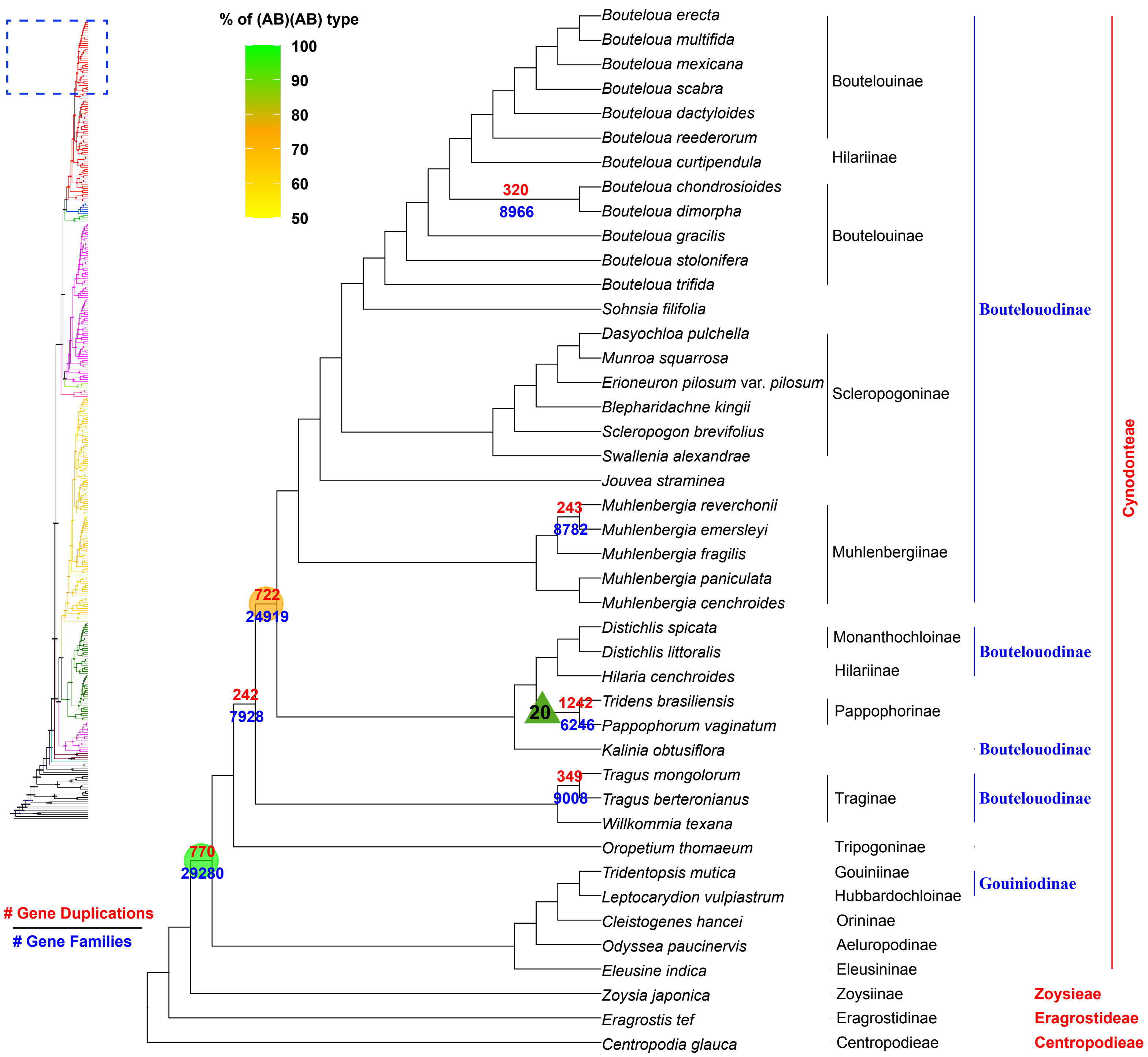

Supplementary Figure 15 WGD and GD bursts in supersubtribe Boutelouodinae (the Cynodonteae tribe of Chloridoideae) detected by phylogenomic analyses via Tree2GD

Meanings of numbers and symbols in the phylogeny are same as those in Supplementary Fig. 3. The WGD #20 in Supplementary Fig. 14 is also detected here. Source data are provided as a Source Data file.

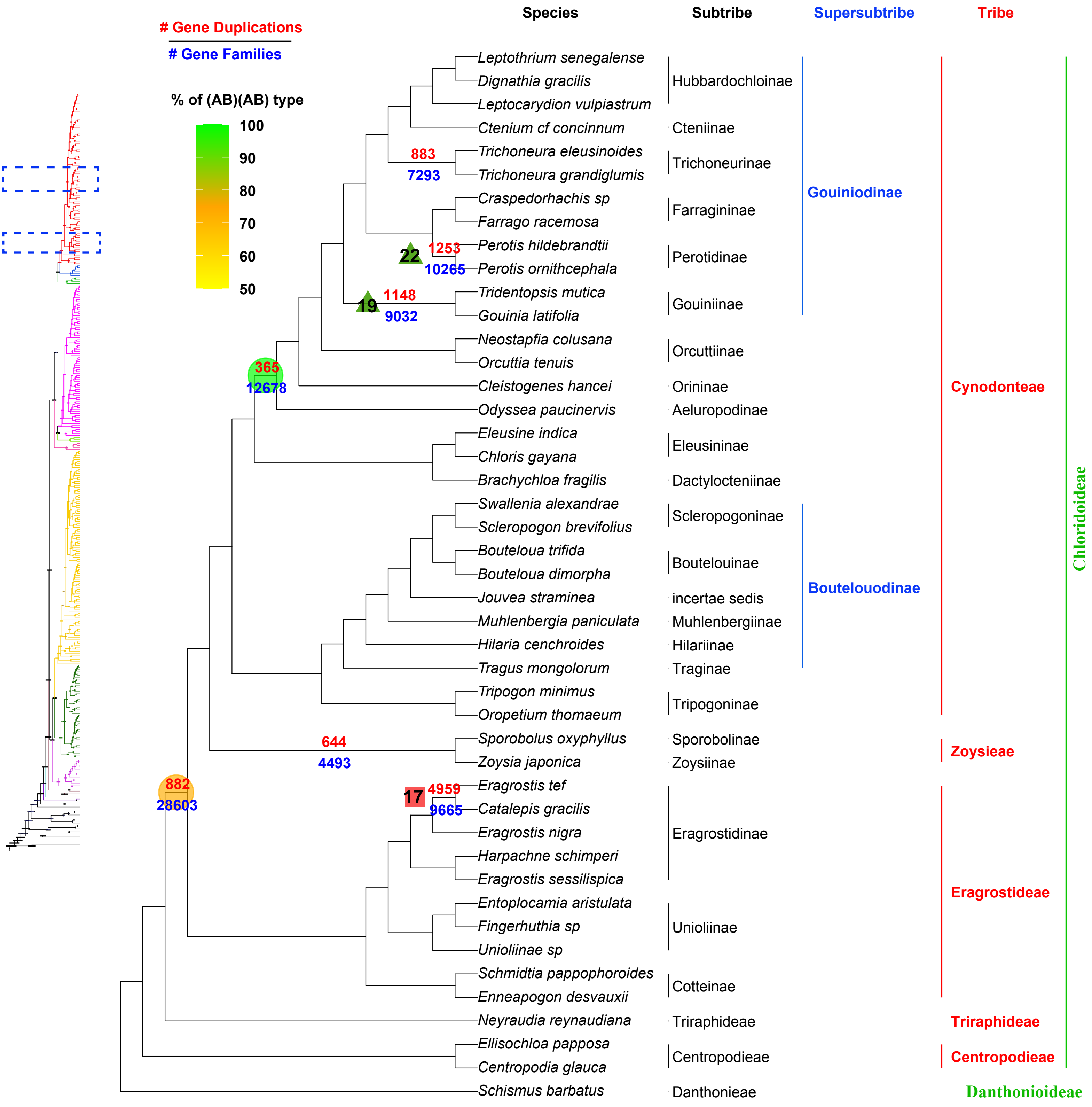

Supplementary Figure 16 WGDs and GD bursts in the supersubtribe Gouiniodinae of the Cynodonteae tribe and the Eragrostideae tribe of Chloridoideae detected by phylogenomic analyses via Tree2GD

Meanings of numbers and symbols in the phylogeny are same as those in Supplementary Fig. 3. Our GD clusters support *Eragrostis tef* and *Catalepis gracilis* WGD (#17), *Tridentopsis mutica* and *Gouinia latifolia* WGD (#19), and the *Perotis* WGD (#22). Source data are provided as a Source Data file.

a, Ks-plot for WGD1 (see details of GDs in Supplementary Fig. 3)

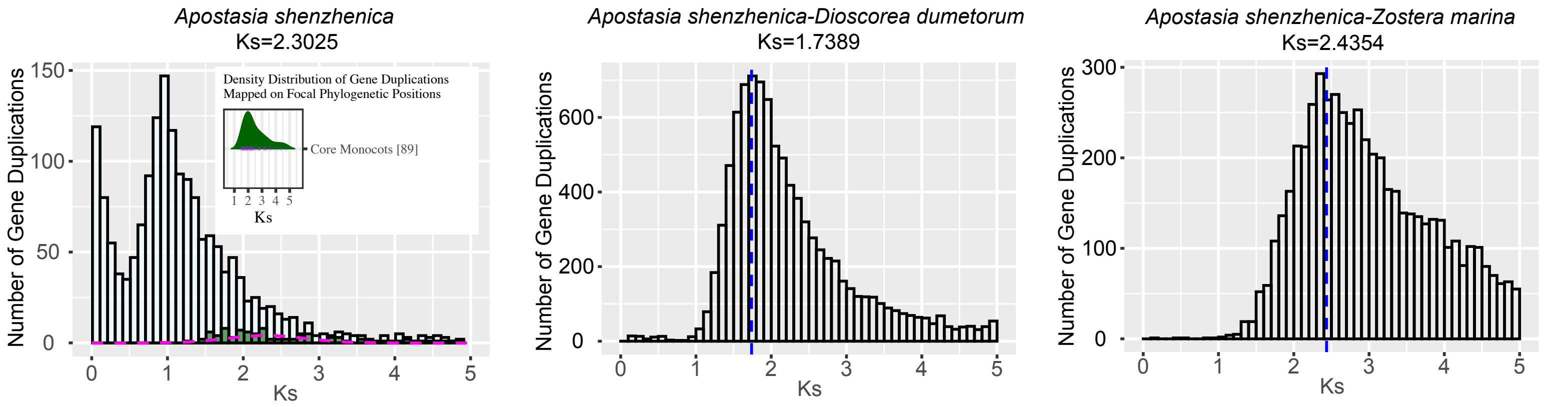

b, Ks-plot for WGD2 (see details of GDs in Supplementary Fig. 3)

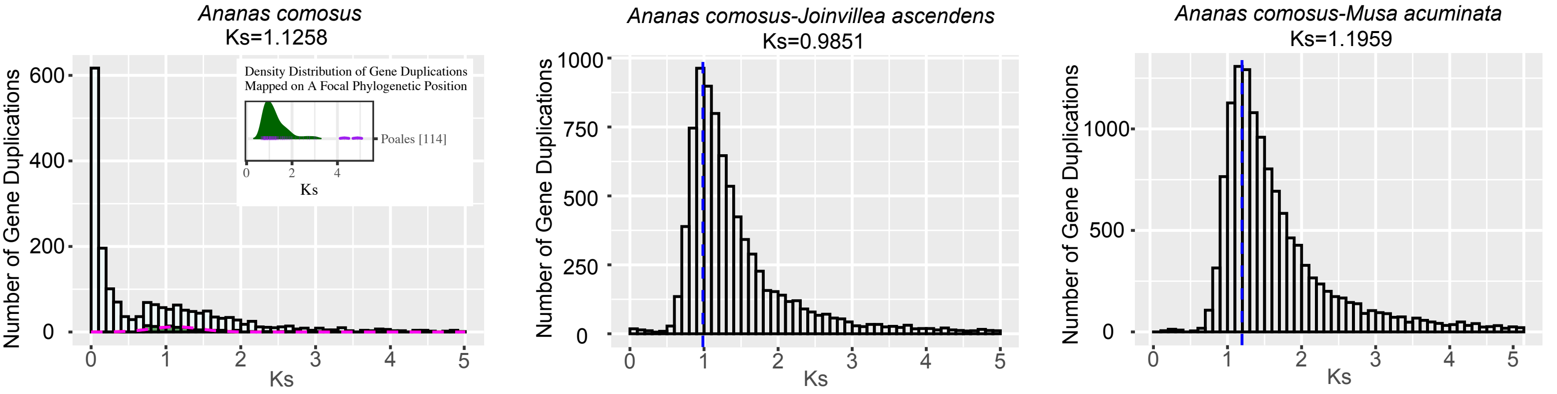

c, Ks-plot for WGD3 (see details of GDs in Supplementary Fig. 3)

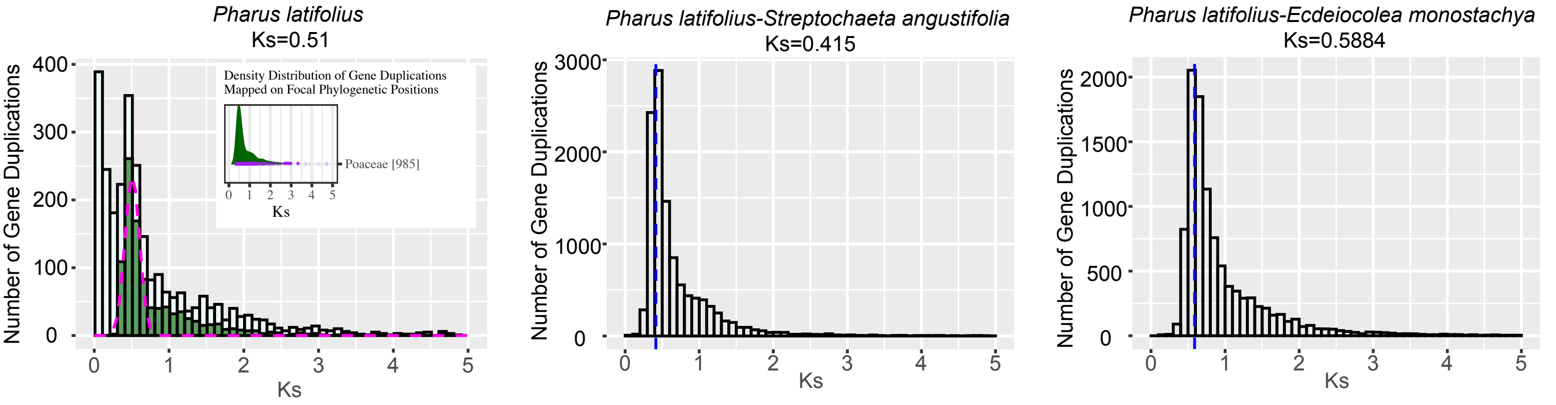

Supplementary Figure 17 Divergence time dating for 3 ancient WGD events, including tau (a), sigma (b), and rho (c)
For each part, Ks-plot showing the comparison of Ks-plot of WGD events consists of one Ks-Plot of paralogs (Left), two Ks-Plots of orthologs (Medium and Right). Bin width is set to 0.05 under a Ks range from 0 to 2, while it is set to 0.1 under a range from 0 to 5. In the left Ks-Plot, background white histogram represents the Ks distribution of gene duplications from all phylogenetic positions in our Tree2GD analyses, and foreground green histogram represents that from a focal phylogenetic position, with their density distribution, position and number (digital in square brackets) shown in top right, and with pink dash curve showing the peak of a focal WGD event. In the medium and right Ks-plots, the blue dash line represents the peak Ks value of orthologs. Source data are provided as a Source Data file.

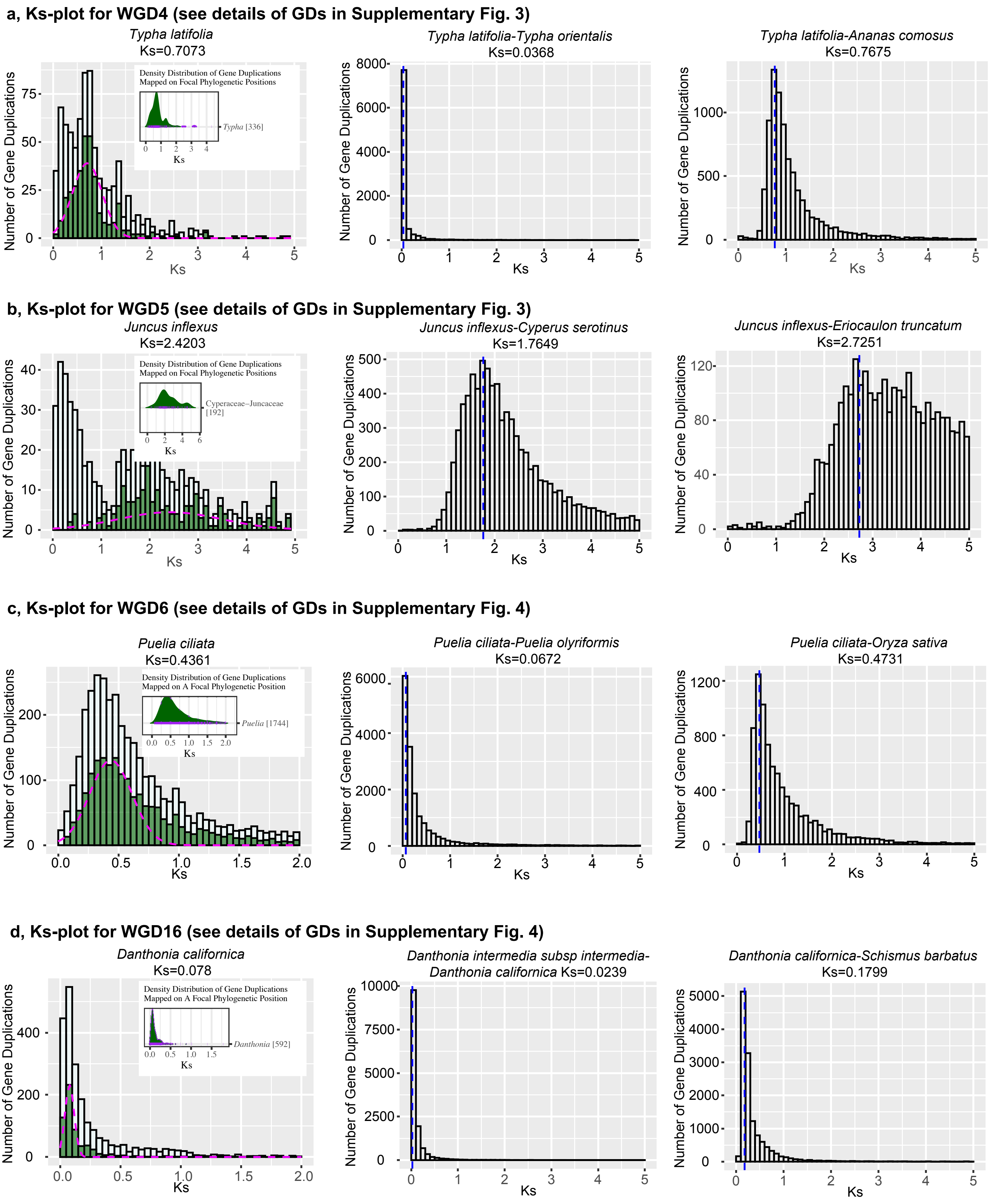

Supplementary Figure 18 Divergence time dating for WGD events #4 (a), #5 (b), #6 (c) and #16(d)
Color and meaning of the bars in Ks-Plot are same as that in Supplementary Fig. 17. Source data are provided as a Source Data file.

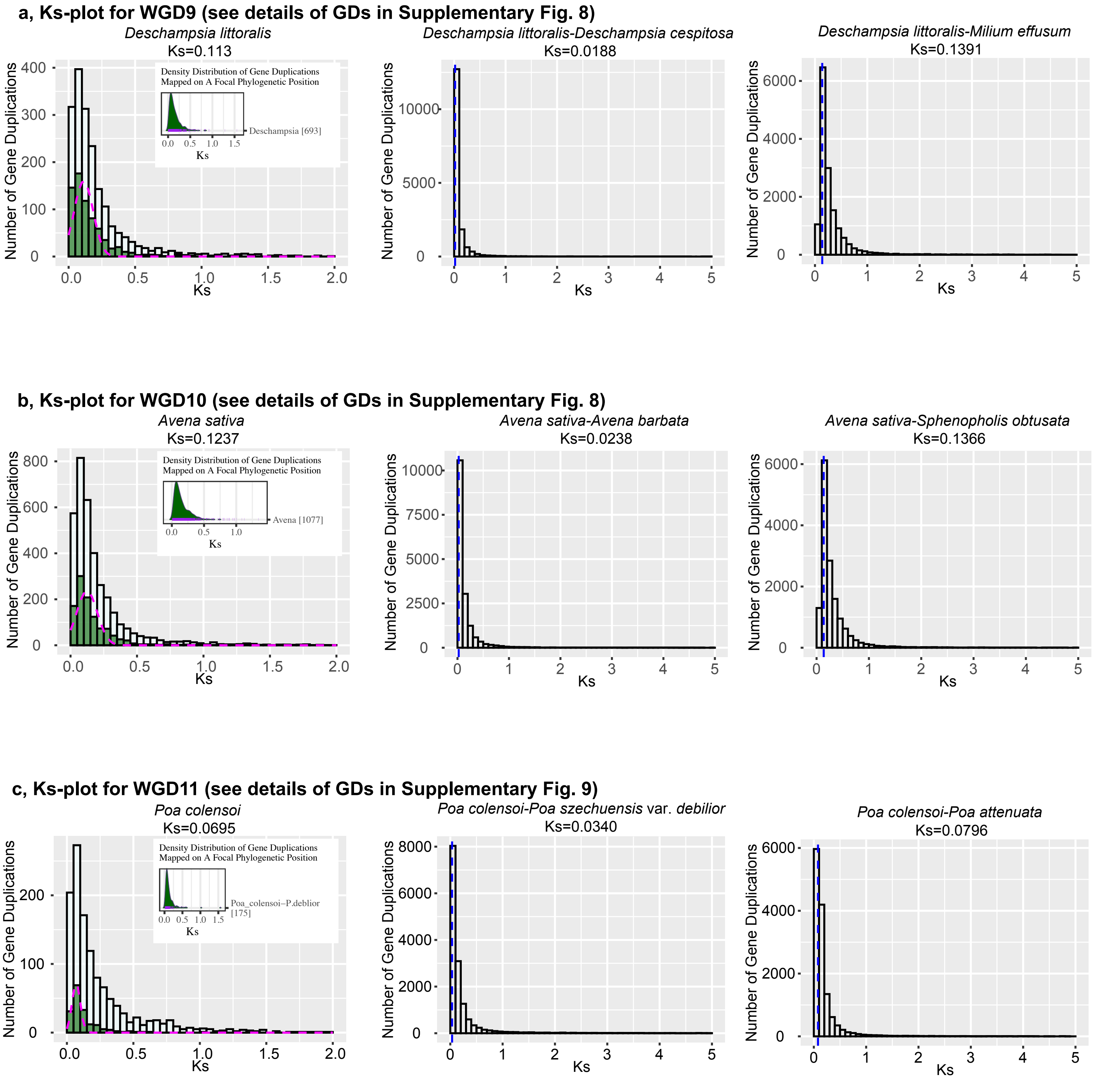

Supplementary Figure 19 Divergence time dating for WGD events #9 (a), #10 (b), and #11 (c) in Pooideae
Color and meaning of the bars in Ks-Plot are same as that in Supplementary Fig. 17. Source data are provided as a Source Data file.

a, Ks-plot for WGD7 (see details of GDs in Supplementary Fig. 6)

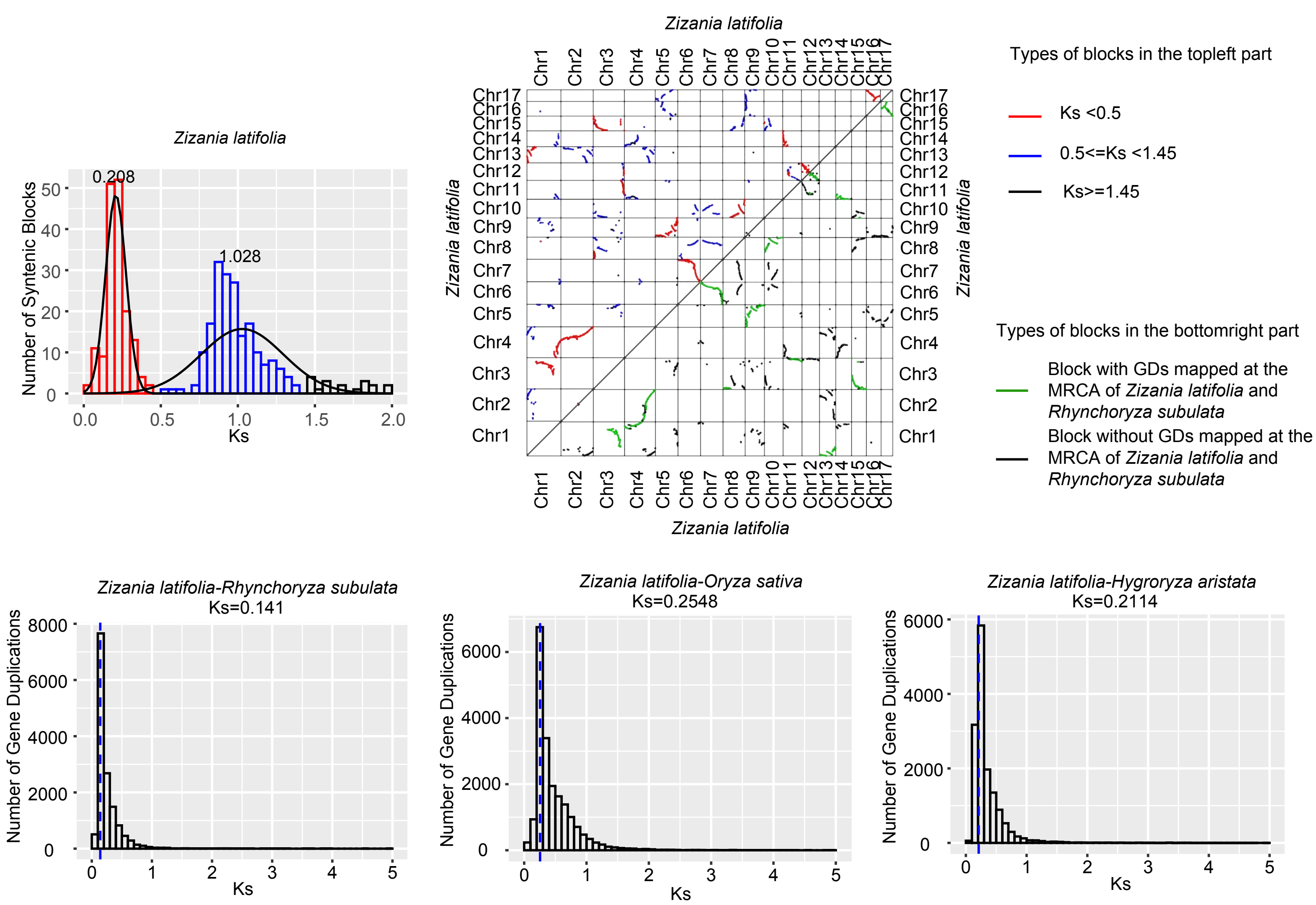

b, Ks-plot for WGD8 (see details of GDs in Supplementary Fig. 5)

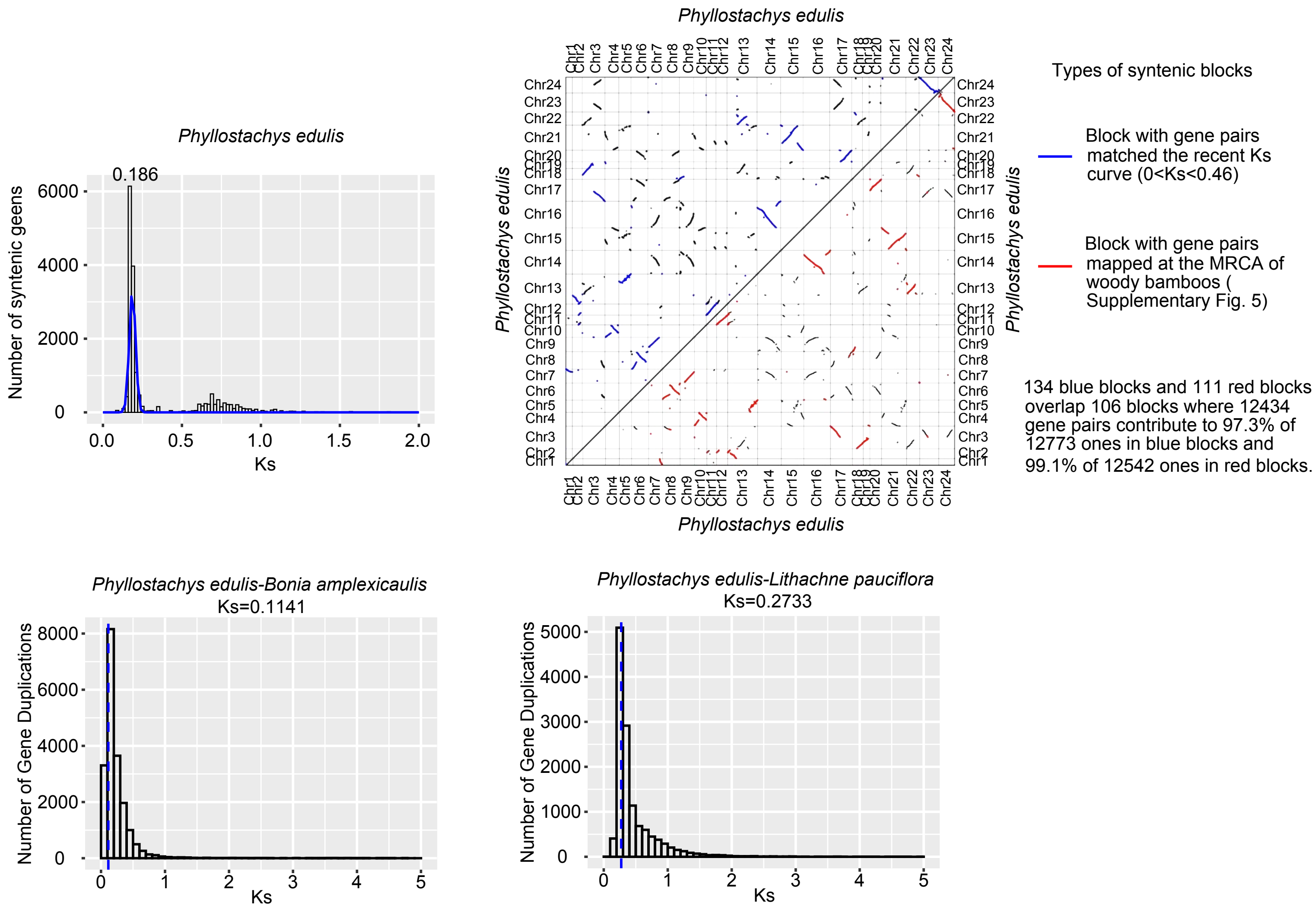

Supplementary Figure 20 Divergence time dating for WGD events #7 (a) and #8 (b)

(**a**) WGD shared by *Zizania latifolia* and *Rhynchoryza subulate*. Top-left: Ks-plot of syntenic blocks in *Z. latifolia* genome showing two clusters in the Ks range of 0~1.45. One cluster (red bars) locates in the Ks range of 0~0.5 with the peak at 0.208 and the other one (blue bars) in the Ks range of 0.5~1.45 with the peak at 1.028. Top-right: dot-plot of syntenic blocks in *Z. latifolia* genome, with color of syntenic blocks in the top-left part indicating their Ks range as shown in right, and the green blocks in the bottom-right part indicating some genes mapped at the MRCA of *Z. latifolia* and *R. subulate*. Bottom: Ks-plots of orthologs. See the color and meaning of the bars in Supplementary Fig. 17. (**b**) WGD in *Phyllostachys edulis* genome. Top-left: Ks-plot of syntenic genes in *P. edulis* genome showing a cluster of syntenic genes in the Ks range of 0~0.46 with a peak at 0.186. Top-right: dot-plot of syntenic blocks in *P. edulis* genome, with the blue syntenic blocks in the top-left part indicating their Ks range as shown in right, and the red blocks in the bottom-right part indicating some genes mapped at the MRCA of woody bamboos. Bottom: Ks-plots of orthologs. See the color and meaning of the bars in Supplementary Fig. 17. Source data are provided as a Source Data file.

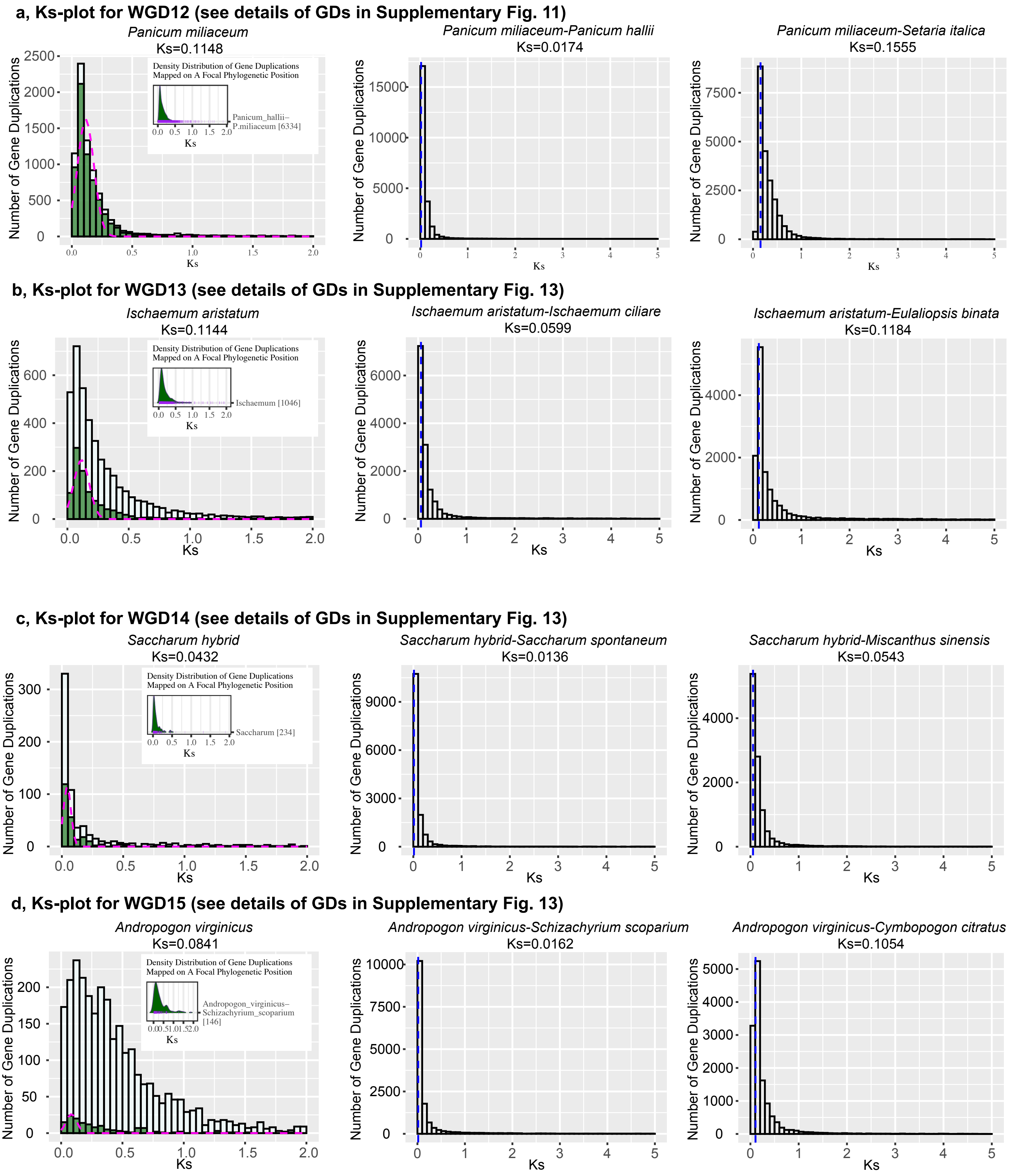

Supplementary Figure 21 Divergence time dating for WGD events #12 (a), #13 (b), #14 (c), and #15 (d) in Panicoideae
Color and meaning of the bars in Ks-Plot are same as that in Supplementary Fig. 17. Source data are provided as a Source Data file.

a, Ks-plot for WGD17 (see details of GDs in Supplementary Fig. 16)

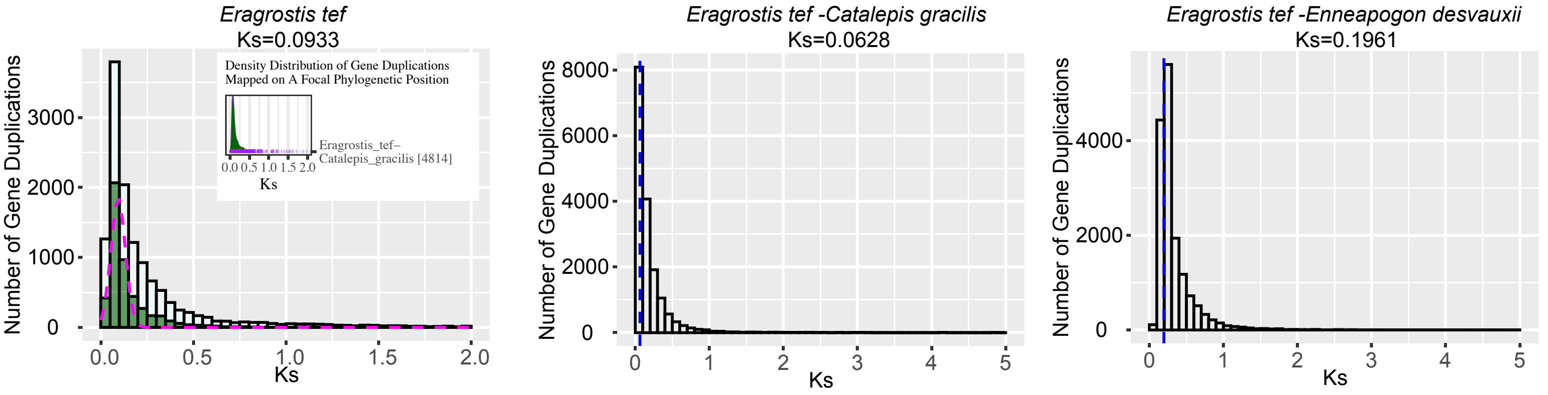

b, Ks-plot for WGD18 (see details of GDs in Supplementary Fig. 14)

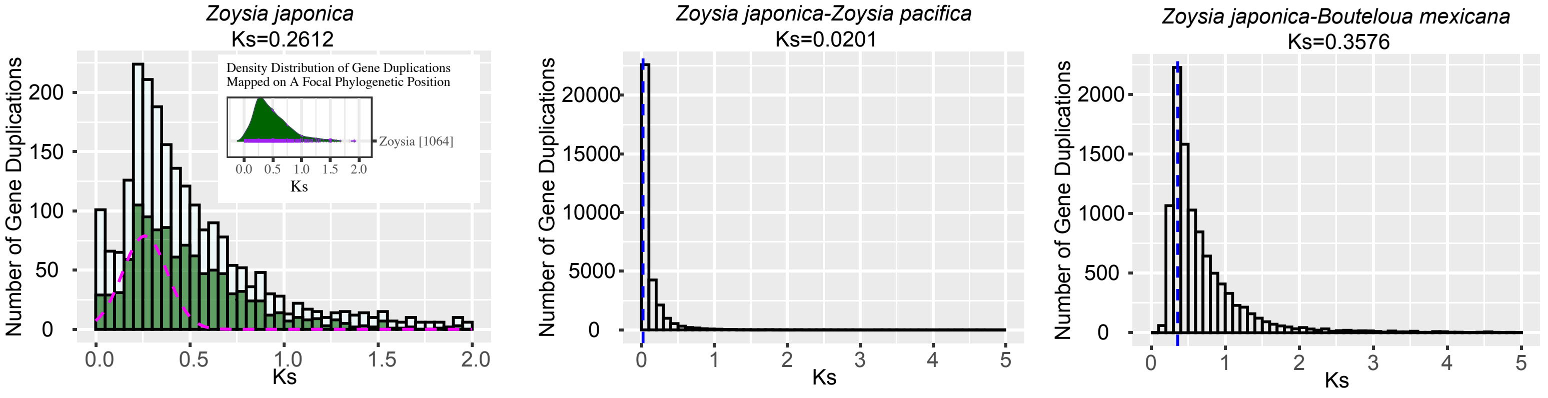

c, Ks-plot for WGD19 (see details of GDs in Supplementary Fig. 16)

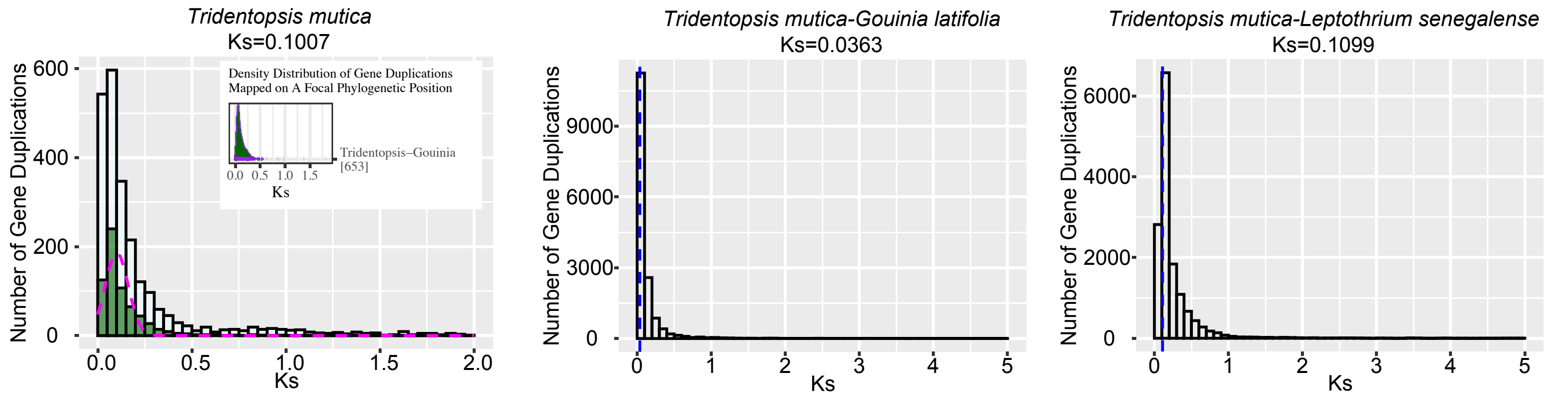

Supplementary Figure 22 Divergence time dating for WGD events #17 (a), #18 (b), and #19 (c) in Chloridoideae
Color and meaning of the bars in Ks-Plot are same as that in Supplementary Fig. 17. Source data are provided as a Source Data file.

a, Ks-plot for WGD20 (see details of GDs in Supplementary Fig. 14)

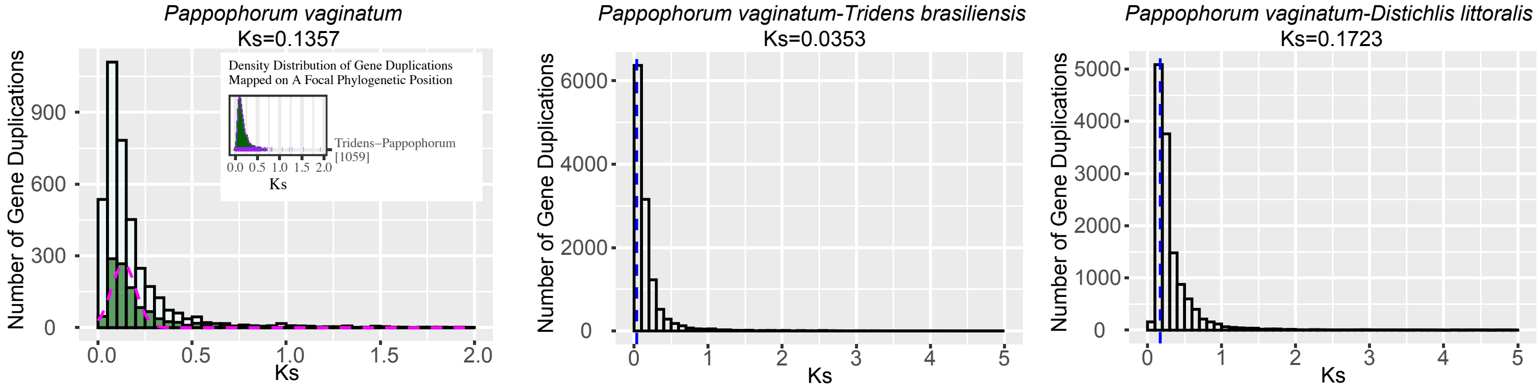

b, Ks-plot for WGD21 (see details of GDs in Supplementary Fig. 14)

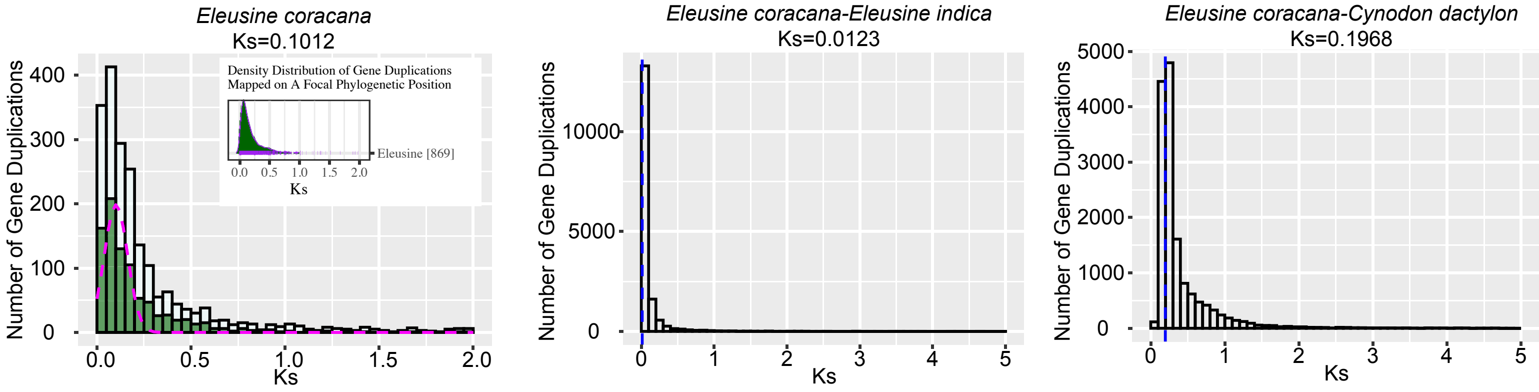

c, Ks-plot for WGD22 (see details of GDs in Supplementary Fig. 16)

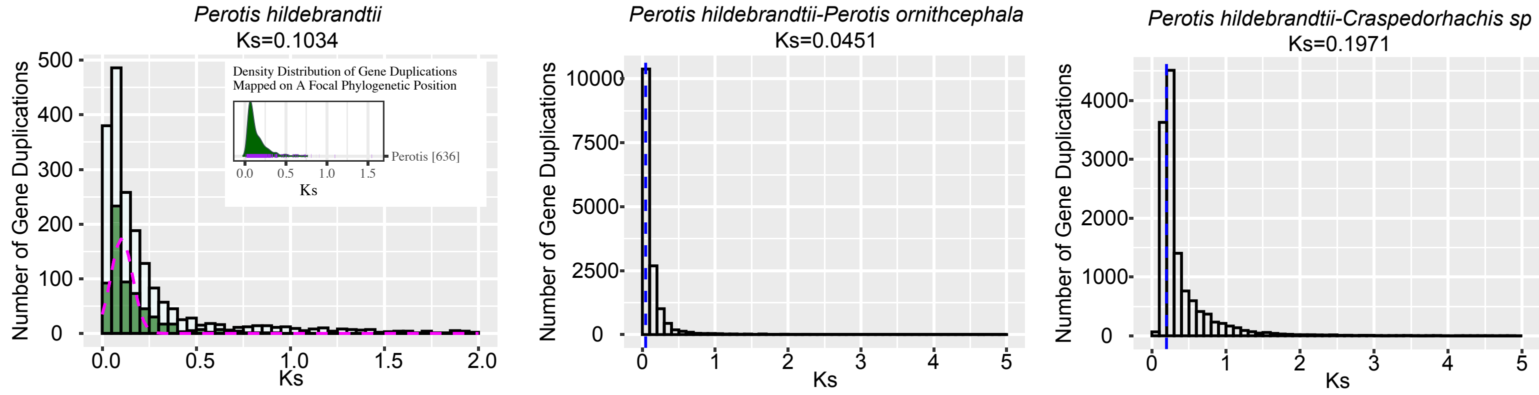

Supplementary Figure 23 Divergence time dating for WGD events #20 (a), #21 (b), and #22 (c) in Chloridoideae
Color and meaning of the bars in Ks-Plot are same as that in Supplementary Fig. 17. Source data are provided as a Source Data file.

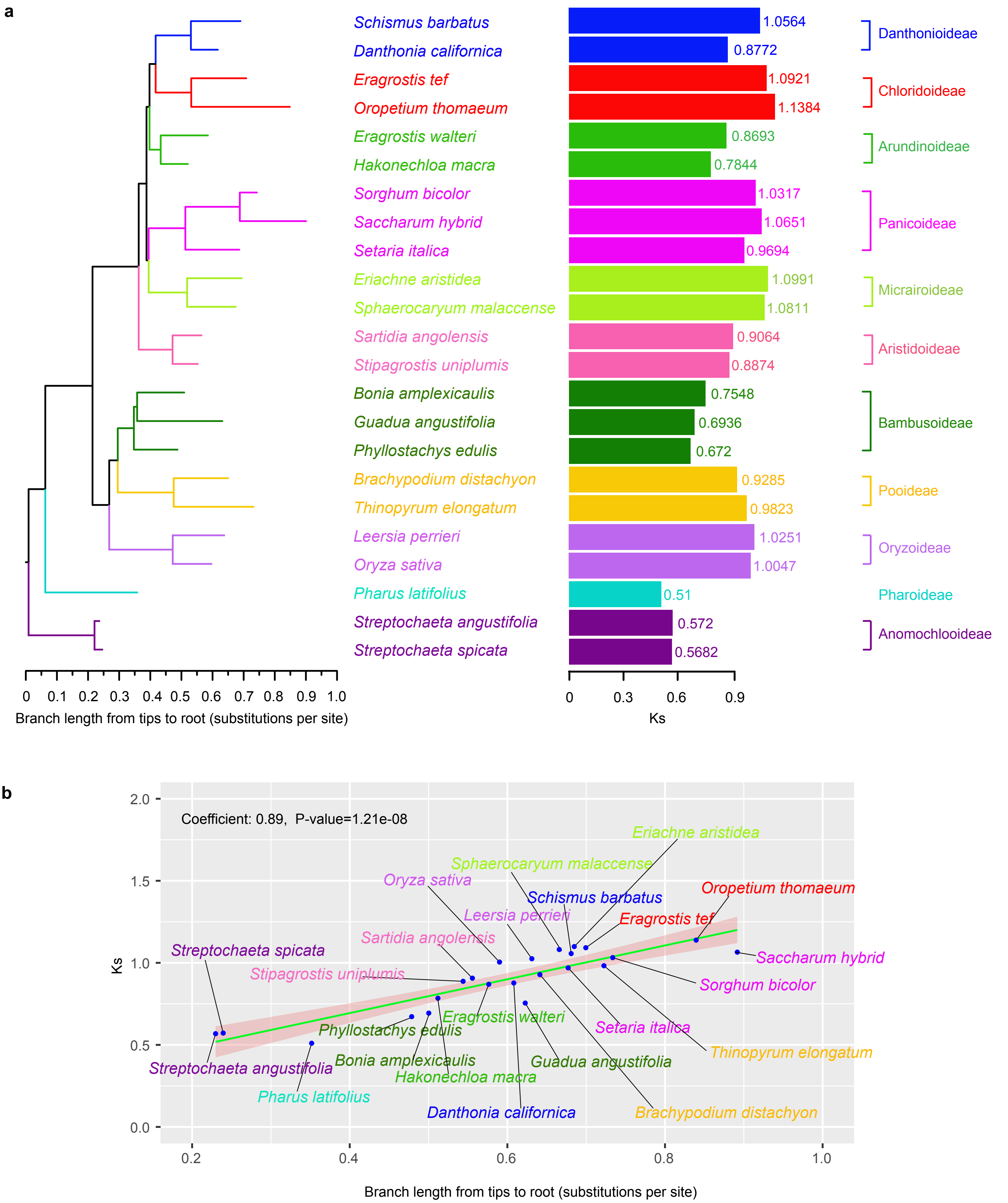

Supplementary Figure 24 Divergence time of duplicates from rho retained in different species varied with different divergence rate

(**a**) Illustration of phylogenetic relationships among Poaceae (a part of the specie-tree in Supplementary Fig. 1a) with right bar-plot showing the Ks value (number) of paralogs mapped at Poaceae from the responding species. Scale below the tree represents the branch length from tips to root. (**b**) Coefficient analysis between the Ks and branch length from tips to root. P-value was estimated by cor.test (method = "pearson", alternative ="two.sided") in R. n=23 species. Source data are provided as a Source Data file.

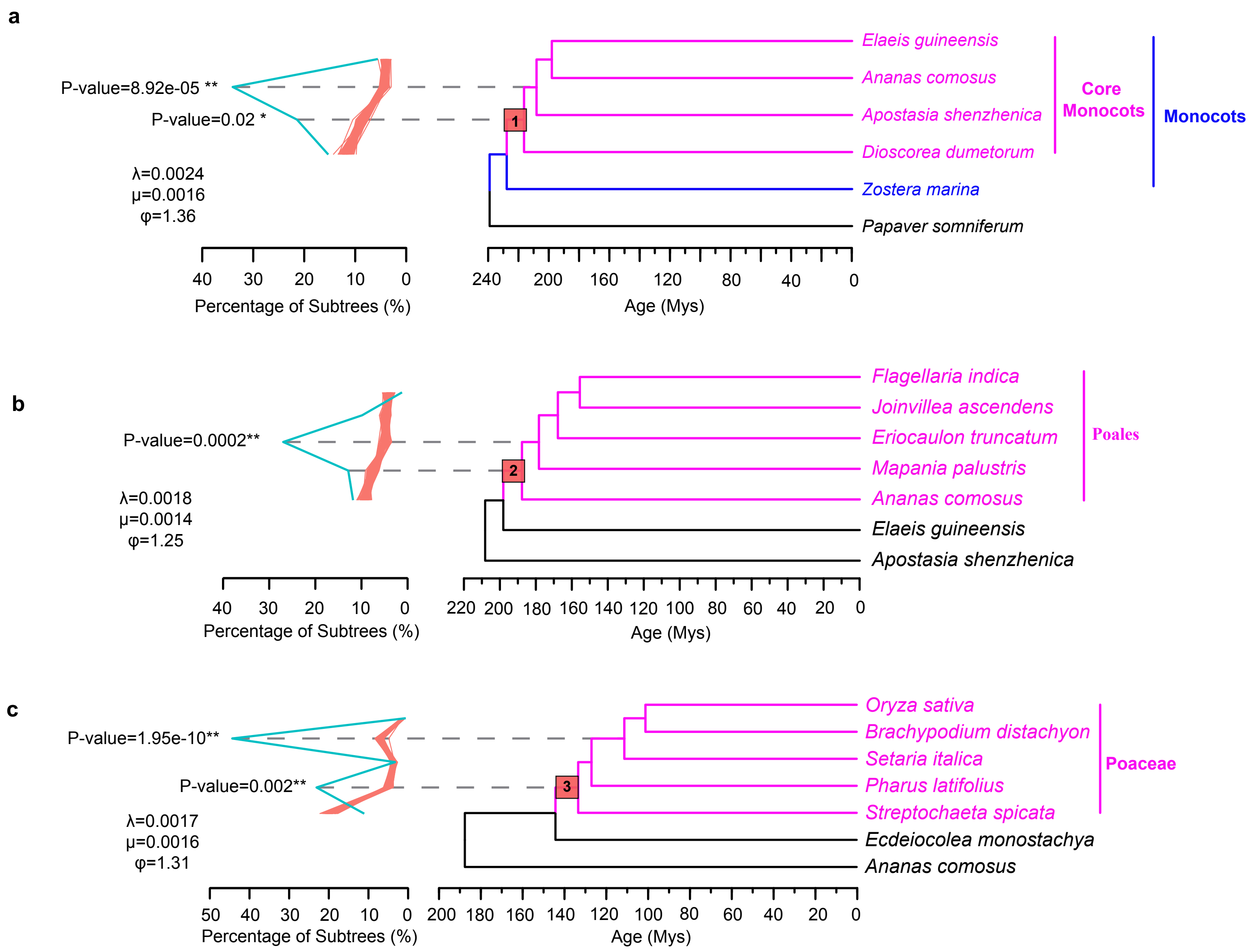

Supplementary Figure 25 Estimation of gene counts from rho, sigma, and tau events with gene-tree reconciliation via MAPS analyses.

Left graph shows the percentage of gene duplications mapped at different nodes of right species tree that simplified from Supplementary Fig. 2. Green lines represent the duplication percentage mapping from true gene families on the species tree and red lines represent that from null simulation. The null simulation involves the estimation of gene birth (λ) and death (μ) rates from true gene families and simulation of gene trees with the λ and μ under an assumption that only small-scale duplations but not WGDs shaped the gene evolution. Asterisks (*) superimposed on the green line indicate that the phylogenomic results of true data extremely significantly (p-value < 0.001) higher than that of null simulation. P-value was estimated by fisher.test (alternative="greater") in R. Red squares at the nodes are same as those in Figure 1. (**a**) MAPS result of tau event (#1). (**b**) MAPS result of sigma event (#2). (**c**) MAPS result of rho event (#3). All three ancient WGDs are mapped by peaks of duplication percentage distribution from true gene families and obtain extremely significant duplication percentage compared to small-scale duplications mapped at respective node. Although strong evidence supports rho event mapped at Poaceae, another strong signal is mapped at the core Poaceae, owing to small-scale duplications but not a WGD contributing to the duplication burst mapped at the core Poaceae. However, here the small-scale duplications can only count a part of the duplication burst mapped at the core Poaceae. Hence more analyses need to explore gene evolution pattern for successive duplication bursts. Source data are provided as a Source Data file.

a

Summary of GDs shared by paralogs in each species mapped at Poaceae

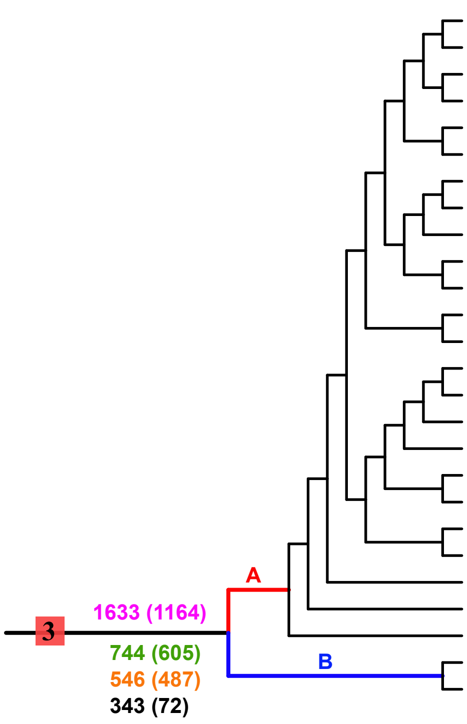

Species	Number of GDs in different retention type shared by paralogs in each species				Subfamily
	Total	(AB)(AB)	(AB)A	(AB)B	
Schismus barbatus	593	328	265	-	Danthonioideae
Danthonia californica	347	204	143	-	
Eragrostis tef	820 (191)	439 (96)	381 (95)	-	Chloridoideae
Oropetium thomaeum	423 (329)	241 (183)	182 (146)	-	
Arundo donax	490	266	224	-	Arundinoideae
Hakonechloa macra	401	220	181	-	
Sorghum bicolor	840 (623)	459 (334)	381 (289)	-	Panicoideae
Saccharum hybrid	272 (160)	158 (95)	114 (65)	-	
Setaria italica	868 (640)	471 (334)	397 (306)	-	
Eriachne aristidea	313	185	128	-	Micrairoideae
Sphaerocaryum malaccense	315	166	149	-	
Sartidia angolensis	413	230	183	-	Aristidoideae
Stipagrostis uniplumis	372	200	172	-	
Bonia amplexicaulis	861 (410)	457 (222)	404 (188)	-	Bambusoideae
Guadua angustifolia	518	280	238	-	
Phyllostachys edulis	1023 (631)	539 (324)	484 (307)	-	
Olyra latifolia	818 (531)	452 (292)	366 (239)	-	
Brachypodium distachyon	772 (582)	425 (322)	347 (260)	-	Pooideae
Thinopyrum elongatum	691 (337)	376 (178)	315 (159)	-	
Leersia perrieri	732 (577)	386 (295)	346 (282)	-	Oryzoideae
Oryza sativa	847 (622)	460 (327)	387 (295)	-	
Puelia ciliata	36	26	10	-	Puelioideae I
Guaduella oblonga	19	15	4	-	Puelioideae II
Pharus latifolius	1018 (811)	472 (377)	546 (434)	-	Pharoideae
Streptochaeta angustifolia	1010 (201)	667 (129)	-	343 (72)	Anomochlooideae
Streptochaeta spicata	728	385	-	343	

# of GDs (# of GDs matched by syntenic genes)

b

Summary of GDs matched by syntenic genes mapped at Poaceae

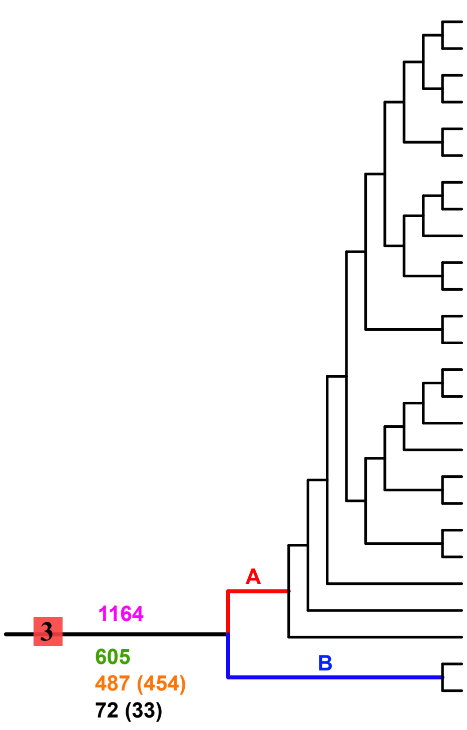

Species	Number of GDs in different retention type matched by syntenic genes in each species				Subfamily
	Total	(AB)(AB)	(AB)A	(AB)B	
Schismus barbatus	-	-	-	-	Danthonioideae
Danthonia californica	-	-	-	-	
Eragrostis tef	191	96	95 (57)	-	Chloridoideae
Oropetium thomaeum	329	183	146 (116)	-	
Arundo donax	-	-	-	-	Arundinoideae
Hakonechloa macra	-	-	-	-	
Sorghum bicolor	623	334	289 (256)	-	Panicoideae
Saccharum hybrid	160	95	65 (41)	-	
Setaria italica	640	334	306 (275)	-	
Eriachne aristidea	-	-	-	-	Micrairoideae
Sphaerocaryum malaccense	-	-	-	-	
Sartidia angolensis	-	-	-	-	Aristidoideae
Stipagrostis uniplumis	-	-	-	-	
Bonia amplexicaulis	410	222	188(150)	-	Bambusoideae
Guadua angustifolia	-	-	-	-	
Phyllostachys edulis	631	324	307 (272)	-	
Olyra latifolia	531	292	239 (175)	-	
Brachypodium distachyon	582	322	260 (228)	-	Pooideae
Thinopyrum elongatum	337	178	159 (119)	-	
Leersia perrieri	577	295	282 (252)	-	Oryzoideae
Oryza sativa	622	327	295 (257)	-	
Puelia ciliata	-	-	-	-	Puelioideae I
Guaduella oblonga	-	-	-	-	Puelioideae II
Pharus latifolius	811	377	434 (411)	-	Pharoideae
Streptochaeta angustifolia	201	129	-	72 (33)	Anomochlooideae
Streptochaeta spicata	-	-	-	-	

# of GDs matched by syntenic genes (# of GDs in a synteny block included other GDs retained in (AB)(AB) type)

c

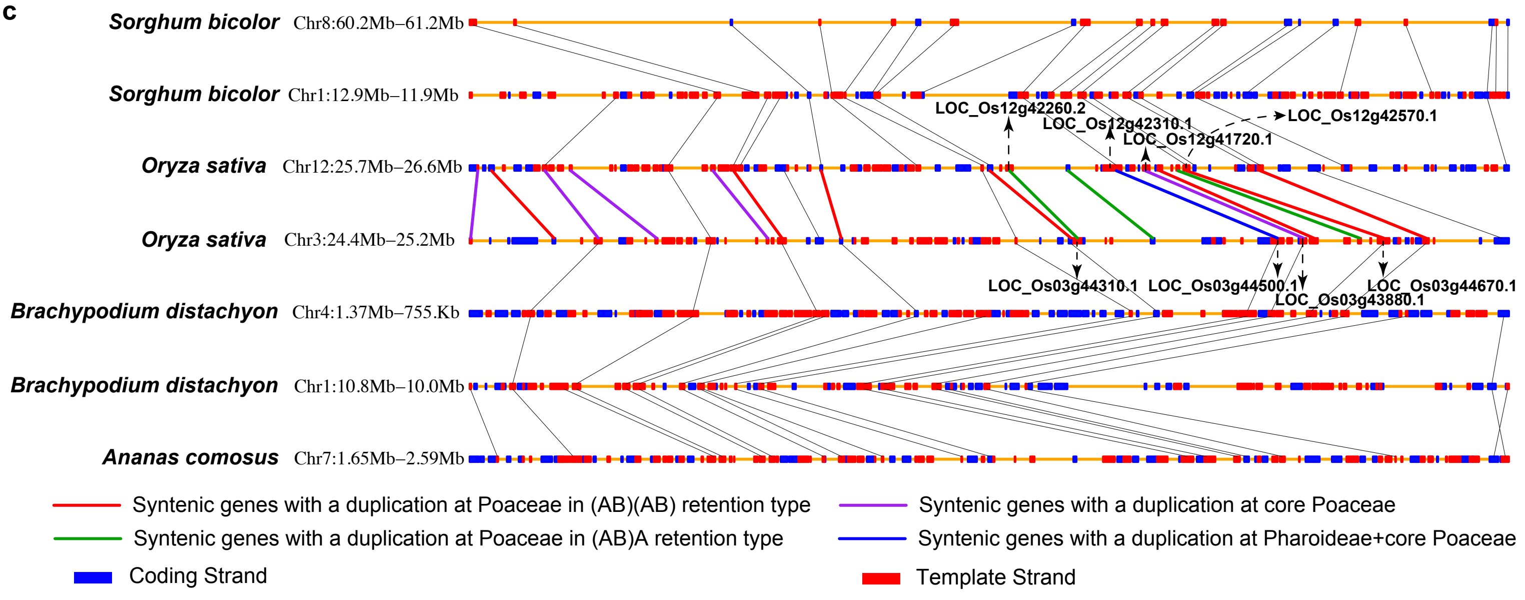

d

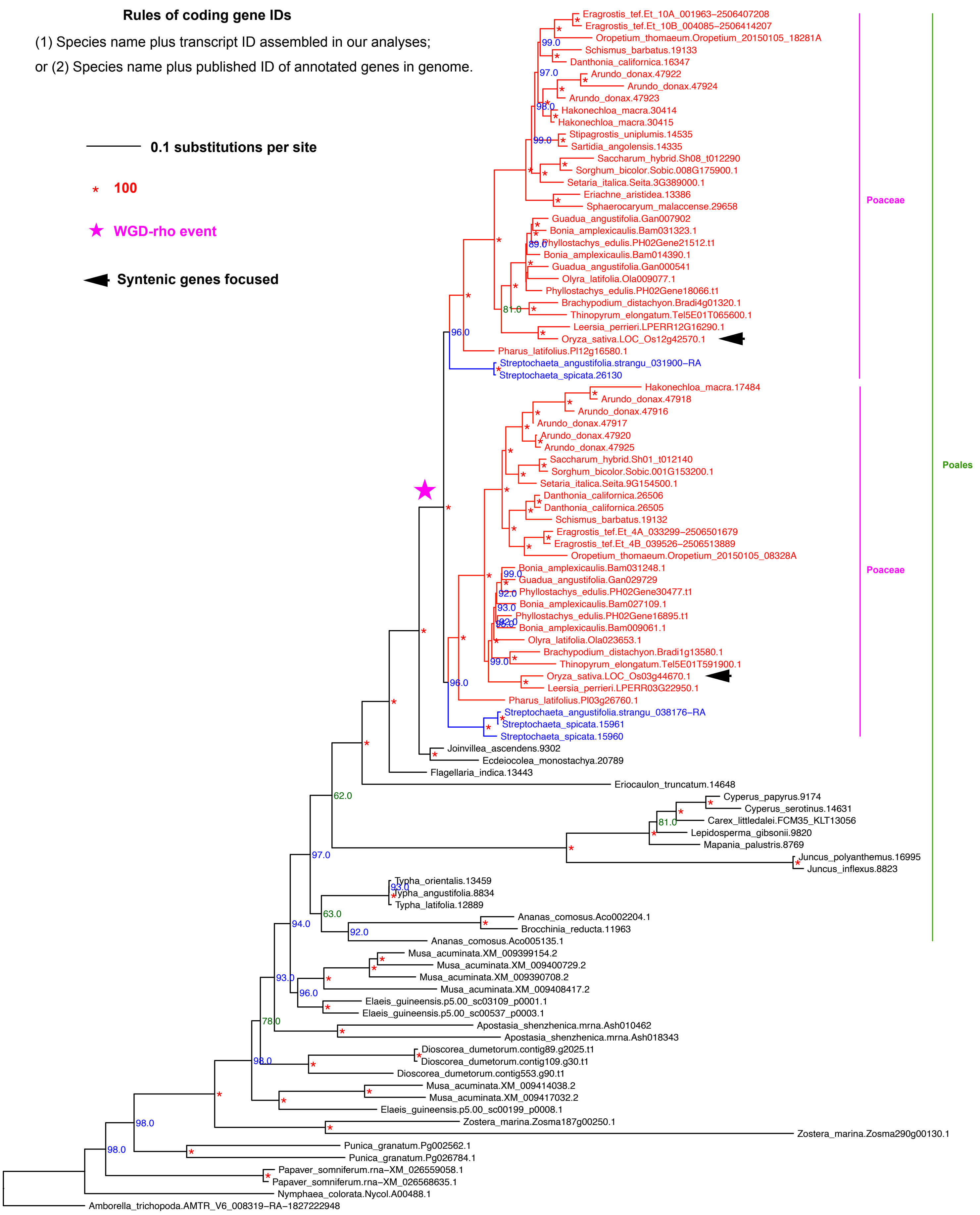
Rules of coding gene IDs
(1) Species name plus transcript ID assembled in our analyses;
or (2) Species name plus published ID of annotated genes in genome.
0.1 substitutions per site
* 100
★ WGD-rho event
Syntenic genes focused
Eragrostis_tef.Et_10A_001963−2506407208
Eragrostis_tef.Et_10B_004085−2506414207
Oropetium_thomaeum.Oropetium_20150105_18281A
Schismus_barbatus.19133
Danthonia_californica.16347
Arundo_donax.47922
Arundo_donax.47924
Arundo_donax.47923
Hakonechloa_macra.30414
Hakonechloa_macra.30415
Stipagrostis_uniplumis.14535
Sartidia_angolensis.14335
Saccharum_hybrid.Sh08_t012290
Sorghum_bicolor.Sobic.008G175900.1
Setaria_italica.Seita.3G389000.1
Eriachne_aristidea.13386
Sphaerocaryum_malaccense.29658
Guadua_angustifolia.Gan007902
Bonia_amplexicaulis.Bam031323.1
Phyllostachys_edulis.PH02Gene21512.t1
Bonia_amplexicaulis.Bam014390.1
Guadua_angustifolia.Gan000541
Olyra_latifolia.Ola009077.1
Phyllostachys_edulis.PH02Gene18066.t1
Brachypodium_distachyon.Bradi4g01320.1
Thinopyrum_elongatum.Tel5E01T065600.1
Leersia_perrieri.LPERR12G16290.1
Oryza_sativa.LOC_Os12g42570.1
Pharus_latifolius.Pl12g16580.1
Streptochaeta_angustifolia.strangu_031900−RA
Streptochaeta_spicata.26130
Hakonechloa_macra.17484
Arundo_donax.47918
Arundo_donax.47916
Arundo_donax.47917
Arundo_donax.47920
Arundo_donax.47925
Saccharum_hybrid.Sh01_t012140
Sorghum_bicolor.Sobic.001G153200.1
Setaria_italica.Seita.9G154500.1
Danthonia_californica.26506
Danthonia_californica.26505
Schismus_barbatus.19132
Eragrostis_tef.Et_4A_033299−2506501679
Eragrostis_tef.Et_4B_039526−2506513889
Oropetium_thomaeum.Oropetium_20150105_08328A
Bonia_amplexicaulis.Bam031248.1
Guadua_angustifolia.Gan029729
Phyllostachys_edulis.PH02Gene30477.t1
Bonia_amplexicaulis.Bam027109.1
Phyllostachys_edulis.PH02Gene16895.t1
Bonia_amplexicaulis.Bam009061.1
Olyra_latifolia.Ola023653.1
Brachypodium_distachyon.Bradi1g13580.1
Thinopyrum_elongatum.Tel5E01T591900.1
Oryza_sativa.LOC_Os03g44670.1
Leersia_perrieri.LPERR03G22950.1
Pharus_latifolius.Pl03g26760.1
Streptochaeta_angustifolia.strangu_038176−RA
Streptochaeta_spicata.15961
Streptochaeta_spicata.15960
Joinvillea_ascendens.9302
Ecdeiocolea_monostachya.20789
Flagellaria_indica.13443
Eriocaulon_truncatum.14648
Cyperus_papyrus.9174
Cyperus_serotinus.14631
Carex_littledalei.FCM35_KLT13056
Lepidosperma_gibsonii.9820
Mapania_palustris.8769
Juncus_polyanthemus.16995
Juncus_inflexus.8823
Typha_orientalis.13459
Typha_angustifolia.8834
Typha_latifolia.12889
Ananas_comosus.Aco002204.1
Brocchinia_reducta.11963
Ananas_comosus.Aco005135.1
Musa_acuminata.XM_009399154.2
Musa_acuminata.XM_009400729.2
Musa_acuminata.XM_009390708.2
Musa_acuminata.XM_009408417.2
Elaeis_guineensis.p5.00_sc03109_p0001.1
Elaeis_guineensis.p5.00_sc00537_p0003.1
Apostasia_shenzhenica.mrna.Ash010462
Apostasia_shenzhenica.mrna.Ash018343
Dioscorea_dumetorum.contig89.g2025.t1
Dioscorea_dumetorum.contig109.g30.t1
Dioscorea_dumetorum.contig553.g90.t1
Musa_acuminata.XM_009414038.2
Musa_acuminata.XM_009417032.2
Elaeis_guineensis.p5.00_sc00199_p0008.1
Zostera_marina.Zosma187g00250.1
Zostera_marina.Zosma290g00130.1
Punica_granatum.Pg002562.1
Punica_granatum.Pg026784.1
Papaver_somniferum.rna−XM_026559058.1
Papaver_somniferum.rna−XM_026568635.1
Nymphaea_colorata.Nycol.A00488.1
Amborella_trichopoda.AMTR_V6_008319−RA−1827222948
Poaceae
Poaceae
Poales

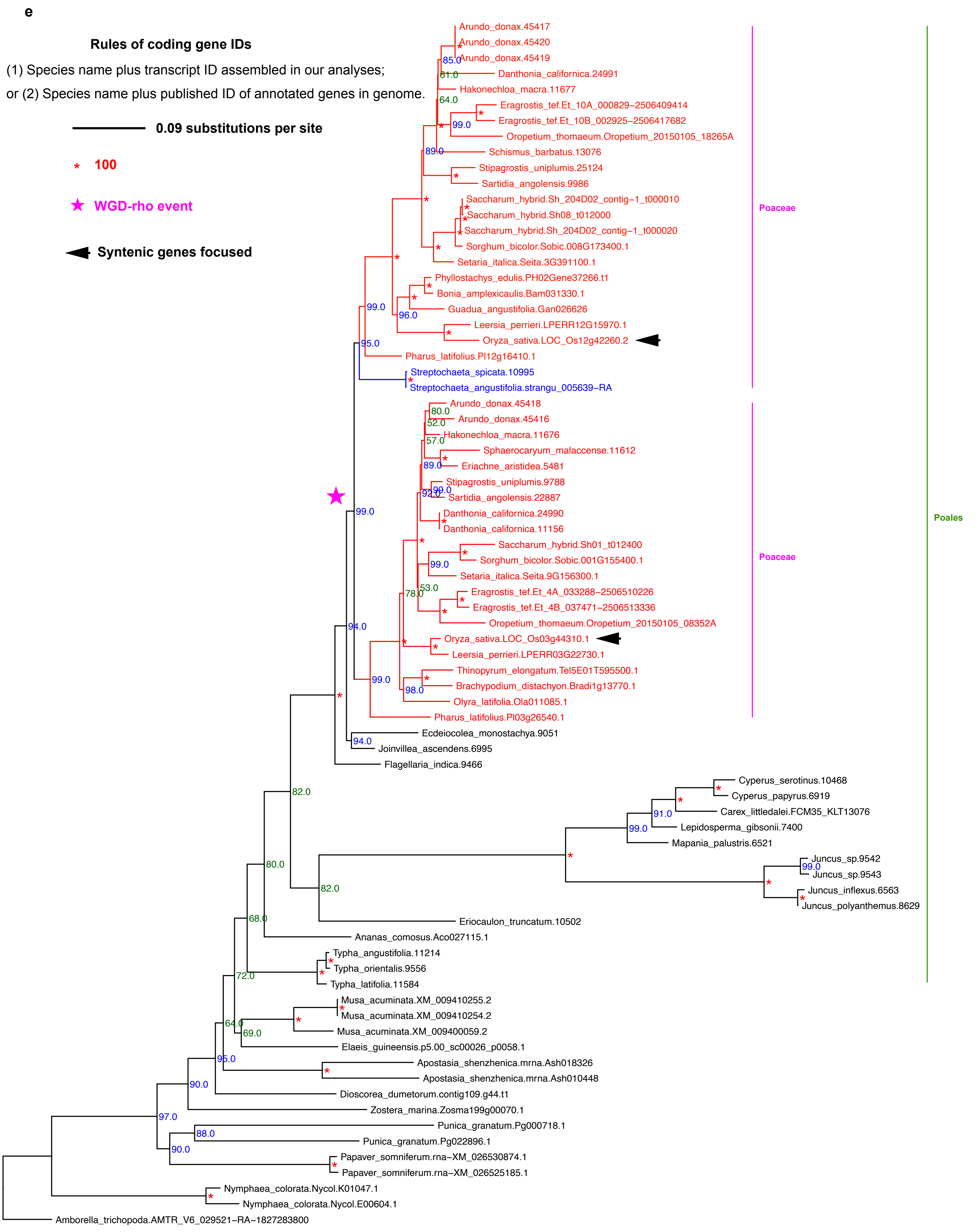

Supplementary Figure 26 Genome synteny summary of GDs at Poaceae in different retention types

(**a**) Summary of GDs shared by paralogs in each species mapped at Poaceae in different retention types. Left phylogenetic tree illustrates the Poaceae phylogeny in Supplementary Fig. 3. Red and blue branches represent the MRCA of Pharoideae and the core Poaceae (referred to as A), and the earliest-branching subfamily (referred to as B), respectively. (AB)(AB) means a pattern of retention of two copies from the A and B lineages. (AB)A means a type of the retention of two copies from the A lineage and retention of only one copy from the B lineage. (AB)B means a type of the retention of two copies from the B lineage and retention of only one copy from the A lineage. Numbers to the right of the species names show numbers of GDs contributed by the paralogs from each species in different retention types. The number in round parentheses represents the number of GDs corresponded to syntenic genes. Numbers of GDs in (AB)(AB), (AB)A, (AB)B, and sum of the above 3 types are shown in green, orange, black, and purple colors, respectively. The total number of GDs are noted above the root branch, and that of GDs in different retention types are noted below the branch. (**b**) Summary of GDs matched by syntenic genes mapped at Poaceae. Meanings of colors and numbers are same as those in part a. Number in round parentheses in orange and black, respectively, represents the number of GDs in (AB)A and (AB)B type, which corresponded to syntenic genes anchored in a block where some gene pairs shared a GD at Poaceae in (AB)(AB) type. (**c**) Illustration of synteny block shared among grasses supporting rho event. Blue and red rectangles represent protein-coding genes with coding or template strands, respectively. Lines between chromosomes from a species represent syntenic genes, and that from different species represent orthologs. In *Oryza sativa*, different gene pairs with a GD at Poaceae in the (AB)(AB) retention type are shown in red, and genes with a GD at Poaceae in the (AB)A retention type are shown in green. (**d**) Gene tree showing a GD (purple star) at Poaceae in the (AB)(AB) retention type. The GD produces two Poaceae clades, each of which contains A and B branches (A and B lineages respectively in red and blue colors). Numbers at nodes represent the support values of bootstrapping 1000 times, of which the maximum support is indicated by a red asterisk (*). Gene pairs from rice in part c are marked by arrows. Gene ID is coded by species name plus transcript ID assembled in our analyses or species name plus published ID of annotated genes in genome. (**e**) Gene tree showing a GD (purple star) at Poaceae in the (AB)A retention type. The number, colors and ID code are same as those in part d. Source data are provided as a Source Data file.

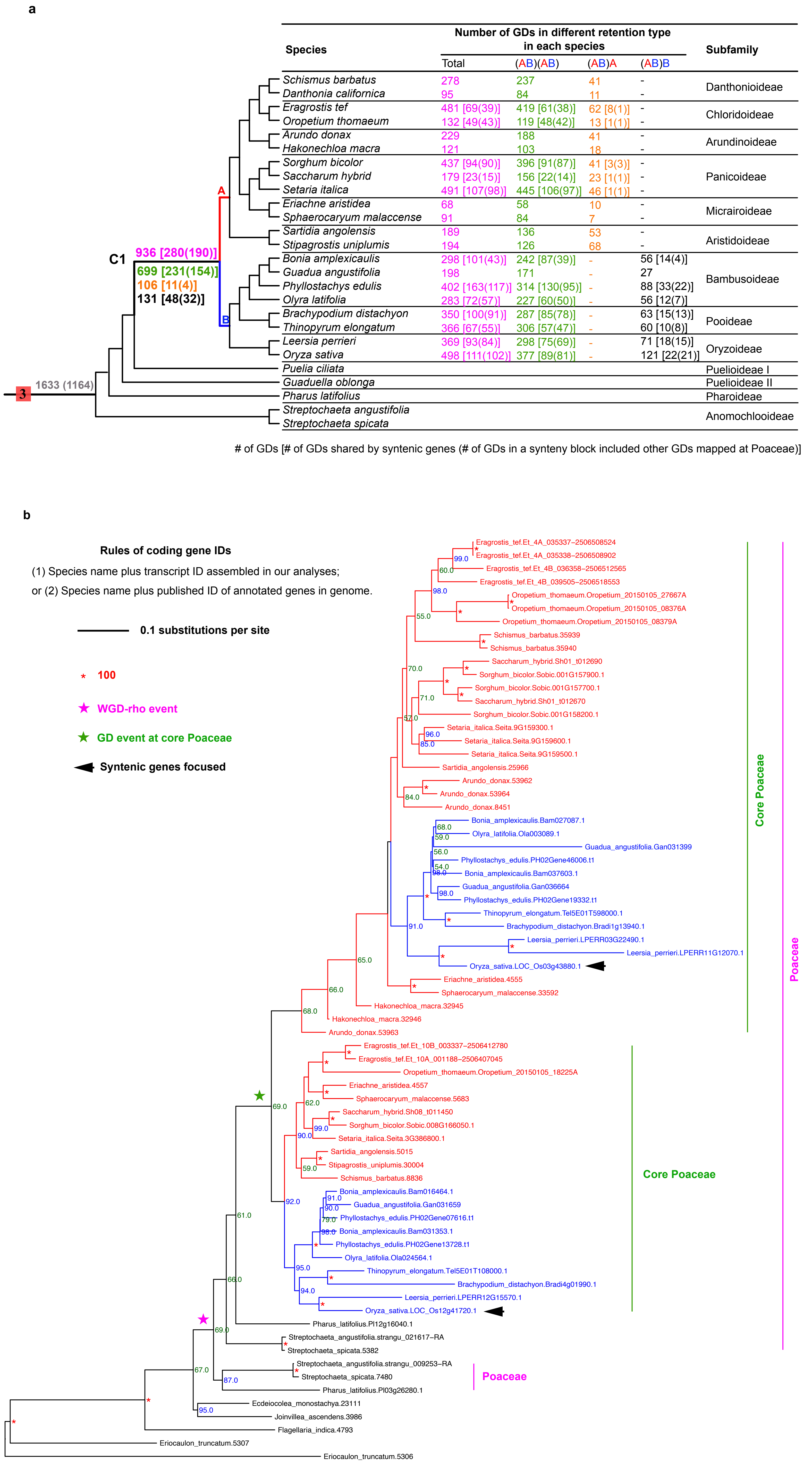

Supplementary Figure 27 Genome synteny summary of GDs at the core Poaceae in different retention type. (**a**) Number of GDs at the core Poaceae in different retention types in each species. Left phylogenetic tree represents a part of the species-tree in Supplementary Fig. 3. Red and blue colors of branches represent the last common ancestor of PACMAD (referred to as A), and BOP (referred to as B), respectively. As shown below, number within square bracket represents the number of GDs shared by syntenic genes, while number within parenthesis represents the number of GDs shared by syntenic genes anchored in a block where other GDs mapped at Poaceae. The total number of GDs in each retention type is showed by respective colors on the core Poaceae in the left tree. Number on the Poaceae ancestor is same as that in Supplementary Fig. 26a. (**b**) Gene tree showing a GD at the core Poaceae (green star at node). The branch colors are same as those in part a. Meanings of the coding of Gene ID and numbers behind the nodes in gene tree are same as those in Supplementary Fig. 26d.

a

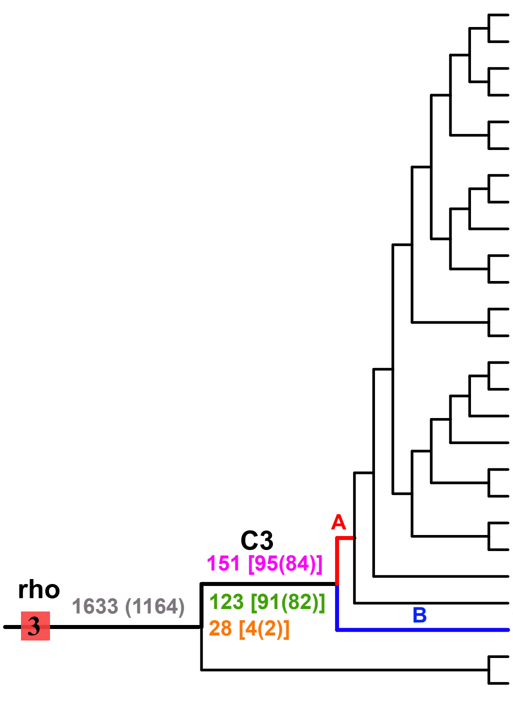

Species	Number of GDs in different retention type in each species: Total	(AB)(AB)	(AB)A	(AB)B	Subfamily
Schismus barbatus	59	54	5	-	Danthonioideae
Danthonia californica	30	28	2	-	
Eragrostis tef	94 [12(9)]	85 [12(9)]	9	-	Chloridoideae
Oropetium thomaeum	37 [25(24)]	35 [24(23)]	2 [1(1)]	-	
Arundo donax	52	44	8	-	Arundinoideae
Hakonechloa macra	31	27	4	-	
Sorghum bicolor	90 [54(52)]	83 [53(51)]	7 [1(1)]	-	Panicoideae
Saccharum hybrid	30 [12(9)]	25 [12(9)]	5	-	
Setaria italica	94 [60(51)]	89 [59(50)]	5 [1(1)]	-	
Eriachne aristidea	19	18	1	-	Micrairoideae
Sphaerocaryum malaccense	24	23	1	-	
Sartidia angolensis	42	37	5	-	Aristidoideae
Stipagrostis uniplumis	34	29	5	-	
Bonia amplexicaulis	90 [34(25)]	84 [33(25)]	6 [1(-)]	-	Bambusoideae
Guadua angustifolia	60	54	6	-	
Phyllostachys edulis	100 [49(47)]	94 [47(46)]	6 [2(1)]	-	
Olyra latifolia	82 [48(38)]	73 [47(37)]	9 [1(1)]	-	
Brachypodium distachyon	91 [53(50)]	82 [52(49)]	9 [1(1)]	-	Pooideae
Thinopyrum elongatum	73 [36(32)]	67 [35(31)]	6 [1(1)]	-	
Leersia perrieri	77 [52(49)]	71 [51(48)]	6 [1(1)]	-	Oryzoideae
Oryza sativa	85 [58(56)]	80 [56(54)]	5 [2(2)]	-	
Puelia ciliata	20	5	15	-	Puelioideae I
Guaduella oblonga	32	4	28	-	Puelioideae II
Pharus latifolius	123 [76(74)]	123 [76(74)]	-	-	Pharoideae
Streptochaeta angustifolia					Anomochlooideae
Streptochaeta spicata					

# of GDs [# of GDs matched by syntenic genes (# of GDs in a synteny block included other GDs mapped at Poaceae)]

b

Rules of coding gene IDs

(1) Species name plus transcript ID assembled in our analyses;

or (2) Species name plus published ID of annotated genes in genome.

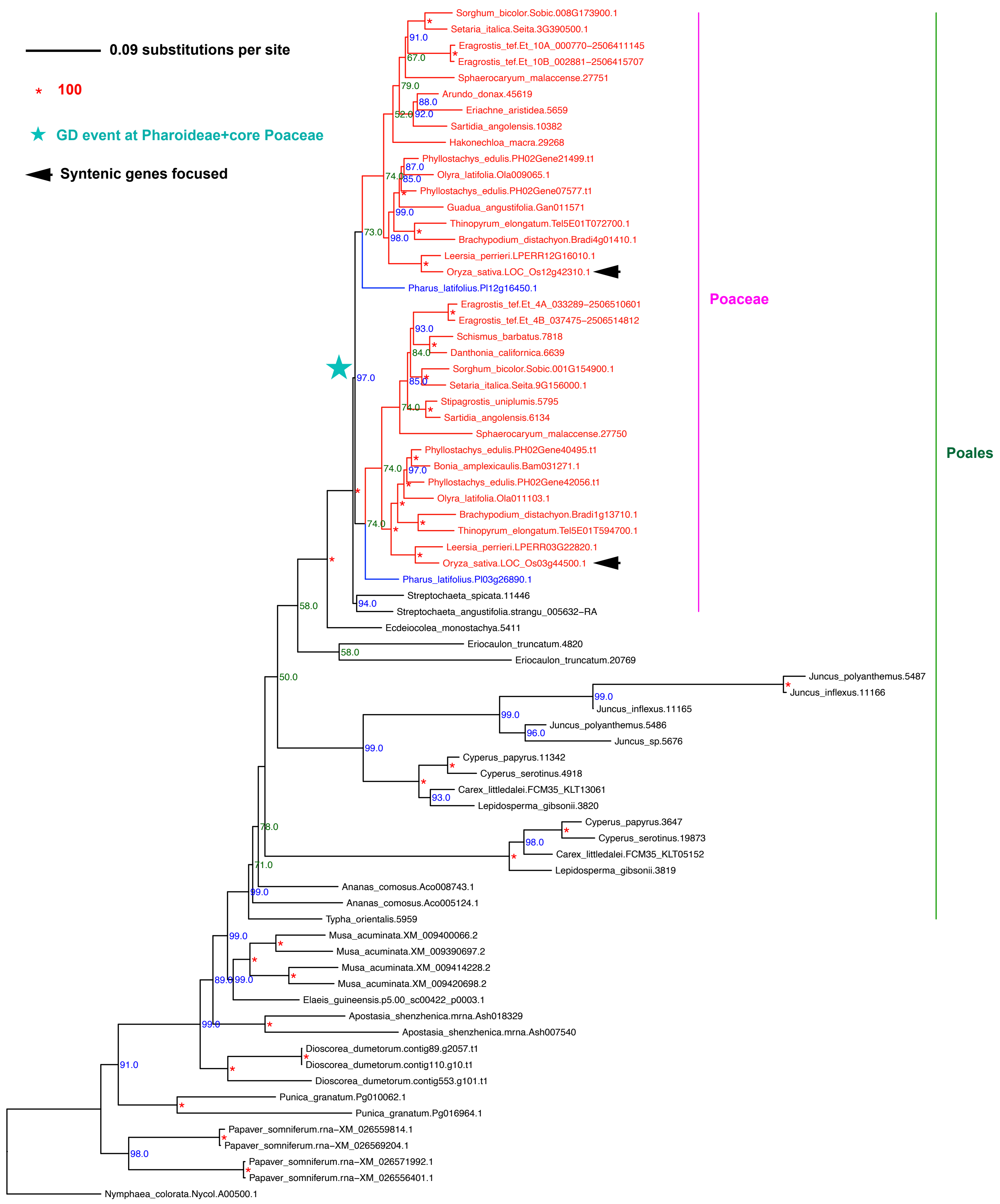

Figure S28 Genome synteny summary of GDs at the last common ancestor of Pharoideae and the core Poaceae in different retention type

(**a**) Summary of GDs at the MRCA of Pharoideae and the core Poaceae in different retention type in each species. Left phylogenetic tree illustrates the Poaceae phylogeny in Supplementary Fig. 3. Red and blue branches represent the MRCA of Puelioideae II and the core Poaceae (referred to as A), and the Pharoideae subfamily (referred to as B), respectively. Numbers to the right of the species names represent the numbers of GDs mapped at the MRCA of Pharoideae and the core Poaceae in different retention type shared by paralogs in respective species. The number in square bracket represents the number of GDs corresponded to syntenic genes; the number in round parentheses represents the number of GDs matched syntenic genes anchored in the block where some syntenic gene pairs are mapped at Poaceae. The total number of GDs in each retention type is showed by respective colors on the MRCA of these lineages, with the C3 representing the GD cluster in Fig. 2. Number on the Poaceae ancestor is same as that in Supplementary Fig. 26a. (**b**) Gene tree showing a GD at ancestor of Pharoideae and the core Poaceae (green star at node). The branch colors are same as those in part A. Meanings of the coding of Gene ID and numbers behind the nodes in gene tree are same as those in Supplementary Fig. 26d.

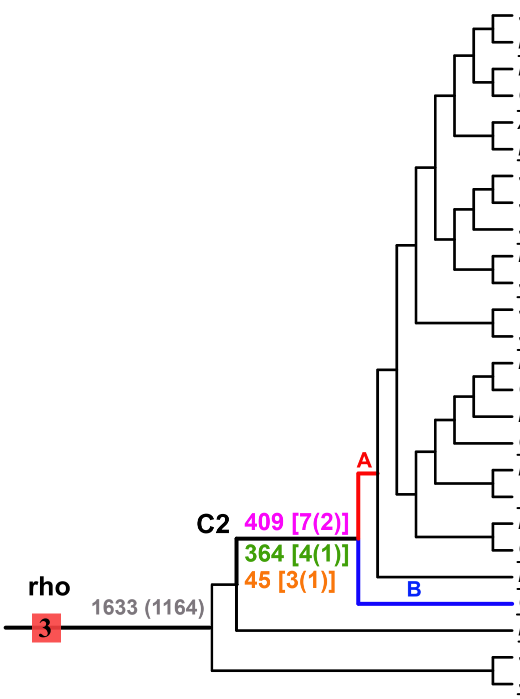

Species	Number of GDs in different retention type in each species: Total	(AB)(AB)	(AB)A	(AB)B	Subfamily
Schismus barbatus	25	14	11	-	Danthonioideae
Danthonia californica	3	1	2	-	
Eragrostis tef	40 [2(-)]	23 [1(-)]	17 [1(-)]	-	Chloridoideae
Oropetium thomaeum	7	4	3	-	
Arundo donax	43	31	12	-	Arundinoideae
Hakonechloa macra	13	8	5	-	
Sorghum bicolor	12	8	4	-	Panicoideae
Saccharum hybrid	5	3	2	-	
Setaria italica	23	14	9	-	
Eriachne aristidea	5	4	1	-	Micrairoideae
Sphaerocaryum malaccense	3	2	1	-	
Sartidia angolensis	15	7	8	-	Aristidoideae
Stipagrostis uniplumis	22	14	8	-	
Bonia amplexicaulis	19 [3(-)]	9 [2(-)]	10 [1(-)]	-	Bambusoideae
Guadua angustifolia	12	6	6	-	
Phyllostachys edulis	19 [2(1)]	10 [1(-)]	9 [1(1)]	-	
Olyra latifolia	12 [1(1)]	7	5 [1(1)]	-	
Brachypodium distachyon	28	13	15	-	Pooideae
Thinopyrum elongatum	15	9	6	-	
Leersia perrieri	12	5	7	-	Oryzoideae
Oryza sativa	28 [1(1)]	14 [1(1)]	14	-	
Puelia ciliata	377	332	45	-	Puelioideae I
Guaduella oblonga	364	364	-	-	Puelioideae II
Pharus latifolius					Pharoideae
Streptochaeta angustifolia					Anomochlooideae
Streptochaeta spicata					

# of GDs [# of GDs matched by syntenic genes (# of GDs in a synteny block included other GDs mapped at Poaceae)]

Supplementary Figure 29 Genome synteny summary of GDs at the last common ancestor of Puelioideae -II and the core Poaceae in different retention type

Left phylogenetic tree represents the Poaceae phylogeny in Supplementary Fig. 3. Red and blue branches, respectively, represent the MRCA of Puelioideae-I and the core Poaceae (referred to as A) and the Puelioideae -II subfamily (referred to as B). Numbers to the right of the species names represent the numbers of GDs mapped at the MRCA of Puelioideae-II and the core Poaceae in different retention type shared by paralogs in respective species. The number in square bracket represents the number of GDs corresponded to syntenic genes; the number in round parentheses represents the number of GDs matched syntenic genes anchored in the block where some syntenic gene pairs are mapped at Poaceae. The total number of GDs in each retention type is shown by respective colors on the MRCA of these lineages. C2 is as Fig. 2a. Number on the Poaceae ancestor is same as that in Supplementary Fig. 26a.

a 190 GDs at the core Poaceae match syntenic genes that are anchored in the block where some GDs are mapped at Poaceae (Supplementary Fig. 27). The main topologies in the 190 GDs are summarized below.

Main Topologies	Percentage of GDs		Number of GDs corresponding to focal gene topologies
Core Poaceae<br>Core Poaceae<br>Early-diverged subfamilies	77.4%<br>(147 out of 190)	**a**	17 GDs in (((Core Poaceae,Core Poaceae),Pharoideae),Anomochlooideae)<br>15 GDs in ((Core Poaceae,Core Poaceae),Pharoideae+Anomochlooideae)<br>7 GDs in (((Core Poaceae,Core Poaceae),Anomochlooideae),Pharoideae)
		**b**	13 GDs in ((Core Poaceae,Core Poaceae),(Anomochlooideae,Anomochlooideae))<br>4 GDs in (((Core Poaceae,Core Poaceae),Anomochlooideae),Anomochlooideae)
		**c**	7 GDs in ((((Core Poaceae, Core Poaceae),Pharoideae),Anomochlooideae),Anomochlooideae)<br>7 GDs in (((Core Poaceae,Core Poaceae),Pharoideae),(Anomochlooideae,Anomochlooideae))<br>5 GDs in (((Core Poaceae,Core Poaceae),Pharoideae+Anomochlooideae),Anomochlooideae)
		**d**	4 GDs in (((Core Poaceae,Core Poaceae),(Pharoideae,Pharoideae),Anomochlooideae)<br>3 GDs in ((((Core Poaceae,Core Poaceae),Anomochlooideae),Pharoideae),Pharoideae)<br>2 GDs in (((Core Poaceae,Core Poaceae),Anomochlooideae),(Pharoideae,Pharoideae))<br>4 GDs in (((Core Poaceae,Core Poaceae),Pharoideae),Pharoideae+Anomochlooideae)
		**e**	9 GDs in ((Core Poaceae,Core Poaceae),Anomochlooideae)<br>5 GDs in ((Core Poaceae,Core Poaceae),Pharoideae)
Core Poaceae<br>Core Poaceae<br>Early-diverged subfamilies +other Poales	6.3%<br>(12 out of 190)		
Core Poaceae<br>Core Poaceae<br>non-Poales	1.1%<br>(2 out of 190)		

a, the major gene topologies matched the loss of one gene from Pharoideae and Anomochlooideae. **b,** the major gene topologies matched the loss of Pharoideae genes. **c,** the major gene topologies matched the loss of one gene of Pharoideae. **d,** the major gene topologies matched the loss of one gene of Anomochlooideae. **e,** the major gene topologies matched the retention of one gene of Pharoideae or Anomochlooideae and the loss of others.

b 84 GDs at the MRCA of Pharoideae and the Core Poaceae (MPCP) match syntenic genes that are anchored in the block where some GDs are mapped at Poaceae (Supplementary Fig. 28). The main topologies in the 84 GDs are summarized below.

Main Topologies	Percentage of GDs	Number of GDs corresponding to different gene topologies
MPCP<br>MPCP<br>Anomochlooideae	82.1%<br>(69 out of 84)	35 GDs in ((MPCP,MPCP),Anomochlooideae)<br>24 GDs in ((MPCP,MPCP),(Anomochlooideae,Anomochlooideae))<br>10 GDs in (((MPCP,MPCP),Anomochlooideae),Anomochlooideae)
MPCP<br>MPCP<br>Anomochlooideae+other Poales	6.0%<br>(5 out of 84)	
MPCP<br>MPCP<br>other Poales	2.4%<br>(2 out of 84)	

c

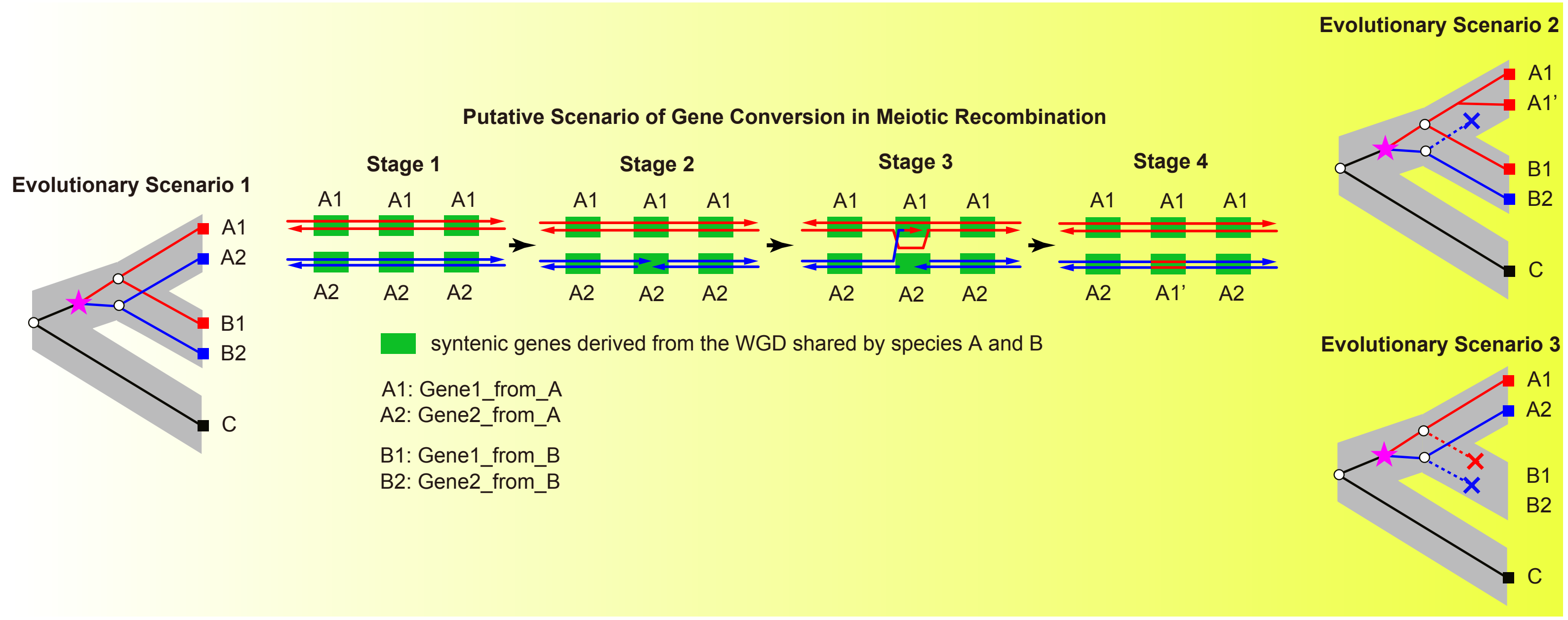

Supplementary Figure 30 A model of gene conversion for gradual placements of the rho-derived gene paralogs in Poaceae (**a**) Patterns of GDs matched by the rho-derived syntenic genes mapped at the core Poaceae with different outgroups. (**b**) Patterns of GDs matched by the rho-derived syntenic genes mapped at the MRCA of Pharoideae and the core Poaceae with different groups. (**c**) A model of gene conversion. In the Evolutionary Scenario 1, gray thick lines illustrate the phylogeny among species A, B, and C; other lines show the gene tree of orthogroup that includes two paralogs from A (A1 and A2; in red lines), two paralogs from B (B1 and B2; in blue lines) and the gene from C. Purple star represents a GD shared by A and B. Open circles at nodes represent speciation events. The illustration to the right of the gene tree shows a possible scenario in meiotic recombination for syntenic genes derived from the WGD event shared by species A and B. Red and blue lines represent the WGD-derived homeologous chromosomes in species A, with arrows showing the transcriptional direction. Three pairs of syntenic genes are represented by green boxes and the gene IDs are the same as IDs in the gene tree. The 4-stage scenario is illustrated according to the gene conversion model in Duret and Galtier[15]. Briefly, recombination is initiated by the formation of a double-strand-break (Stage 1), followed by 5' to 3' resection (Stage 2). One DNA tail invades the homologous DNA duplex and forms a displacement (D)-loop for DNA synthesis (Stage 3). Mismatch repair in heteroduplex DNA and new DNA synthesis lead to A1' being identical or similar to A1 (Stage 4). After gene conversion, A2 was lost and A1' was produced. Therefore, gene tree of the new orthogroup suggests a close relationship between A1 and A1' as shown in the Evolutionary Scenario 2. In addition, two gene trees in part (a) show the gene loss in early-diverging subfamilies and might support another model (Evolutionary Scenario 3) that the losses of genes from early-diverging grasses lead to the GD placement at the core Poaceae.

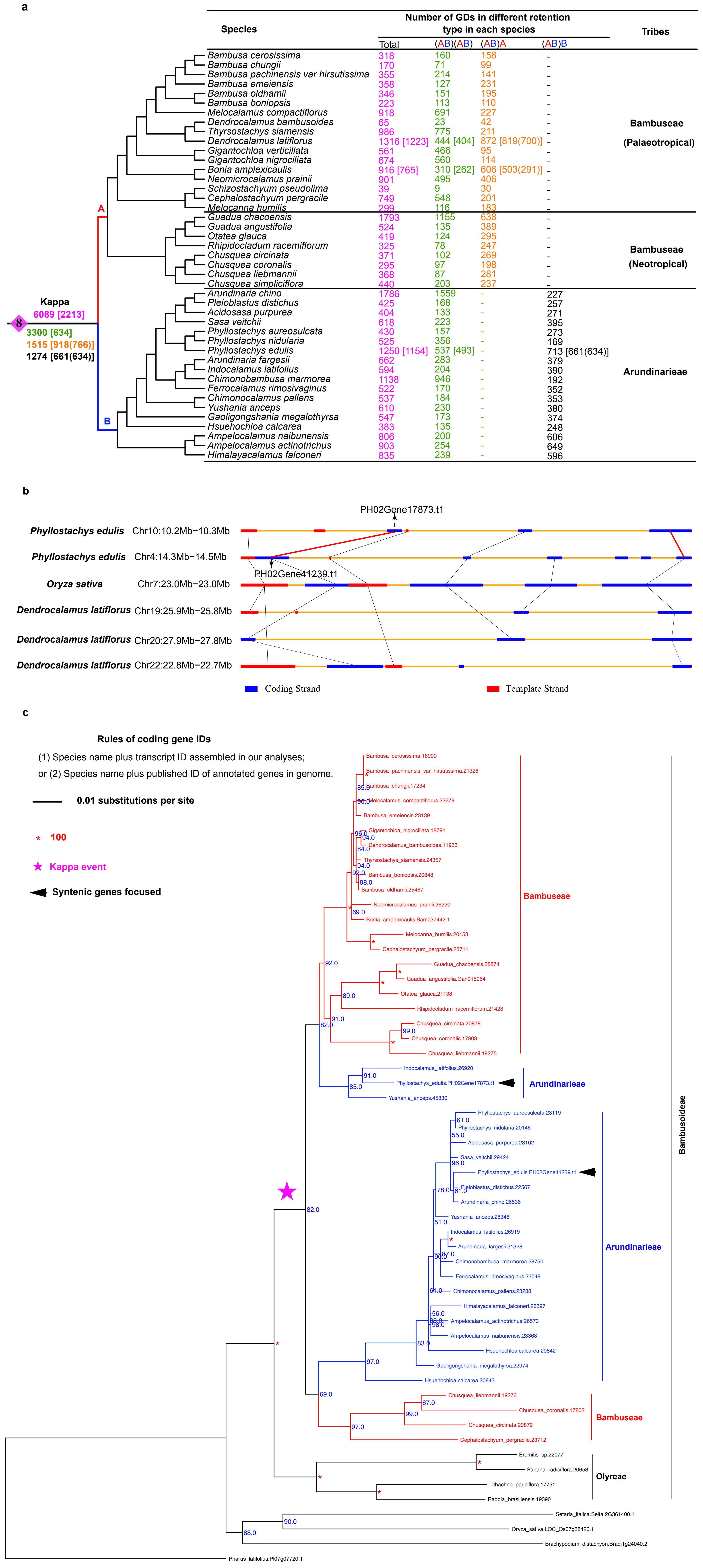

Supplementary Figure 31 Genome synteny summary of GDs at woody bamboo ancestor in different retention type
(**a**) Number of GDs mapped at woody bamboo MRCA in different retention type in each species. Left phylogenetic tree illustrates the woody bamboo phylogeny in Supplementary Fig. 5. Red and blue branches, respectively, represent the Bambuseae tribe (referred to as A) and the Arundinarieae tribe (referred to as B). The number in square brackets represents the number of GDs matched by syntenic genes. Numbers of GDs in the (AB)(AB), (AB)A, (AB)B, and sum of the above 3 types are showed in green, orange, black, and purple colors, respectively. The total number of GDs are showed above the root branch, and that of GDs in different retention types are below the branch. The number in round parentheses represents the number of GDs of the (AB)A or (AB)B type anchored in the syntenic block that also contains some gene pairs corresponding to the GDs at woody bamboo ancestor in the (AB)(AB) type. (**b**) A sample of the synteny block supporting the kappa event. Meanings of rectangles and lines are same as those in Supplementary Fig. 26c. The red line shows two *Phyllostachys edulis* syntenic paralogs that diverged at the woody bamboo ancestor in the respective phylogeny. (**c**) Gene tree showing the duplication of a pair of syntenic genes of part b mapped at woody bamboo ancestor (purple star at node). Meanings of the coding of Gene ID and numbers behind the nodes in gene tree are same as those in Supplementary Fig. 26d. Gene IDs with arrows are focal syntenic gene pairs as shown in part b. Source data are provided as a Source Data file.

a

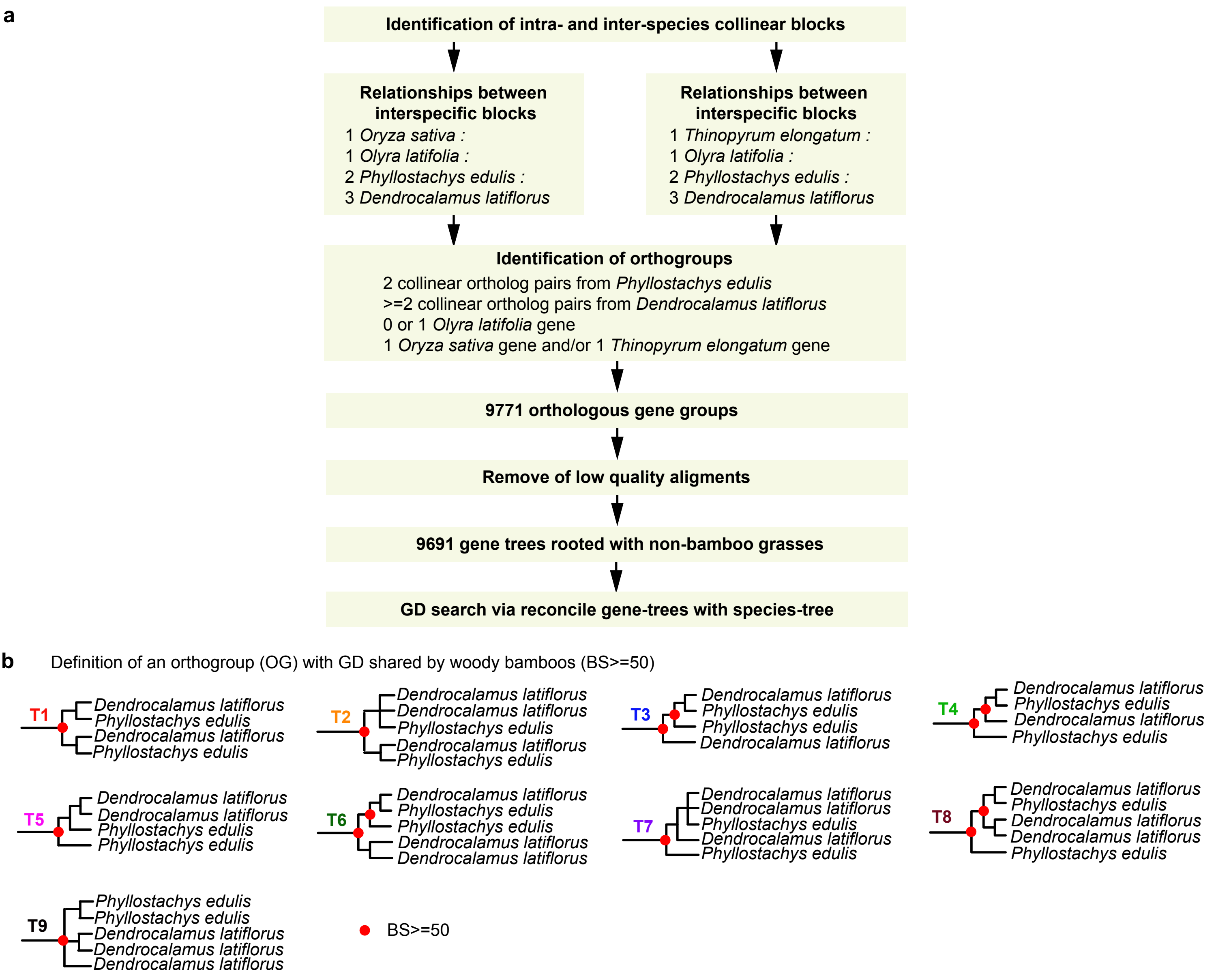

c

Summary of OGs with GDs shared by woody bamboos

Type	# of OGs BS>=50
T1	724 [724]
T2	418 [418]
T3	466 [466 (464)]
T4	417 [417 (414)]
T5	104 [104 (103)]
T6	94 [94 (93)]
T7	134 [134 (133)]
T8	43 [43 (43)]
T9	30 [26 (25)]
Total	2430 [2426 (2417)]

[#] # of OGs with GDs matched by syntenic genes
(#) # of OGs with GDs anchored in the syntenic blocks where other GDs are placed into T1 and/or T2 types

d

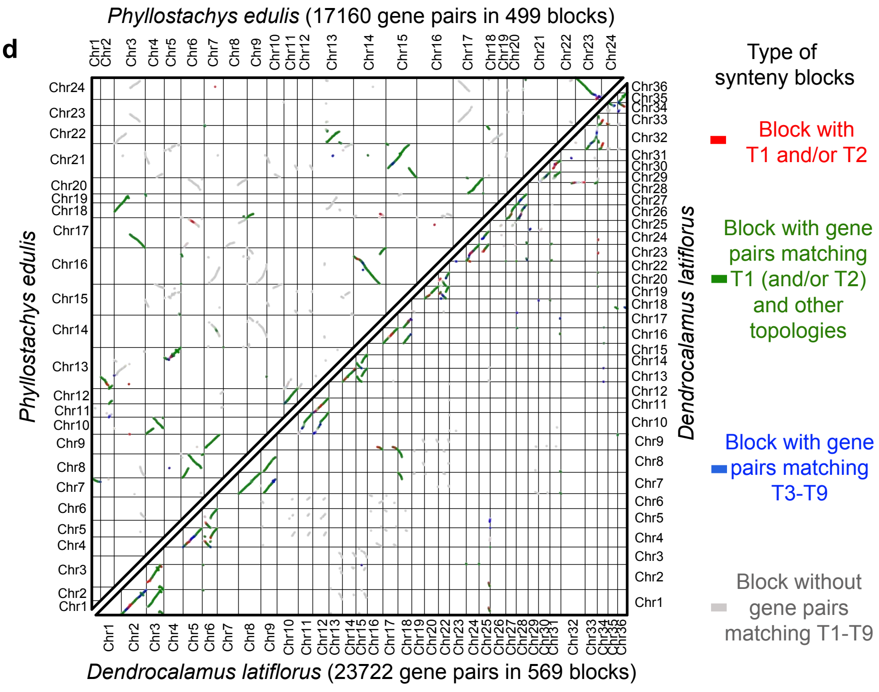

e

Comparison of OGs matched by the same syntenic genes in the phylogenomic analyses here and that in Supplementary Fig. 5

Type		# OGs with GDs (BS>=50)			
	in (C)	overlapped with GDs mapped at the woody bamboo MRCA in Supplementary Fig. 5			
		(AB)(AB)	(AB)A	(AB)B	total
T3	466	19 [19]	26 [25(23)]	27 [27(25)]	72 [71(67)]
T4	417	20 [20]	17 [17(15)]	28 [28(27)]	63 [63(60)]
T5	104	0	0	1 [1(1)]	1 [1(1)]
T6	94	5 [5]	11 [11(11)]	10 [10(10)]	26 [26(26)]
T7	134	10 [10]	8 [7(7)]	9 [9(7)]	27 [26(24)]
T8	43	2 [1]	6 [5(5)]	5 [5(4)]	13 [11(10)]
T9	30	1 [1]	2 [2(2)]	1 [1(1)]	4 [4(4)]
Total	1288				206 [202(192)]

[#] # OGs with GDs matched by syntenic genes
(#) # OGs with GDs anchored in the syntenic blocks that also contain gene duplicates mapped at the woody bamboo MRCA in (AB)(AB) type

f **g**

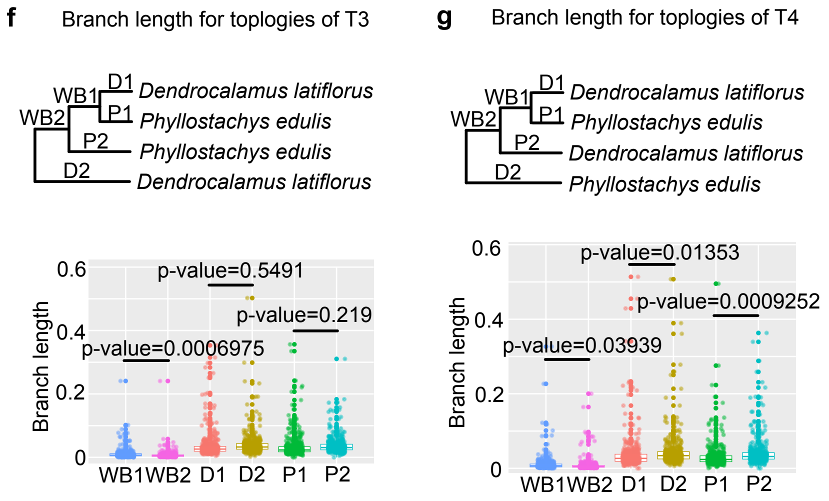

Supplementary Figure 32 Analyses of GDs matched by syntenic genes from woody bamboos

(**a**) Pipeline to analyze GDs. To support the kappa event, genomes from 3 bamboos [including *Olyra latifolia* (Olyreae), *Phyllostachys edulis* (Arundinarieae), and *Dendrocalamus latiflorus* (Bambuseae)] and 2 outgroups (*Oryza sativa* and *Thinopyrum elongatum*) are compared by using MCScan to identify collinear blocks with 1 outgroup versus 1 *O. latifolia* versus 2 *P. edulis* versus 3 *D. latiflorus*. In a block, when tandem duplicates in single species correspond to >=2 orthogroups, which should be placed into 1 group, the tandem gene with the longest alignments and the highest sequence identities against other orthologs is selected (see methods). 9,771 orthogroups are detected to include 2 syntenic genes from *P. edulis*, >=2 syntenic genes from *D. latiflorus*, 0 or 1 *O. latifolia* gene, and 1 *O. sativa* gene and/or 1 *T. elongatum* gene. Excluding 80 orthogroups due to low alignment quality for tree constructing, gene trees of 9,691 orthogroups are reconstructed by IQ-TREE. Gene-trees are rooted with non-bamboo grasses and reconciled to species-tree [((((*P. edulis*, *D. latiflorus*), *O. latifolia*), *T. elongatum*), *O. sativa*)] to detect GDs. (**b**) Definition of orthogroups (OGs) with GDs shared by woody bamboos. We focused on 9 gene topologies (T1 through T9), with the T1 and T2 showing the typical (AB)(AB) retention type of GDs for supporting the kappa event. (**c**) Summary of GDs shared by woody bamboos. The number of GDs matched by syntenic genes is shown in square brackets and that anchored in the syntenic blocks with some other genes of topologies of Type 1 and/or Type2 is in round parentheses. Specifically, many syntenic genes from WBs correspond to 724 GDs of T1, with the gene topology of (*P. edulis*, *D. latiflorus*) (*P. edulis*, *D. latiflorus*), supporting the kappa event detected by the above Analysis-I. Another GD-cluster with 418 GDs (T2) also directly supports kappa, with two *P. edulis* paralogs and three *D. latiflorus* paralogs forming two WB clades in gene trees. (**d**) Illustration of intra-species chromosome collinear blocks with top-left dot-plot showing the *P. edulis* genome and bottom-right dot-plot showing the *D. latiflorus* genome. Red blocks represent the syntenic blocks with the syntenic genes showing the gene topologies of Type1 and/or Type2. Green blocks represent the syntenic blocks with the syntenic genes showing the gene topologies of Type1 (and/or Type2) and other types. Blue blocks represent the syntenic blocks with the syntenic genes in gene topologies of Types 3-9. Black blocks represent the blocks without genes matched Types 1-9. Specifically, in the *P. edulis* genome, 91 syntenic blocks have gene pairs with the T1 and/or T2 topologies and a total of 12,268 syntenic gene pairs, which account for 71.5% of all detected syntenic gene pairs (17,160 pairs in 499 blocks). Similarly, in the *D. latiflorus* genome, 220 syntenic blocks contain gene pairs with T1 and/or T2 topologies and a total of 20,580 gene pairs (accounting for 86.8% of 23,722 gene pairs in a total of 569 blocks). Other OGs with topologies 3-9 account for 53% (1,288) of the 2,430 GDs (Supplementary Fig. 32b, 32c); these contain one clade with both *P. edulis* and *D. latiflorus* genes, consistent with them having a WB ancestral gene. (**e**) Comparison of GDs matched by the same syntenic genes in the phylogenomic analyses here and that in Supplementary Fig. 5 showing that some genes of Types 3-9 can be changed into being Type1/2 via increasing bamboo species. (**f**) Comparison of branch lengths of gene trees with GDs in type-3 showing different evolutionary rates between paralogs retained from Kappa. The center line in box-plot marks the median. (**g**) Comparison of branch lengths of gene trees with GDs in type-4 showing different evolutionary rates between paralogs retained from Kappa. The center line in box-plot marks the median. P-value in f and g was estimated by by t.test (alternative = "two.sided") in R. Source data are provided as a Source Data file.

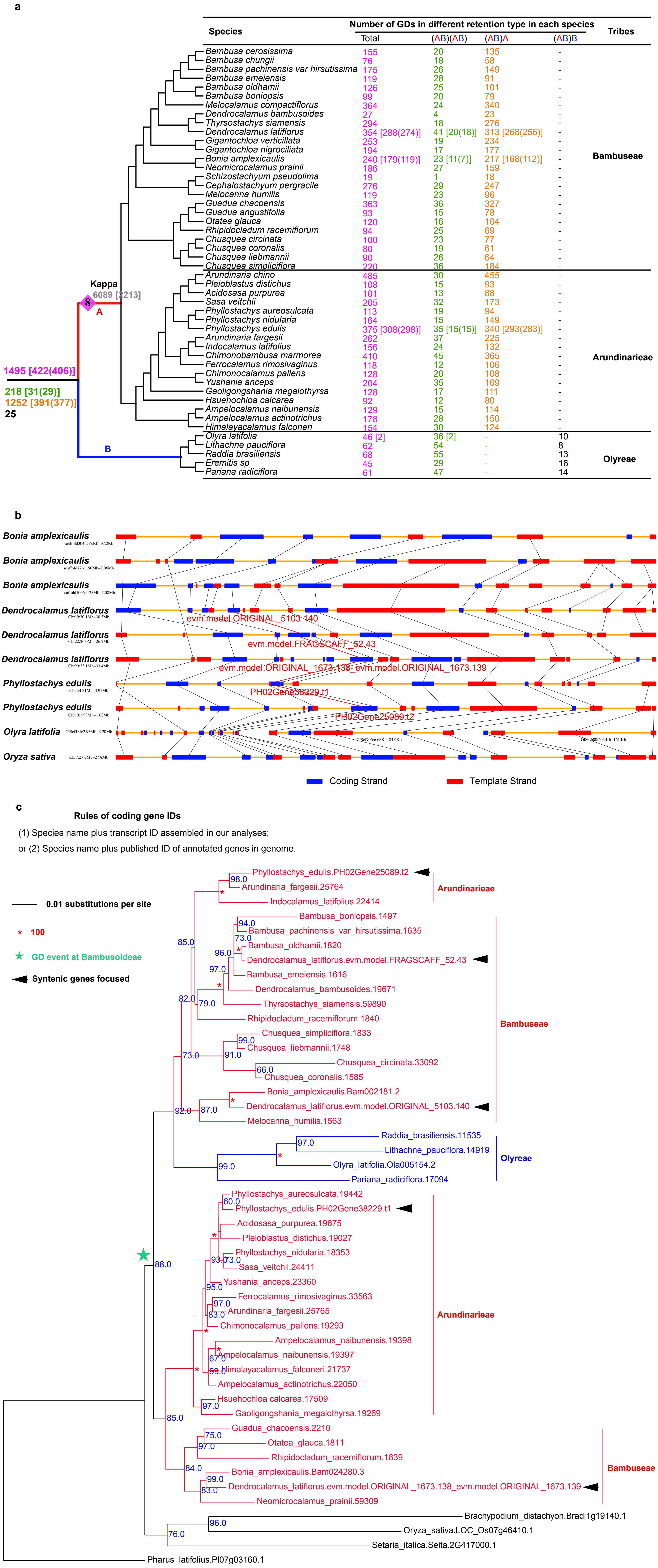

Supplementary Figure 33 Genome synteny summary of GDs at Bambusoideae in different retention type

(**a**) Number of GDs mapped at Bambusoideae in different retention type in each species. Left phylogenetic tree illustrates the Bambusoideae phylogeny in Supplementary Fig. 5. Red and blue branches, respectively, represent the woody bamboos (referred to as A) and the herbaceous bamboos (referred to as B). Meaning of the numbers in square brackets and round parenthesis, in addition to the color of numbers, are same as those in Supplementary Fig. 31a. The total number of GDs in each retention type is shown by respective colors on bamboo ancestor in the phylogeny. Number on the woody bamboo ancestor is same as that in Supplementary Fig. 31a. (**b**) A sample of the synteny block supporting the kappa event. Meanings of rectangles and lines are same as those in Supplementary Fig. 26c. Red lines indicate a bamboo orthogroup with syntenic paralogs from *Phyllostachys edulis* and *Dendrocalamus latiflorus* diverged at Bambusoideae. (**c**) Gene tree showing the duplication of syntenic genes in part b mapped at bamboo ancestor (green star at node). Meanings of the coding of Gene ID and numbers at nodes in gene tree are same as those in Supplementary Fig. 26d. Gene IDs with arrows are focal syntenic gene pairs as shown in part b. Source data are provided as a Source Data file.

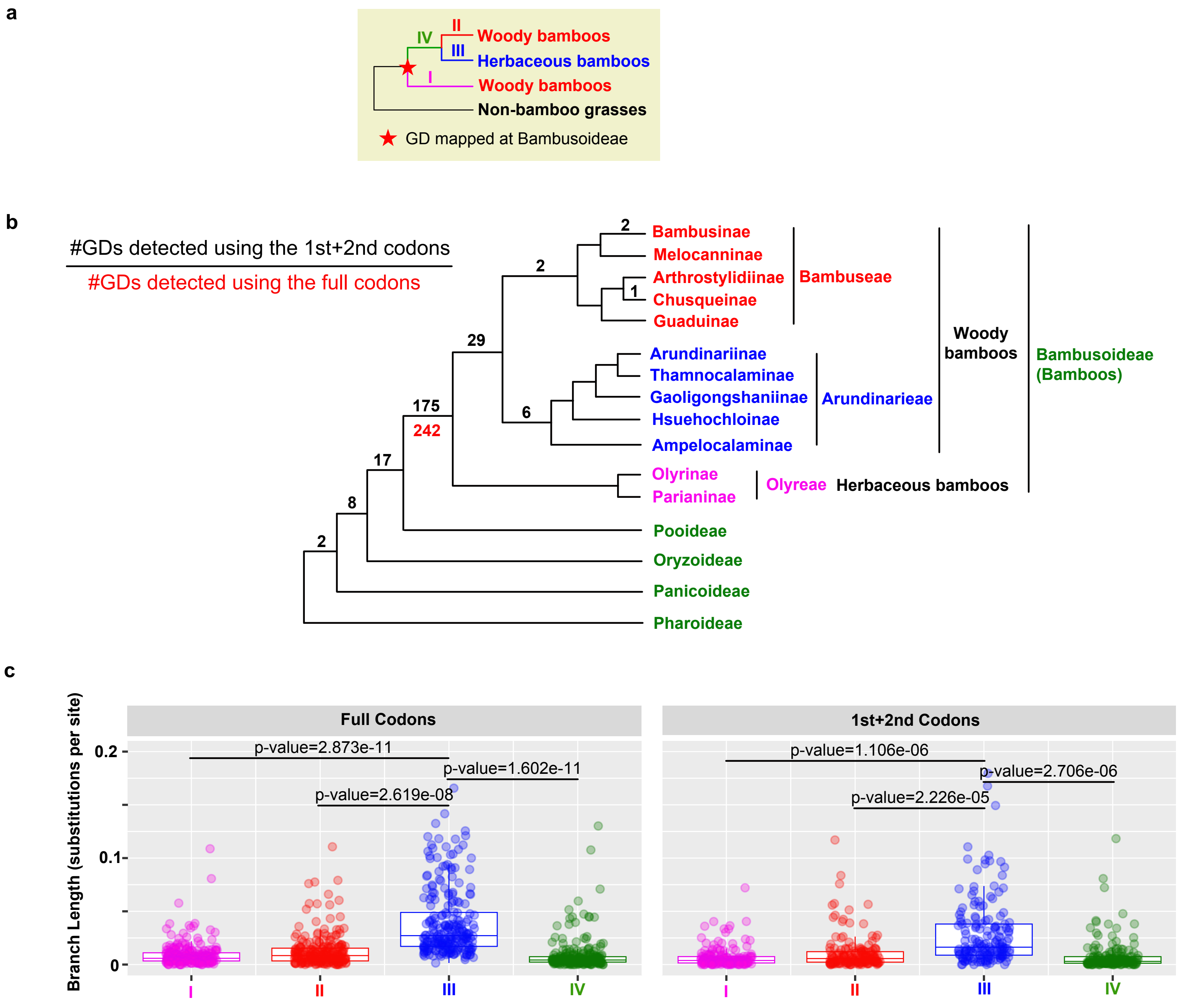

Supplementary Figure 34 Examination of the effect of long branch attraction artifacts on GD detection
(**a**) Illustration of the gene topology with a GD mapped at Bambusoideae in retention type of [(woody bamboo, herbaceous bamboo) woody bamboo] (referred to as (AB)A below). If paralogs from woody bamboos have unusually high substitution rates, the woody bamboo gene with high evolution rate can be placed sister to bamboos due to long branch attraction (LBA) artifacts, resulting into incorrect placement of the GD on root. One of approaches to reduce the LBA artifacts is construction of gene trees using the first and second codon positions. To examine the potential effects of LBA artifacts in detection of GDs mapped at Bambusoideae of the (AB)A type, 242 gene trees with non-bamboo grasses as outgroup are reconstructed by using the 1st+2nd codons and used to detect GDs. (**b**) Comparison of the number of GDs in gene trees using full codons and the 1st+2nd codon positions. Phylogeny is a part of tree in Supplementary Fig. 5, illustrating tribal relationships in Bambusoideae. Red number below branch represents the number of GDs (BS>=50) retrieved from gene trees using full codons. Number on branch represents the number of GDs (BS>=50) retrieved from gene trees using the 1st+2nd codons. The reconciliation shows the greatest number of GDs (BS>=50) mapped at Bambusoideae, albeit with a few GDs placed on woody bamboo ancestor and the ancestors of Bambusoideae and other grasses, in consistent with that from full codons, indicating that the GDs at Bambusoideae using full codons are robust. (**c**) Comparison of branch lengths among two woody bamboo lineages (I, II), the herbaceous bamboo lineage (III), and the Bambusoideae lineage (IV) consisting of II and III in full codons and the 1st+2nd codon positions. Different lineages are shown in different colors. P-value in was estimated by by t.test (alternative ="two.sided") in R. The center line in box-plot marks the median. Source data are provided as a Source Data file.

a, Pipeline to compare bamboo genomes

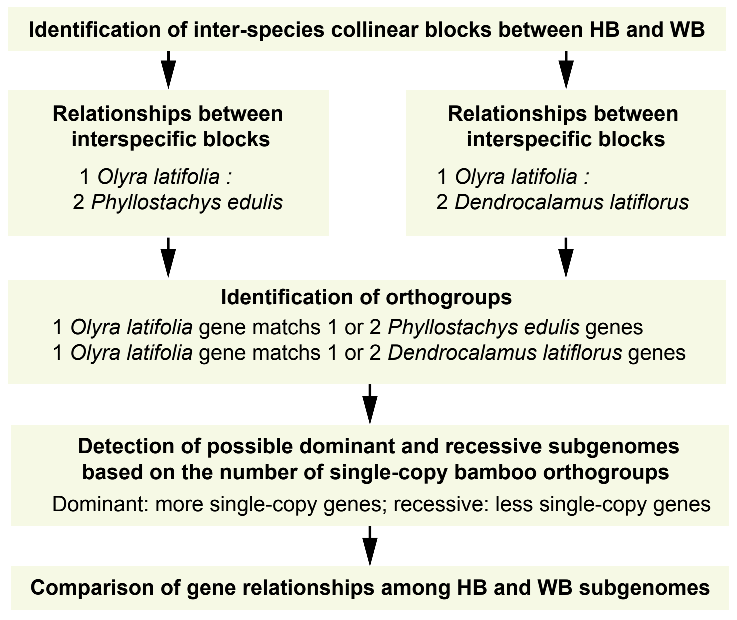

b, An example of chromosomal collinear relationships between HB and WB

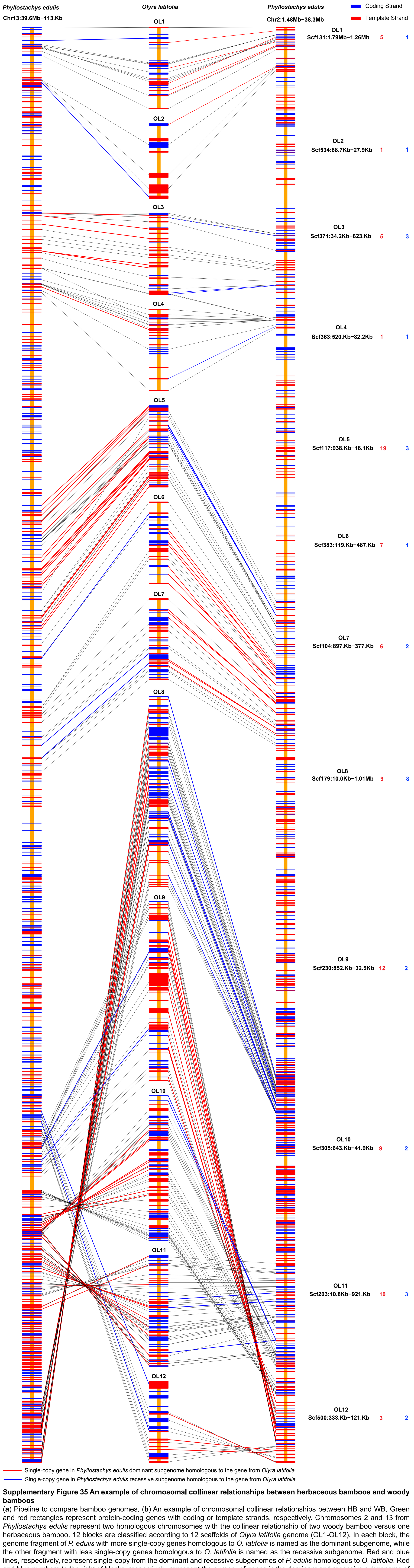

Supplementary Figure 35 An example of chromosomal collinear relationships between herbaceous bamboos and woody bamboos

(**a**) Pipeline to compare bamboo genomes. (**b**) An example of chromosomal collinear relationships between HB and WB. Green and red rectangles represent protein-coding genes with coding or template strands, respectively. Chromosomes 2 and 13 from *Phyllostachys edulis* represent two homologous chromosomes with the collinear relationship of two woody bamboo versus one herbaceous bamboo. 12 blocks are classified according to 12 scaffolds of *Olyra latifolia* genome (OL1-OL12). In each block, the genome fragment of *P. edulis* with more single-copy genes homologous to *O. latifolia* is named as the dominant subgenome, while the other fragment with less single-copy genes homologous to *O. latifolia* is named as the recessive subgenome. Red and blue lines, respectively, represent single-copy from the dominant and recessive subgenomes of *P. edulis* homologous to *O. latifolia*. Red and blue numbers to the right of blocks, respectively, represent the number of genes in the dominant and recessive subgenome of *P. edulis* corresponding to the single-copy genes.

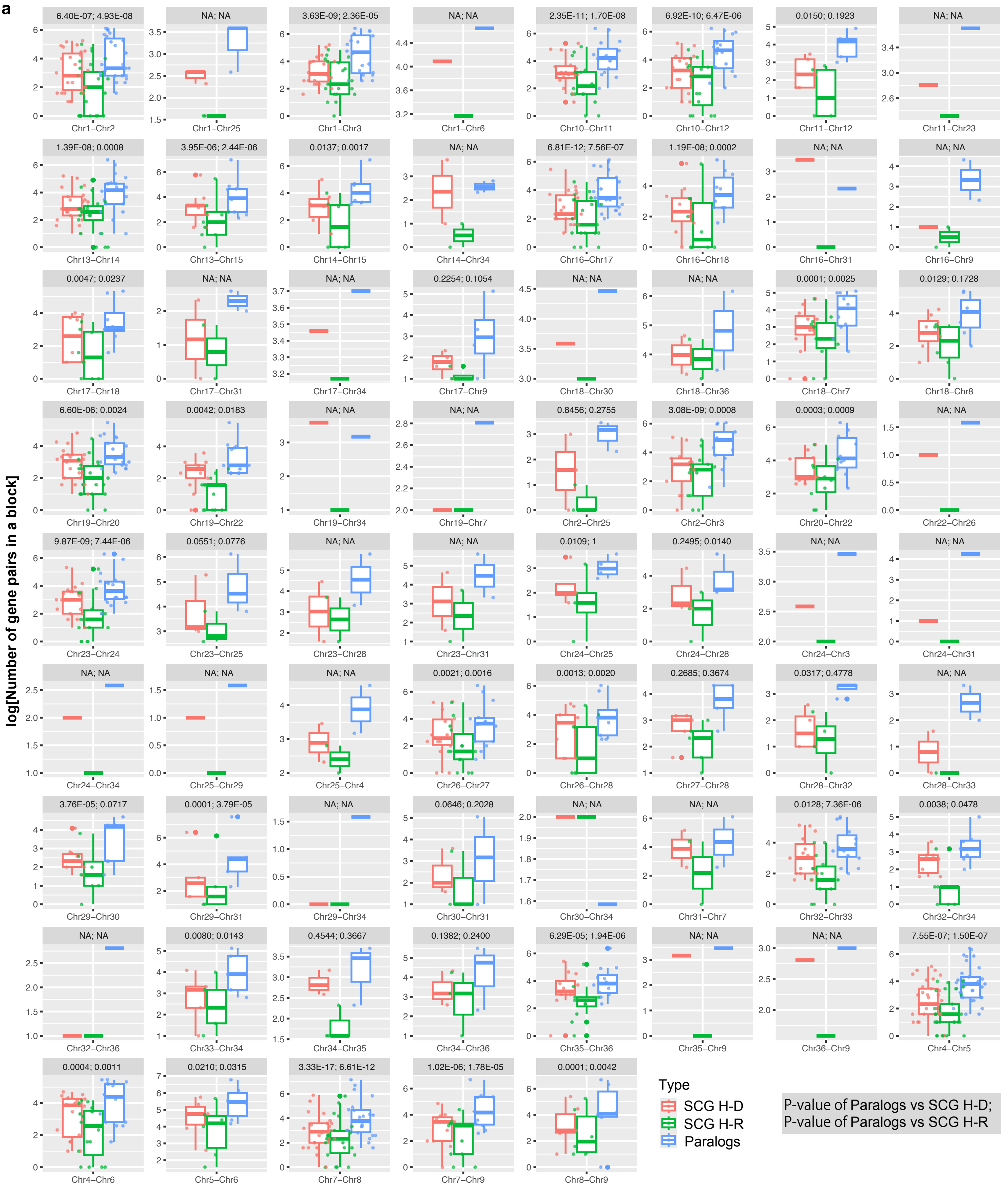

Cluster of *Dendrocalamus latiflorus* genome blocks with a synteny ratio of 1:2 for *Olyra latifolia* versus *Dendrocalamus latiflorus*

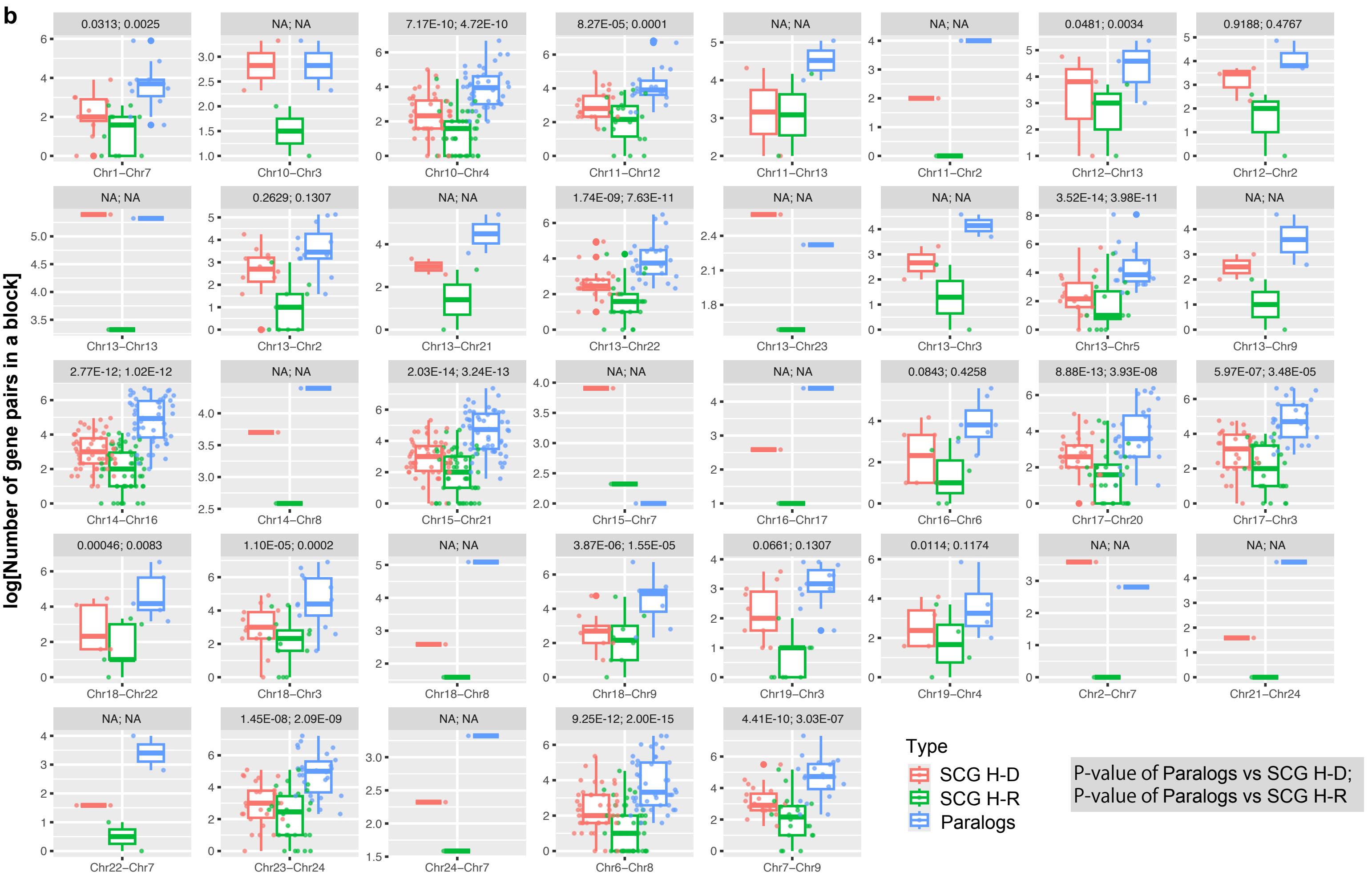

Cluster of *Phyllostachys edulis* genome blocks with a ratio of 1:2 for *Olyra latifolia* versus *Phyllostachys edulis*

Supplementary Figure 36 Genomic landscape of the retention patterns of orthologous genes between interspecific collinear blocks

Box-plots show the genomic landscape of orthologous gene retention patterns between *Dendrocalamus latiflorus* and *Olyra latifolia* genomes (a) and between *Phyllostachys edulis* and *O. latifolia* genomes (b). Different homologous chromosome combinations implicate several genome combination events (including fissions and fusions) in woody bamboos after the kappa event. SCG H-D, single-copy gene between herbaceous bamboo and the dominant subgenome of woody bamboo. SCG H-R, single-copy gene between herbaceous bamboo and the recessive subgenome of woody bamboo. The SCG H-D and SCG H-R implicate gene flows between woody bamboos and herbaceous bamboos. The genome-wide survey of retention patterns of the closest homologs between WBs and *O. latifolia* genomes revealed that the numbers of SCGs on two chromosome regions with the syntenic relationships are often not even, with one of the regions having more SCGs (SCG H-D) than the other region (SCG H-R). In addition, the number of orthogroups with two WB copies (likely one from each parent) and one herbaceous gene is greater than both subsets of SCGs. These results support the proposed hybridization involving an HB-like parent for the polyploidy (kappa) of the WB ancestor. P-value was estimated by aov() in R. Source data are provided as a Source Data file.

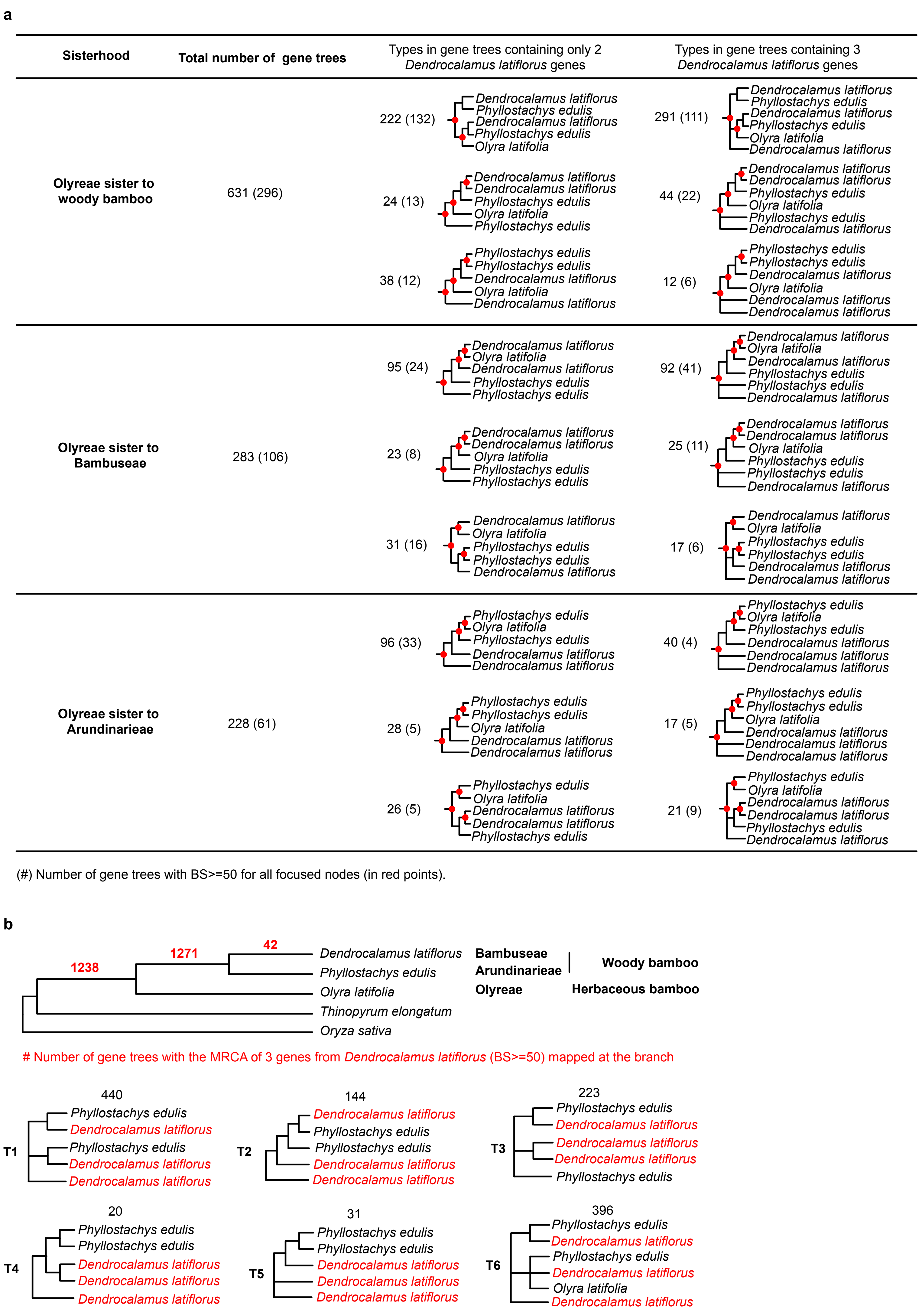

Supplementary Figure 37 Summary of different evolutionary patterns for hybridization between herbaceous bamboos and woody bamboos and the third gene copy of *Dendrocalamus latiflorus*

(**a**) Comparison of different sisterhood relationships of herbaceous bamboos in gene trees. To examine the possibly evolutionary history of hybridization between herbaceous bamboos and woody bamboos, we estimated the number of gene trees with three types of placements of herbaceous bamboos (Olyreae) relative to woody bamboo lineages, including Olyreae sister to (1) woody bamboos, (2) Bambuseae, and (3) Arundinarieae. Specifically, we used the gene trees that contain syntenic genes (as described in Supplementary Fig. 32) with three *Dendrocalamus latiflorus* genes or two *D. latiflorus* genes due to gene loss and/or putatively incomplete genome annotation. For type 1 (631 gene trees), the sisterhood of Olyreae genes with one subclade of woody bamboo genes supports the ancient gene flow between herbaceous bamboos and one parental lineage of the kappa polyploidization event of the woody bamboo ancestor. (**b**) Illustration of all possible origination of the third *D. latiflorus* gene in gene trees with GDs mapped at woody bamboo or Bambusoideae. Number on a branch represents the number of gene trees with the MRCA of 3 genes from D. latiflorus (BS>=50) mapped at the branch. The greatest value (1271) among the surveyed numbers is mapped at the MRCA of woody bamboos and therefore means that the origination of the third subgenome of *D. latiflorus* is putatively from the woody bamboo ancestor after it split from the last common ancestor of bamboos. In particular, we illustrate 6 main gene tree topologies (T1-T6) to discuss the possible origination. T1 (with 440 trees) represents a simplified relationship among two woody bamboo subgenomes and the 3rd *D. latiflorus* gene that possibly originated before the woody bamboo ancestor divergence. The origination is also supported by the relationships in T2, although one *Phyllostachys edulis* gene is not placed as sister to *D. latiflorus* in T2 partly due to rapid sequence divergence when compared with that in T1. Other gene trees (T3 and T4) could be explained by gene conversion involving *D. latiflorus* gene copies, whereas 396 gene trees of T6 are consistent with both the hybridization with herbaceous bamboo and the proposed origin of the third hexopoid subgenome. Gene conversion in *P. edulis* can explain the relationships in T5 where one *D. latiflorus* gene is not placed as sister to *P. edulis*, although rapid sequence divergence of the *D. latiflorus* gene is also a putative reason for the observed topology.

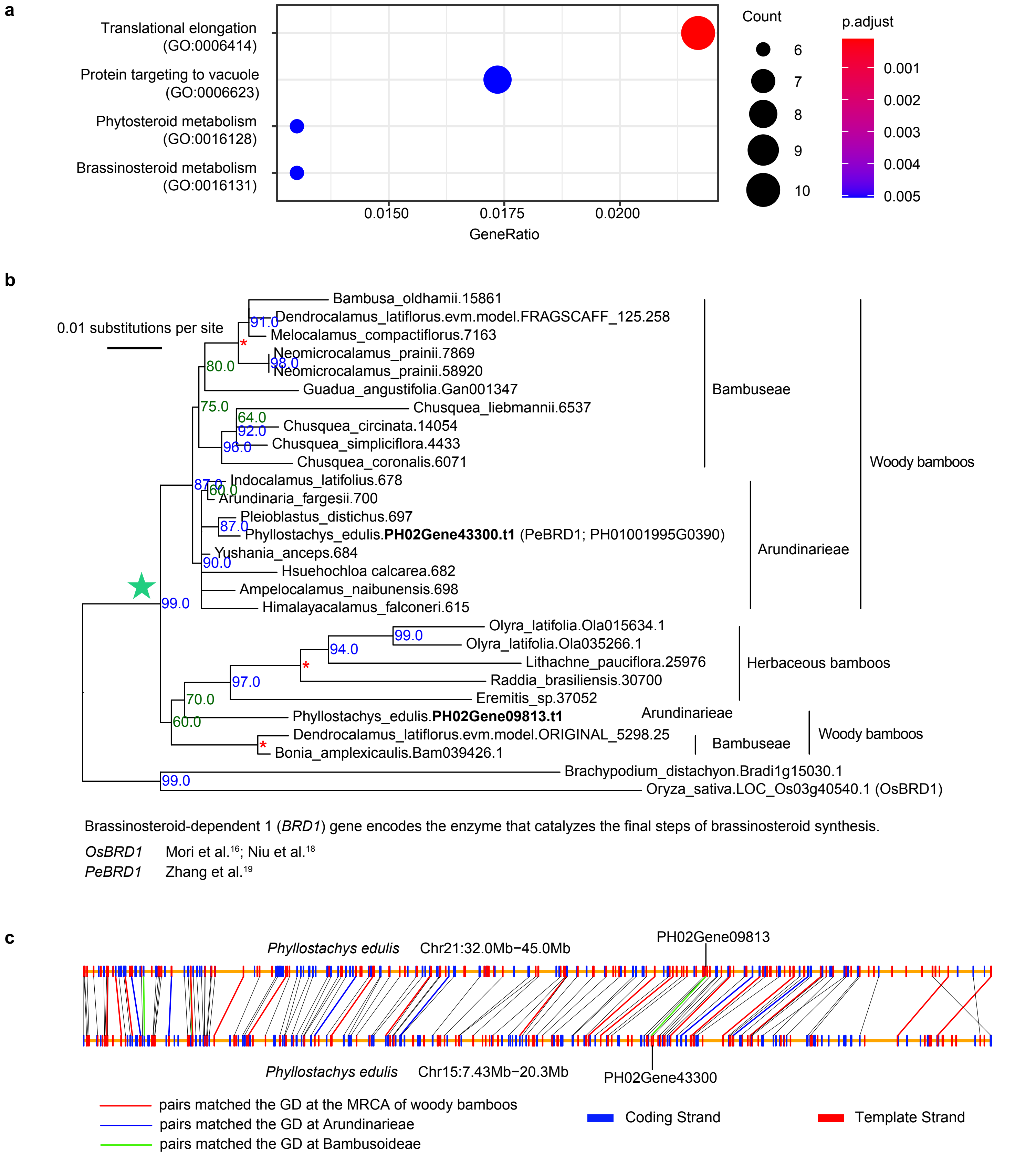

d

Tissue	Expression abundance			SRA in NCBI
	Tpm(*PH02Gene43300*)	Tpm(*PH02Gene09813*)	Tpm (*PH02Gene43300*)/ Tpm (*PH02Gene09813*)	
Inflorescence	6.5703	1.4720	4.4635	SRR1187864
Leaf	22.8147	4.7356	4.8177	SRR2035212
Rhizome Tip	1.7429	1.8872	0.9236	SRR5054402
Lateral Bud	1.3753	2.7209	0.5055	SRR5054408
Shoot Tip	2.0801	0.9732	2.1373	SRR5054405
Shoot 0.2m	10.7463	0.6759	15.9000	SRR6171235
Shoot 0.5m	2.7513	0.3615	7.6116	SRR6171238
Shoot 1m	8.2773	0.2147	38.5526	SRR6171241
Shoot 2m	6.4378	0.9113	7.0646	SRR6171244
Shoot 3m	3.4098	2.4188	1.4097	SRR6171247

Early fast-growing period

Supplementary Figure 38 GO enrichment of genes from *Phyllostachys edulis* mapped at Bambusoideae in (AB)A retention type

(**a**) Dot-plot of GO enrichment, with the size representing the number of genes, the color representing the p-value. The p-value was estimated by the enrichGO function with pAdjustMethod of "BH". Slim related to Brassinosteroid metabolism is enriched, indicating the genes from hybridization might have a role in shoot growth. See parts b and c for the Brassinosteroid-dependent 1 (*BRD1*) gene that encodes an enzyme catalyzing the final steps of brassinosteroid synthesis and has been cloned and functionally verified in dwarf mutant of maize and rice, with the roles in shoot internode elongation[16-17]. (**b**) Gene tree showing the bamboo *BRD1* gene duplicates mapped at Bambusoideae (green star at node). Numbers at nodes represent the support values of bootstrapping 1000 times, of which the maximum support is indicated by a red asterisk (*). (**c**) Illustration of a synteny block from *Phyllostachys edulis*, with the lines in different colors showing distinct phylogenetic positions of gene pairs. The syntenic gene pair (*PH02Gene09813* and *PH02Gene43300*) of *BRD1* in part b are highlighted here. Phylogenetic and genomic analyses of *P. edulis BRD1* paralogs trace a GD mapped at Bambusoideae of the (AB)A retention type and place the gene pair in the synteny block, which also included some genes mapped at the MRCA of WBs, indicating the evolution of WB *BRD1* genes involved the introgressions between HBs and WBs. (**d**) Comparison of the TPM values of *PH02Gene09813* and *PH02Gene43300* genes in different tissues showing their possible functional divergence. The green background indicates the gene expression pattern in the shoots during early fast-growing period. In this period the *PH02Gene43300* gene has higher expression abundances than *PH02Gene09813*, indicating the *PH02Gene43300* gene from one woody bamboo ancestor has a role in bamboo rapid growing and the *PH02Gene09813* gene from the other woody bamboo ancestor with gene flow between herbaceous bamboos might be responsible for the dwarf of herbaceous bamboos. Source data are provided as a Source Data file.

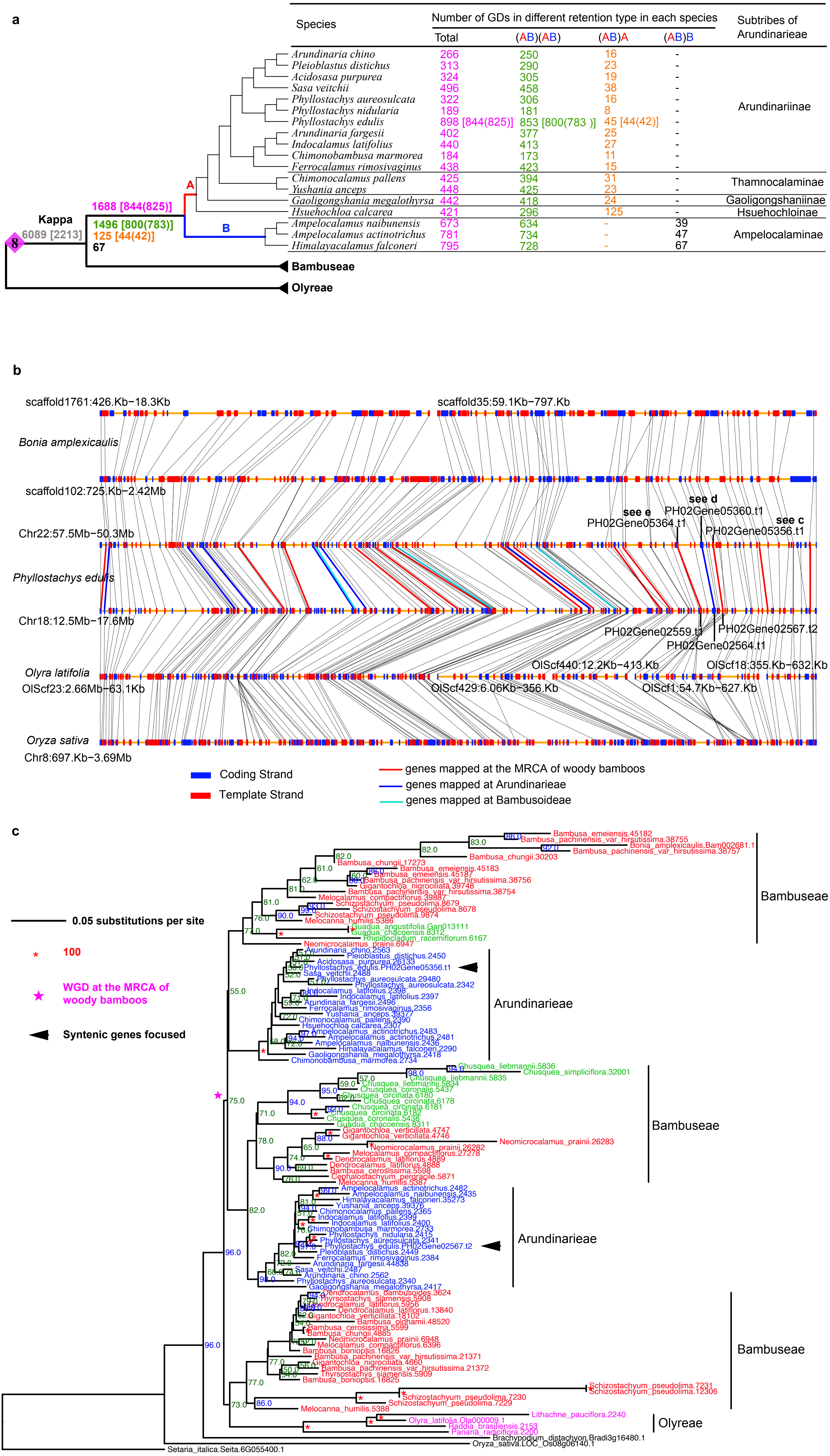
a
Species
Number of GDs in different retention type in each species
Total
(AB)(AB)
(AB)A
(AB)B
Subtribes of Arundinarieae
Arundinaria chino 266 250 16 -
Pleioblastus distichus 313 290 23 -
Acidosasa purpurea 324 305 19 -
Sasa veitchii 496 458 38 -
Phyllostachys aureosulcata 322 306 16 -
Phyllostachys nidularia 189 181 8 -
Phyllostachys edulis 898 [844(825)] 853 [800(783)] 45 [44(42)] -
Arundinaria fargesii 402 377 25 -
Indocalamus latifolius 440 413 27 -
Chimonobambusa marmorea 184 173 11 -
Ferrocalamus rimosivaginus 438 423 15 -
Arundinariinae
Chimonocalamus pallens 425 394 31 -
Yushania anceps 448 425 23 -
Thamnocalaminae
Gaoligongshania megalothyrsa 442 418 24 -
Gaoligongshaniinae
Hsuehochloa calcarea 421 296 125 -
Hsuehochloinae
Ampelocalamus naibunensis 673 634 - 39
Ampelocalamus actinotrichus 781 734 - 47
Himalayacalamus falconeri 795 728 - 67
Ampelocalaminae
A
B
1688 [844(825)]
Kappa
6089 [2213]
8
1496 [800(783)]
125 [44(42)]
67
Bambuseae
Olyreae
b
scaffold1761:426.Kb−18.3Kb
scaffold35:59.1Kb−797.Kb
Bonia amplexicaulis
scaffold102:725.Kb−2.42Mb
see e
see d
see c
PH02Gene05364.t1
PH02Gene05360.t1
PH02Gene05356.t1
Chr22:57.5Mb−50.3Mb
Phyllostachys edulis
Chr18:12.5Mb−17.6Mb
PH02Gene02559.t1
PH02Gene02567.t2
PH02Gene02564.t1
Olyra latifolia
OlScf23:2.66Mb−63.1Kb
OlScf440:12.2Kb−413.Kb
OlScf18:355.Kb−632.Kb
OlScf429:6.06Kb−356.Kb
OlScf1:54.7Kb−627.Kb
Oryza sativa
Chr8:697.Kb−3.69Mb
Coding Strand
Template Strand
genes mapped at the MRCA of woody bamboos
genes mapped at Arundinarieae
genes mapped at Bambusoideae
c
0.05 substitutions per site
100
WGD at the MRCA of woody bamboos
Syntenic genes focused
Bambuseae
Arundinarieae
Bambuseae
Arundinarieae
Bambuseae
Olyreae
Phyllostachys_edulis.PH02Gene05356.t1
Phyllostachys_edulis.PH02Gene02567.t2
Brachypodium_distachyon.Bradi3g16480.1
Oryza_sativa.LOC_Os08g06140.1
Setaria_italica.Seita.6G055400.1

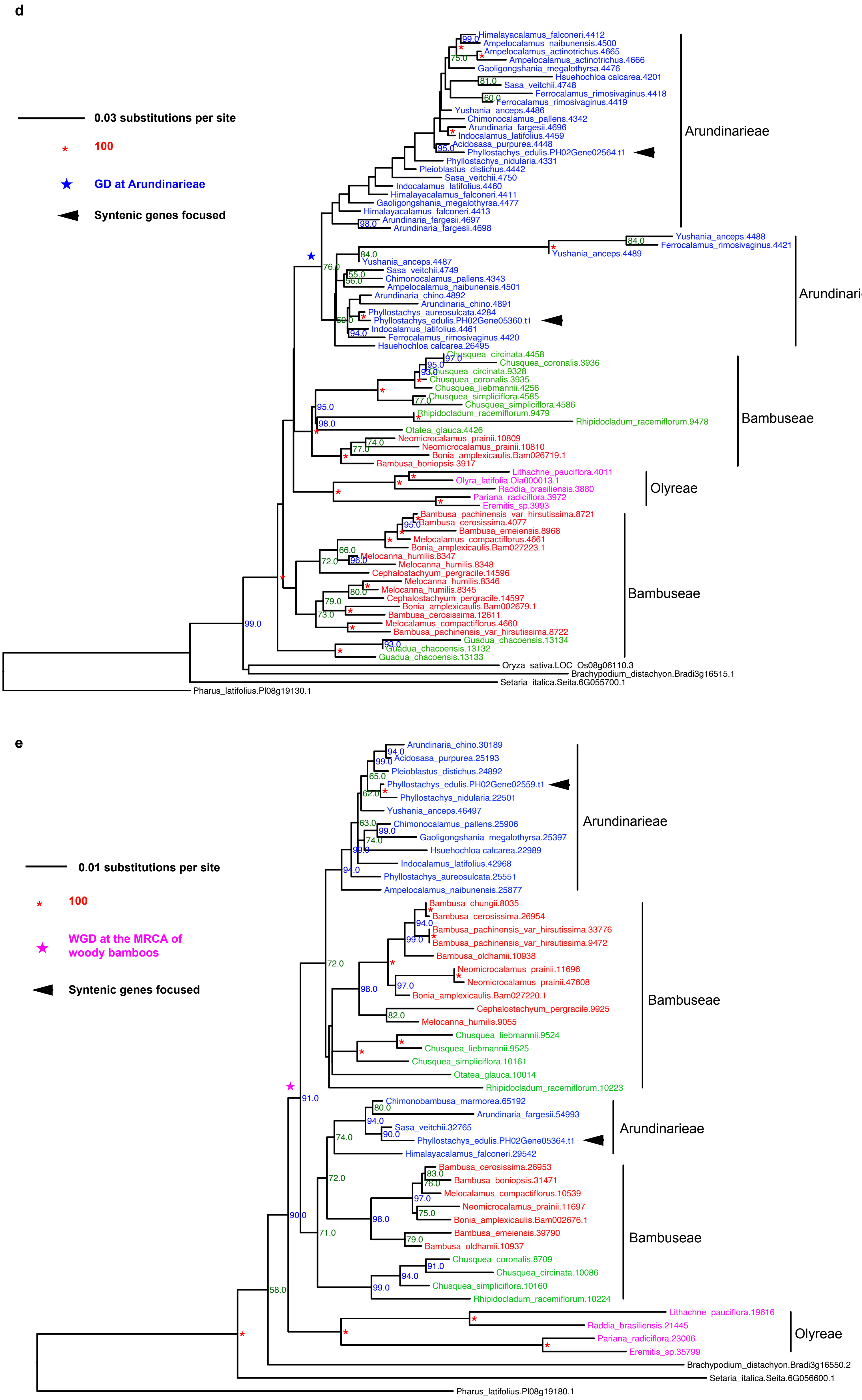

Supplementary Figure 39 Genome synteny summary of GDs at Arundinarieae in different retention type
(**a**) Number of GDs mapped at Arundinarieae in different retention type in each species. Left phylogenetic tree represents a part of the species-tree in Supplementary Fig. 5. Red and blue branches, respectively, represent the last common ancestor of Hsuehochloinae and Arundinariinae subtribes (referred to as A), and the Ampelocalaminae subtribe (referred to as B). Meaning of the numbers in square brackets and round parenthesis, in addition to the color of numbers, are same as that in Supplementary Fig. 31a. The total number of GDs in each retention type is showed by respective colors on the Arundinarieae tribe in the left tree. Number on the woody bamboo ancestor is same as that in Supplementary Fig. 31a. (**b**) A sample of synteny block supporting the kappa event. Meanings of rectangles and lines are same as those in Supplementary Fig. 26c. Red lines show the bamboo orthogroup with *Phyllostachys edulis* syntenic paralogs diverged at the woody bamboo ancestor in the respective phylogeny (in parts c and e). Blue lines represent the orthogroup with *P. edulis* syntenic paralogs diverged at Arundinarieae in the gene tree of part (d).The GDs mapped at Arundinarieae are GDs retained from the kappa event instead of another WGD event specific to Arundinarieae. (**c-e**) Gene trees contained a pair of syntenic genes at the block in part b. Meanings of the coding of Gene ID and numbers behind the nodes in gene tree are same as those in Supplementary Fig. 26d. Gene IDs with arrows are focal syntenic gene pairs as showed in part (b). Source data are provided as a Source Data file.

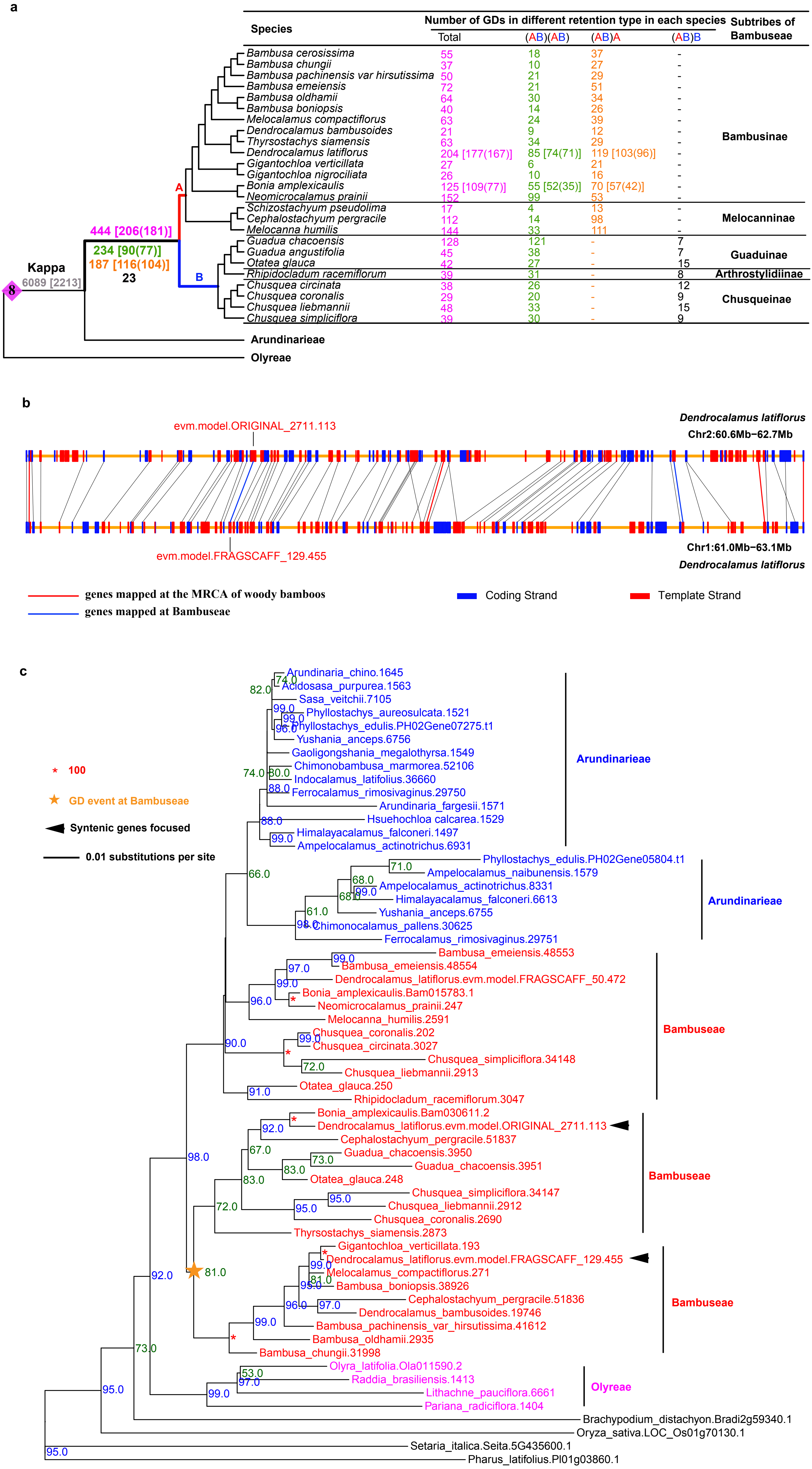

Supplementary Figure 40 Genome synteny summary of GDs at Bambuseae in different retention type
(**a**) Number of GDs mapped at Bambuseae in different retention type in each species. Left phylogenetic tree represents a part of the species-tree in Supplementary Fig. 5. Red and blue colors of branches represent the MRCA of Bambusinae and Melocanninae subtribes (referred to as A), and the MRCA of Guaduinae, Arthrostylidiinae, and Chusqueinae subtribes (referred to as B), respectively. Meaning of the numbers in square brackets and round parenthesis, in addition to the color of numbers, are same as that in Supplementary Fig. 31a. The total number of GDs in each retention type is shown by respective colors on the Bambuseae tribe in the left tree. Number on the woody bamboo ancestor is same as that in Supplementary Fig. 31a. (**b**) A sample of synteny block in *Dendrocalamus latiflorus* genome supporting the kappa event. Meanings of rectangles and lines are same as those in Supplementary Fig. 26c. Red lines show the syntenic paralogs mapped at the woody bamboo ancestor. Blue lines represent the syntenic paralogs mapped at Bambuseae. (**c**) Gene tree showing the duplication of the syntenic genes (evm.model.ORIGINAL_2711.113 and evm.model.FRAGSCAFF_129.455) in part b mapped at Bambuseae (orange star at node). Meanings of the coding of Gene ID and numbers at nodes in gene tree are same as those in Supplementary Fig. 26d. Gene IDs with arrows are focal syntenic gene pairs as shown in part b. Source data are provided as a Source Data file.

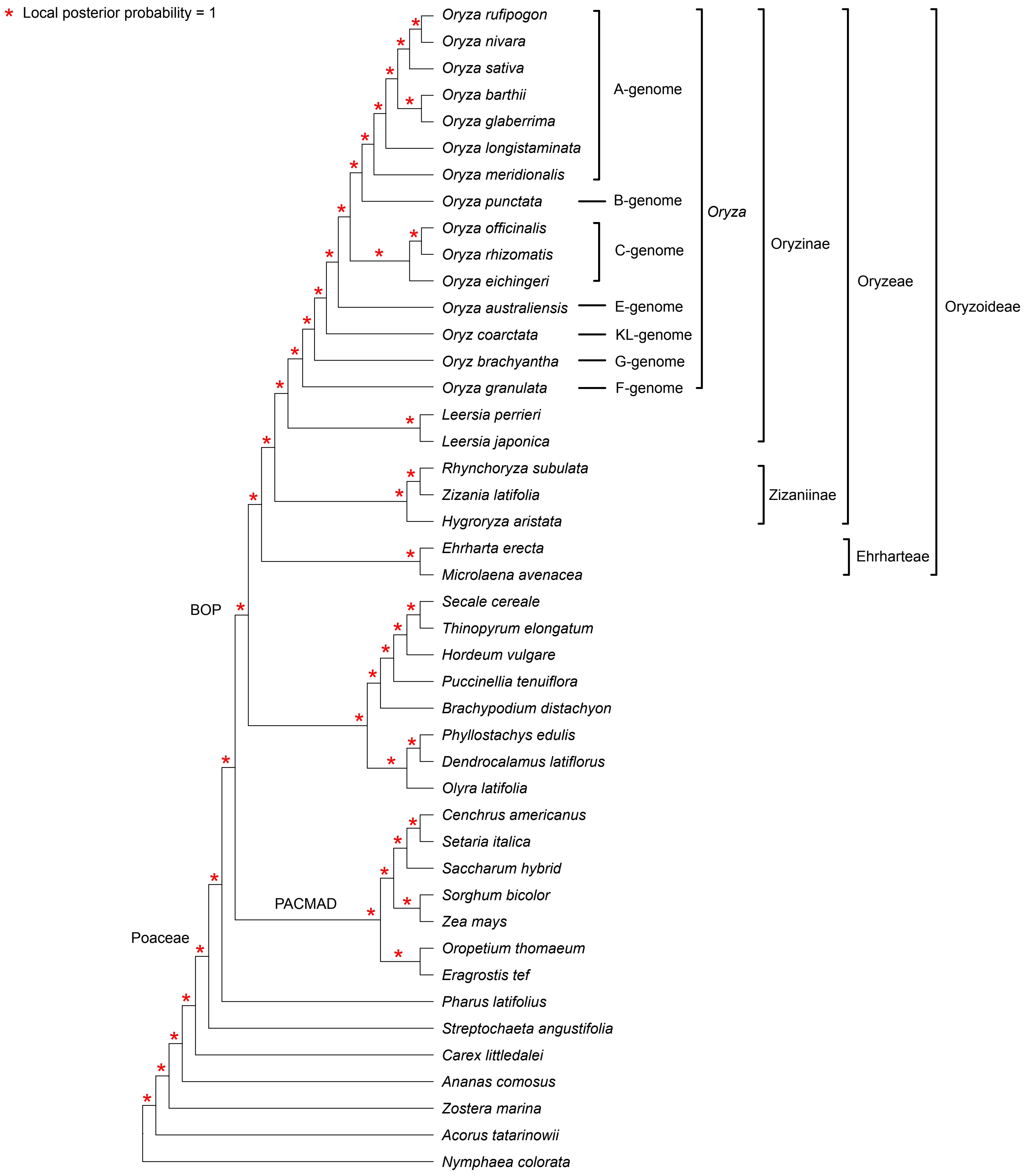

Supplementary Figure 41 Summary of phylogenetic relationships of *Oryza* species
Phylogeny represents the tree inferred from ASTRAL-PRO analyses of 22,829 gene trees with at least two copies in at least one species. Stars on the branches represent the full support in local posterior probability. The genome types (A, B, C, D, E, F, and KL) in *Oryza* are highlighted by different background colors. KL-genome, F-genome and G-genome lineage is placed successively sister to the MRCA of A, B, C, and E genome clades.

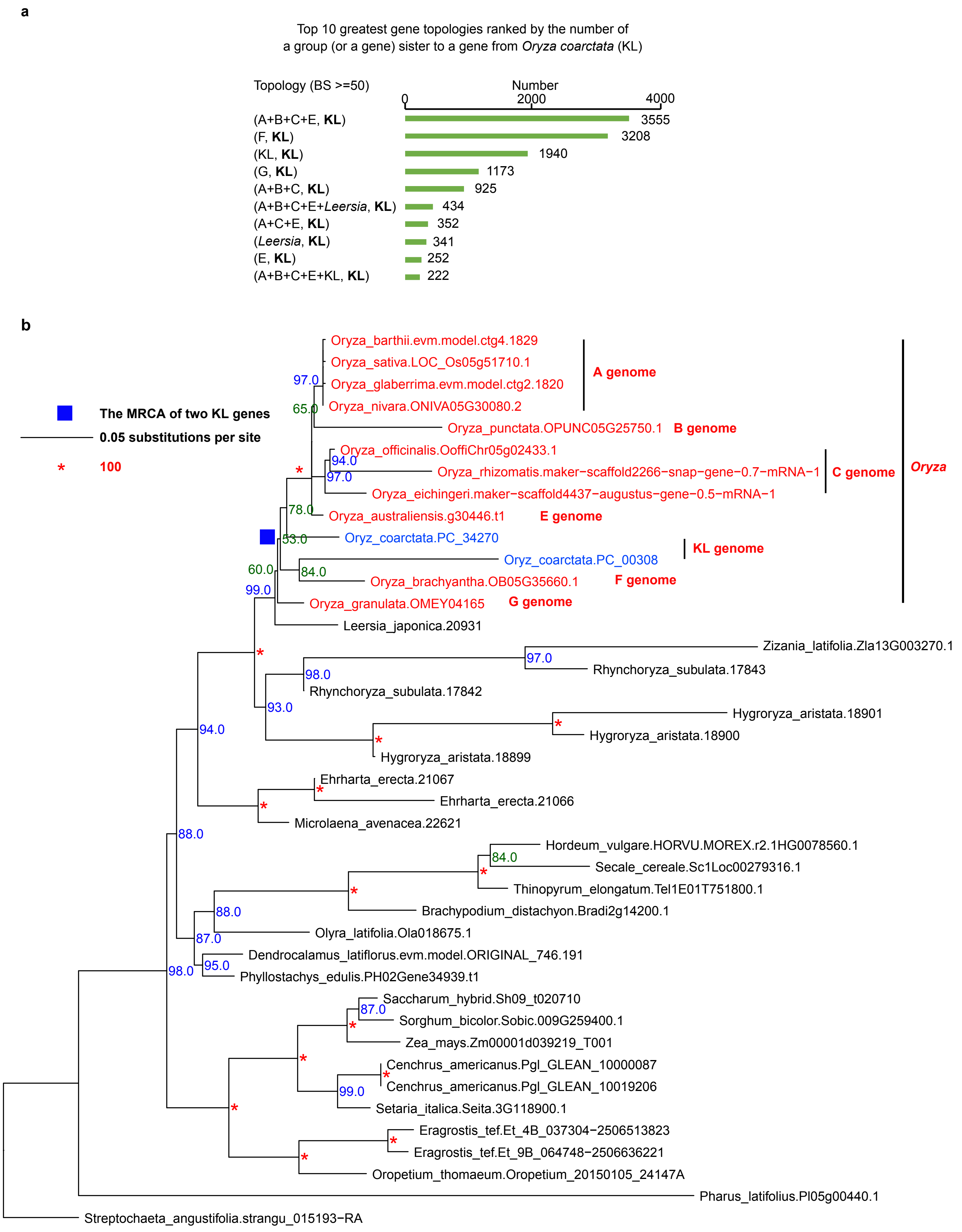

Supplementary Figure 42 Summary of sisterhood of KL genes

(**a**) Top 10 greatest gene topologies ranked by the number of a group (or a gene) sister to a gene from Oryza coarctata (KL). Number to the right of each green bar represents the total number of the topology. The sisterhood between KL and the MRCA of A, B, C, and E genomes receives the greatest number, indicating the MRCA of A, B, C, and E genomes might be one progenitor of KL. The second greatest sisterhood means gene flows between one of KL gene duplicates and its possible progenitor (the F genome). (**b**) An example of gene tree showing the two greatest sisterhoods in part a. Meanings of the coding of Gene ID and numbers at nodes in gene tree are same as those in Supplementary Fig. 26d.

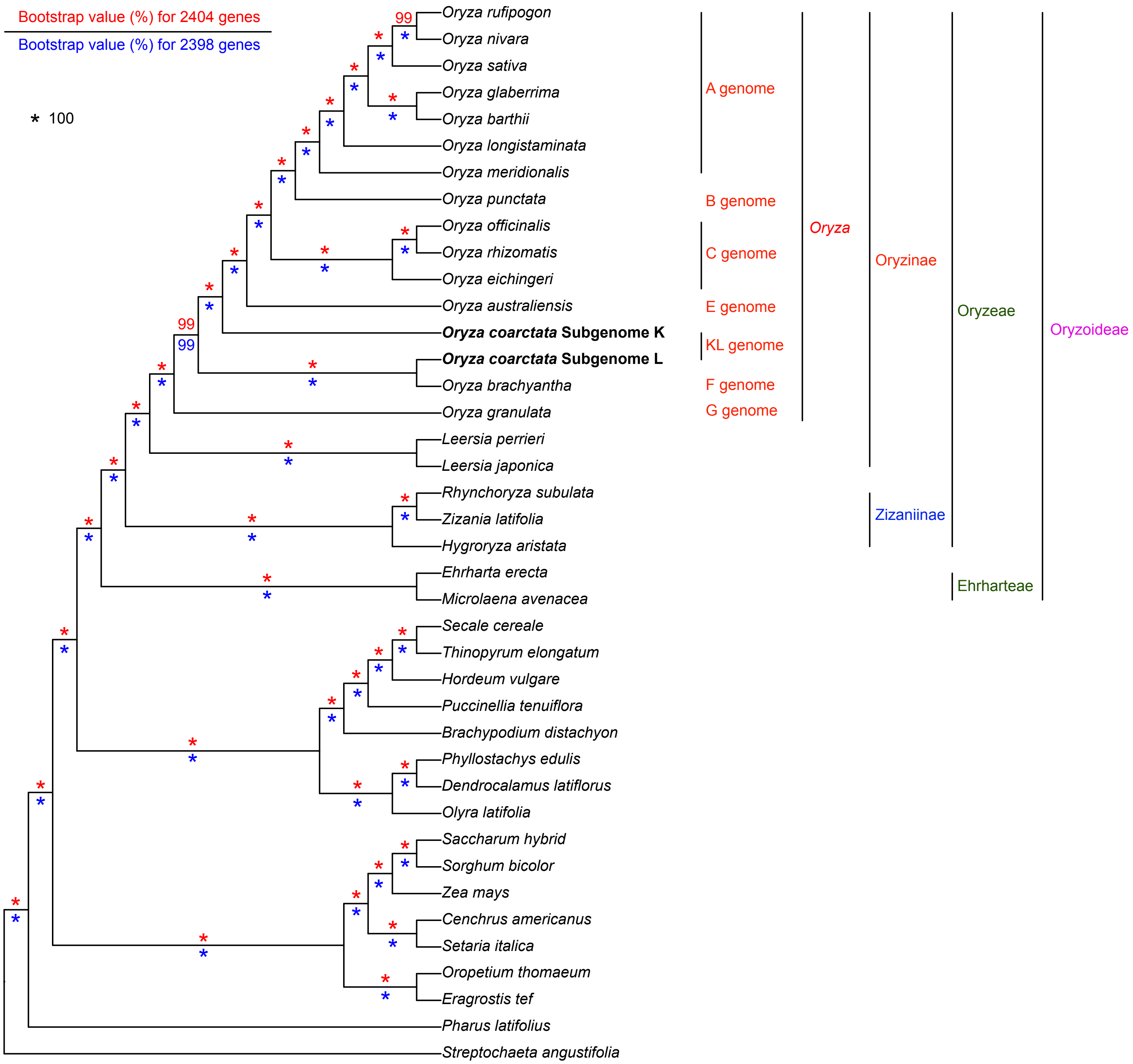

Supplementary Figure 43 Summary of phylogenetic relationships of *Oryza* genomes

In *Oryza*, based on the phylogenetic positions of the subgenomes of the tetraploid KL genome, the subgenome sister to the MRCA of the A, B, C, and E genomes is defined as subgenome-K and the subgenome sister to F genome is defined as subgenome-L. Numbers at branches represent bootstrap values and stars indicate maximum supports. Among the 2,404 gene trees, 2,398 meet the requirements of interspecific collinearity between *Oryza* genomes and/or intraspecific collinearity between the K and L genomes. Both datasets support the sisterhood between the K-subgenome and MRCA of the A, B, C, and E genomes and the sisterhood between the L-subgenome and the F genome.

a

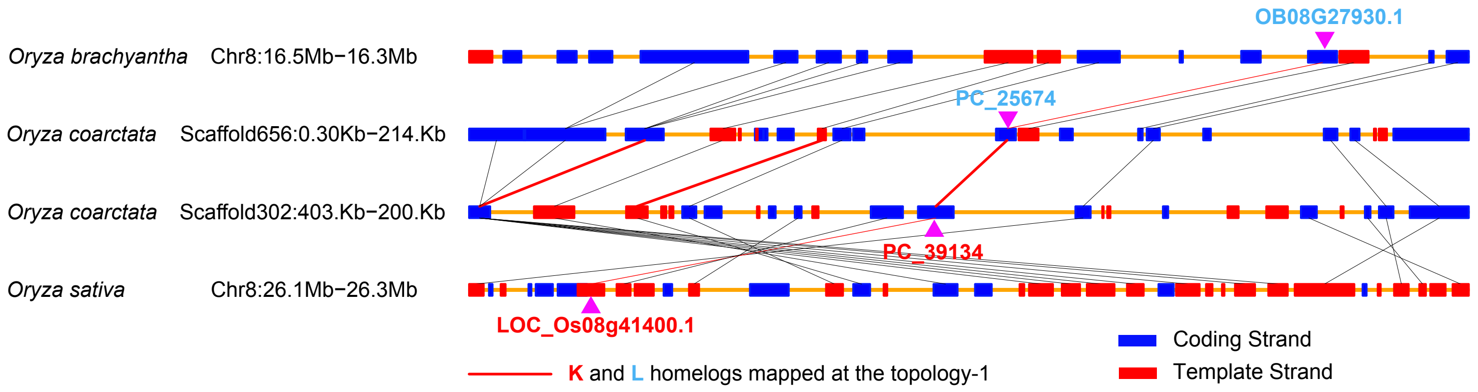

b

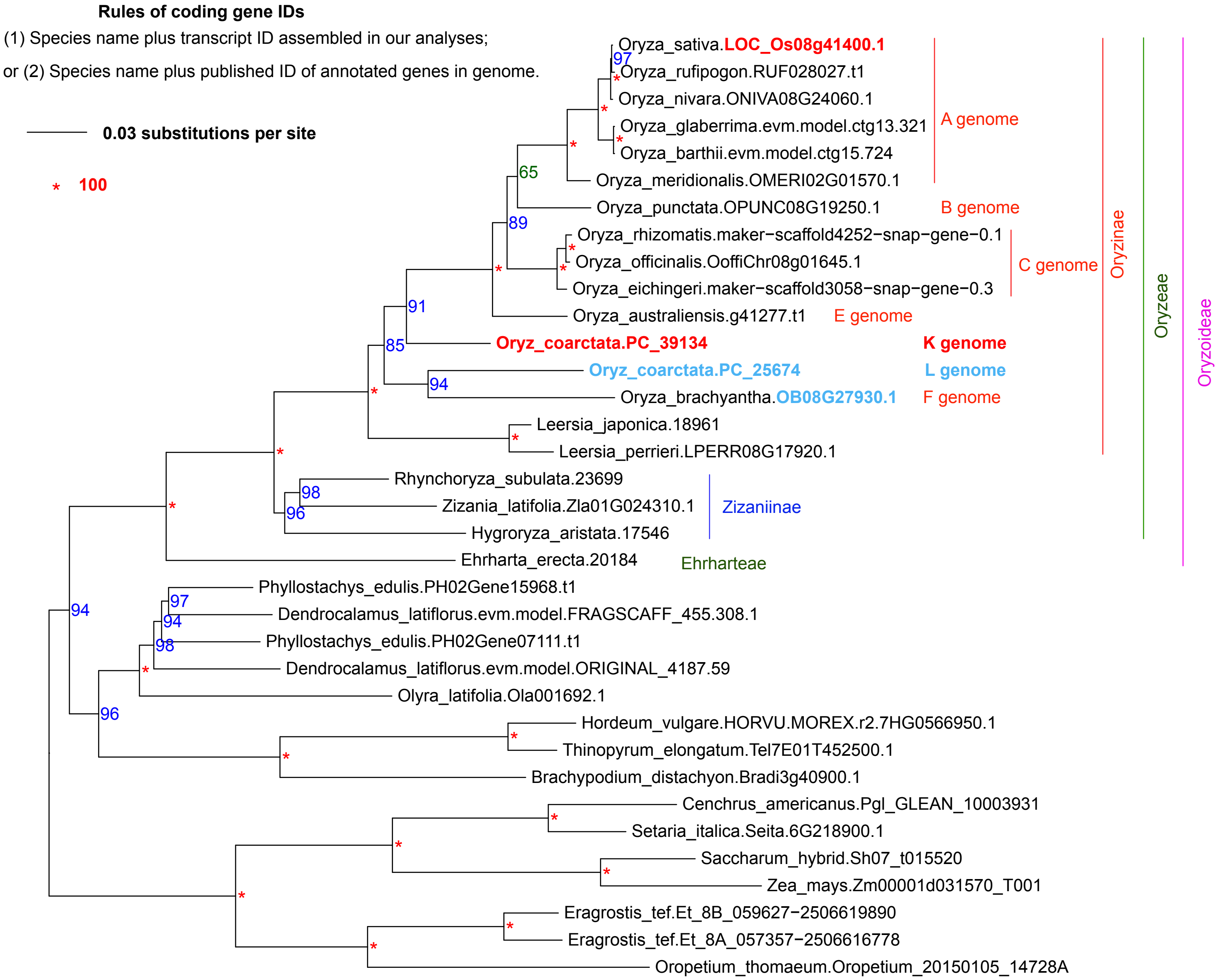

Supplementary Figure 44 A sample of chromosomal collinearity with an orthogroup supporting Topology-1
(**a**) A chromosomal collinear block shared among rice genomes. Meanings of rectangles are same as those in Supplementary Fig. 26c. An orthogroup is highlighted in red and the phylogeny of the orthologous genes is illustrated in part b. (**b**) Gene tree showing the K and L homeolog of Topology-1. Meanings of the coding of Gene ID and numbers behind the nodes in gene tree are same as those in Supplementary Fig. 26d. Gene IDs with arrows are focal syntenic gene pairs as shown in part a. Source data are provided as a Source Data file.

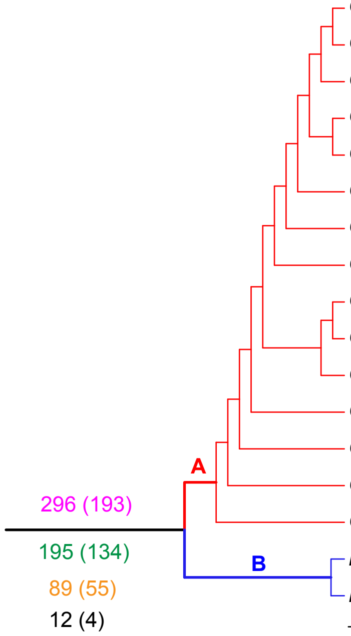

Species	Number of GDs in different retention type in each species				Group	
	Total	(AB)(AB)	(AB)A	(AB)B		
Oryza rufipogon	247 (64)	161 (51)	77 (13)	9	A-genome	*Oryza*
Oryza nivara	229 (62)	150 (44)	70 (18)	9		
Oryza sativa	277 (93)	179 (64)	88 (29)	10		
Oryza glaberrima	267 (101)	173 (73)	84 (28)	10		
Oryza barthii	267 (104)	170 (69)	86 (35)	11		
Oryza longistaminata	235 (61)	156 (42)	71 (19)	8		
Oryza meridionalis	224 (53)	148 (39)	72 (14)	4		
Oryza punctata	253 (66)	175 (52)	71 (14)	7	B-genome	
Oryza officinalis	241 (36)	161 (24)	74 (12)	6	C-genome	
Oryza rhizomatis	237 (62)	159 (44)	73 (18)	5		
Oryza eichingeri	241 (41)	159 (32)	74 (9)	8		
Oryza australiensis	195 (52)	114 (34)	74 (18)	7	E-genome	
Oryza coarctata	156 (8)	101 (5)	51 (3)	4	KL-genome	
Oryza brachyantha	192 (44)	117 (27)	68 (17)	7	F-genome	
Oryza granulata	223 (43)	130 (11)	89 (32)	4	G-genome	
Leersia perrieri	286 (89)	192 (85)	82	12 (4)		*Leersia*
Leersia japonica	171	110	49	12		

(#) number of GDs shared by tandem duplicates

Supplementary Figure 45 Summary of GDs shared by different species in Oryzinae
Number of GDs mapped at Oryzinae in different retention type shared by paralogs in each species. Left phylogenetic tree represents a part of the species-tree in Supplementary Fig. 41. Red and blue colors of branches represent *Oryza* (referred to as A) and *Leersia* (referred to as B), respectively. (AB)(AB) means a pattern of retention of two copies from the A and B lineages. (AB)A means a type of the retention of two copies from the A lineage and retention of only one copy from the B lineage. (AB)B means a type of the retention of two copies from the B lineage and retention of only one copy from the A lineage. Right numbers show the number of GDs contributed by the paralogs from each species in different retention types. The number in brackets represents the number of GDs shared by tandem duplicates. Numbers of GDs in (AB)(AB), (AB)A, (AB)B, and sum of the above 3 types are shown in green, orange, black, and purple colors, respectively. The total number of GDs are noted above a branch in left tree, and that of GDs in different retention types are noted below the branch.

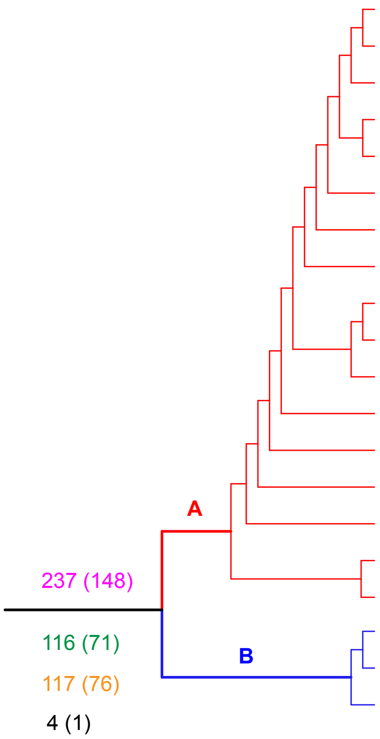

Species	Number of GDs in different retention type in each species				Subtribe
	Total	(AB)(AB)	(AB)A	(AB)B	
Oryza rufipogon	205 (46)	100 (23)	102 (23)	3	
Oryza nivara	198 (56)	93 (30)	102 (26)	3	
Oryza sativa	228 (83)	111 (45)	113 (38)	4	
Oryza glaberrima	211 (83)	100 (42)	107 (41)	4	
Oryza barthii	209 (88)	99 (48)	106 (40)	4	
Oryza longistaminata	182 (46)	86 (25)	93 (21)	3	
Oryza meridionalis	187 (43)	97 (23)	88 (20)	2	
Oryza punctata	203 (56)	100 (29)	100 (27)	3	
Oryza officinalis	185 (25)	87 (11)	97 (14)	1	Oryzinae
Oryza rhizomatis	184 (49)	88 (25)	95 (24)	1	
Oryza eichingeri	192 (29)	92 (13)	98 (16)	2	
Oryza australiensis	184 (46)	94 (26)	86 (20)	4	
Oryza coarctata	134 (10)	65 (7)	68 (3)	1	
Oryza brachyantha	190 (51)	98 (25)	89 (26)	3	
Oryza granulata	190 (31)	94 (16)	93 (15)	3	
Leersia perrieri	205 (75)	92 (22)	110 (53)	3	
Leersia japonica	148	72	72	4	
Rhynchoryza subulata	128	77	47	4	
Zizania latifolia	192 (38)	102 (37)	87	3 (1)	Zizaniinae
Hygroryza aristata	154	82	68	4	

(#) number of GDs shared by tandem duplicates

Supplementary Figure 46 Summary of GDs shared by different species in Oryzeae

Number of GDs mapped at Oryzeae in different retention type in each species. Left phylogenetic tree represents a part of the species-tree in Supplementary Fig. 41. Red and blue colors of branches represent Oryzinae (referred to as A) and Zizaniinae (referred to as B), respectively. As shown below, number in square bracket represents the number of GDs shared by tandem duplicates. Numbers of GDs in (AB)(AB), (AB)A, (AB)B, and sum of the above 3 types are shown in green, orange, black, and purple colors, respectively. The total number of GDs are noted above a branch in left tree, and that of GDs in different retention types are noted below the branch.

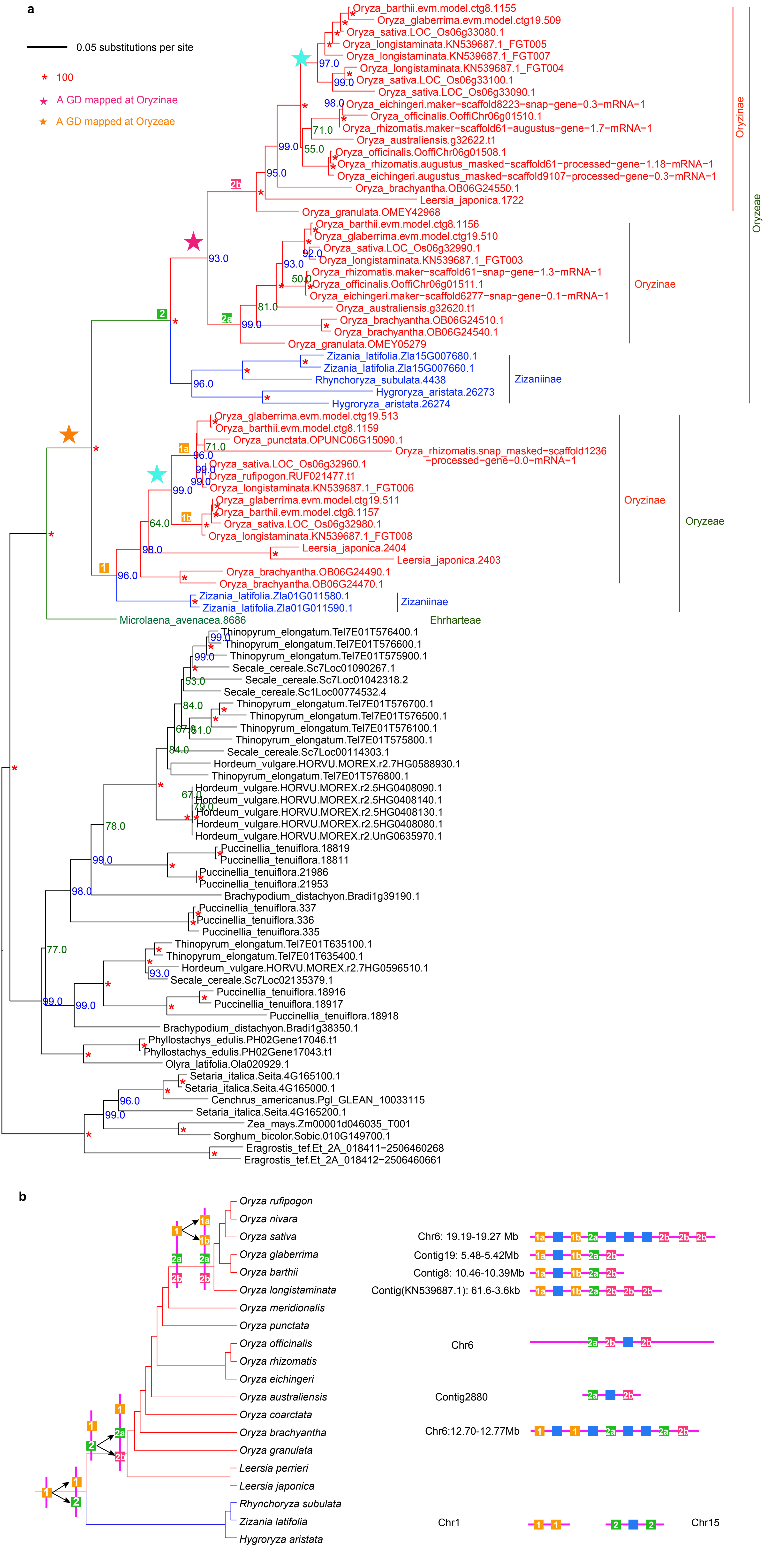

Supplementary Figure 47 Phylogeny and genomic structures of Peroxidase genes
(**a**) Gene tree showing peroxidase gene duplications at Oryzeae (orange star) and at Oryzinae (pink star). Meanings of the coding of Gene ID and numbers behind the nodes in gene tree are same as those in Supplementary Fig. 26d. (**b**) Left: Oryzeae phylogeny from Supplementary Fig. 41. Red branches represent Oryzinae and blue branches represent Zizaniinae. Right: Graph of the order of peroxidase genes (orange, green and red squares) in chromosomes showing tandem duplications shared between Oryzeae. The numbers in squares are same as that in part a. Blue squares represent other genes. A possible landscape of the peroxidase gene evolution is illustrated above branches of the left phylogeny.

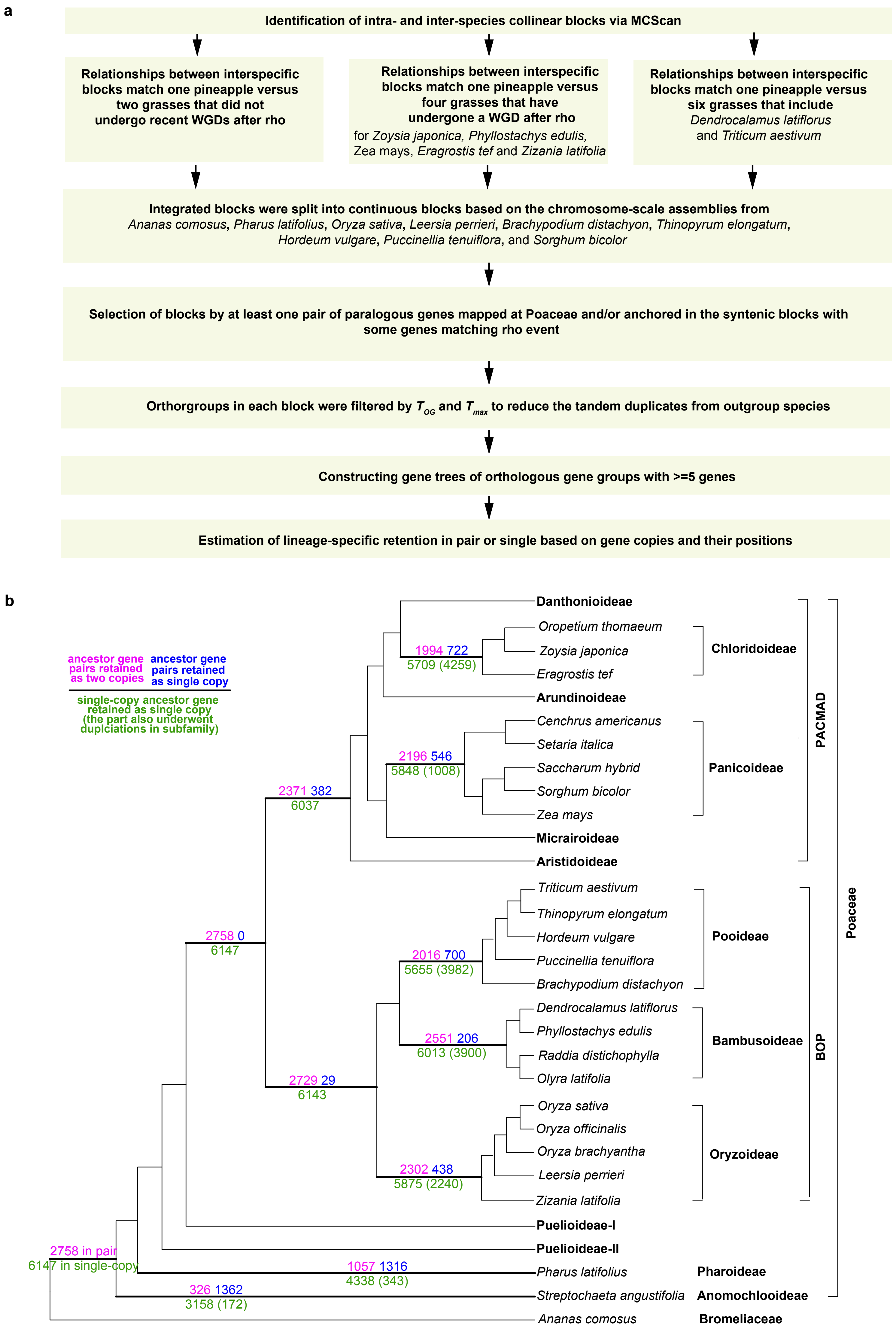

Supplementary Figure 48 Pipeline for estimating ancestral grass genes from rho.
(**a**) A pipeline of inferring grass ancestral genes derived from rho. See the grass genomes used for inference of ancestral genes in the phylogeny of part b. (**b**) Number of ancestral genes from rho in pair and in single-copy among grasses. Pink numbers on the branches represent the number of ancestral gene pairs retained as two copies; blue numbers on the branches represent the number of ancestral gene pairs retained as single copy; and green numbers below the branches represent the number of single-copy ancestral gene retained.

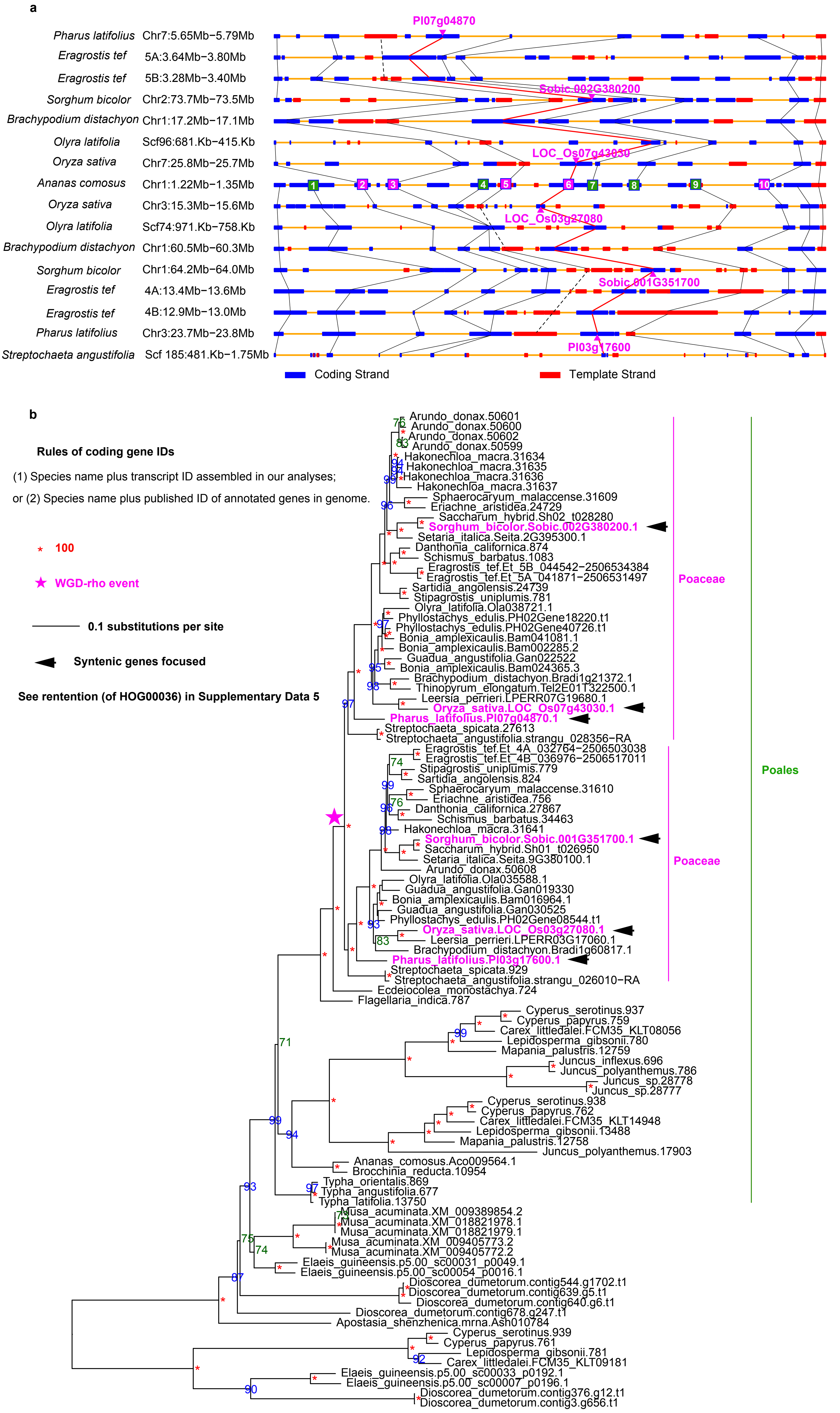

Supplementary Figure 49 A sample of chromosomal collinearity with an orthogroup mapped at Poaceae.
(a) A chromosomal collinear block shared among grass genomes supporting rho event. Meanings of rectangles are same as those in Supplementary Fig. 26c. Genes #1-10 correspond to those in Fig. 6a. An ancestral grass gene in pair is highlighted in red and the phylogeny of the orthologous genes is illustrated in part b. **(b)** Gene tree showing the gene duplication at Poaceae (pink star at node). Meanings of the coding of Gene ID and numbers at nodes in gene tree are same as those in Supplementary Fig. 26d. Gene IDs with arrows are focal syntenic gene pairs as showed in part a. Source data are provided as a Source Data file.

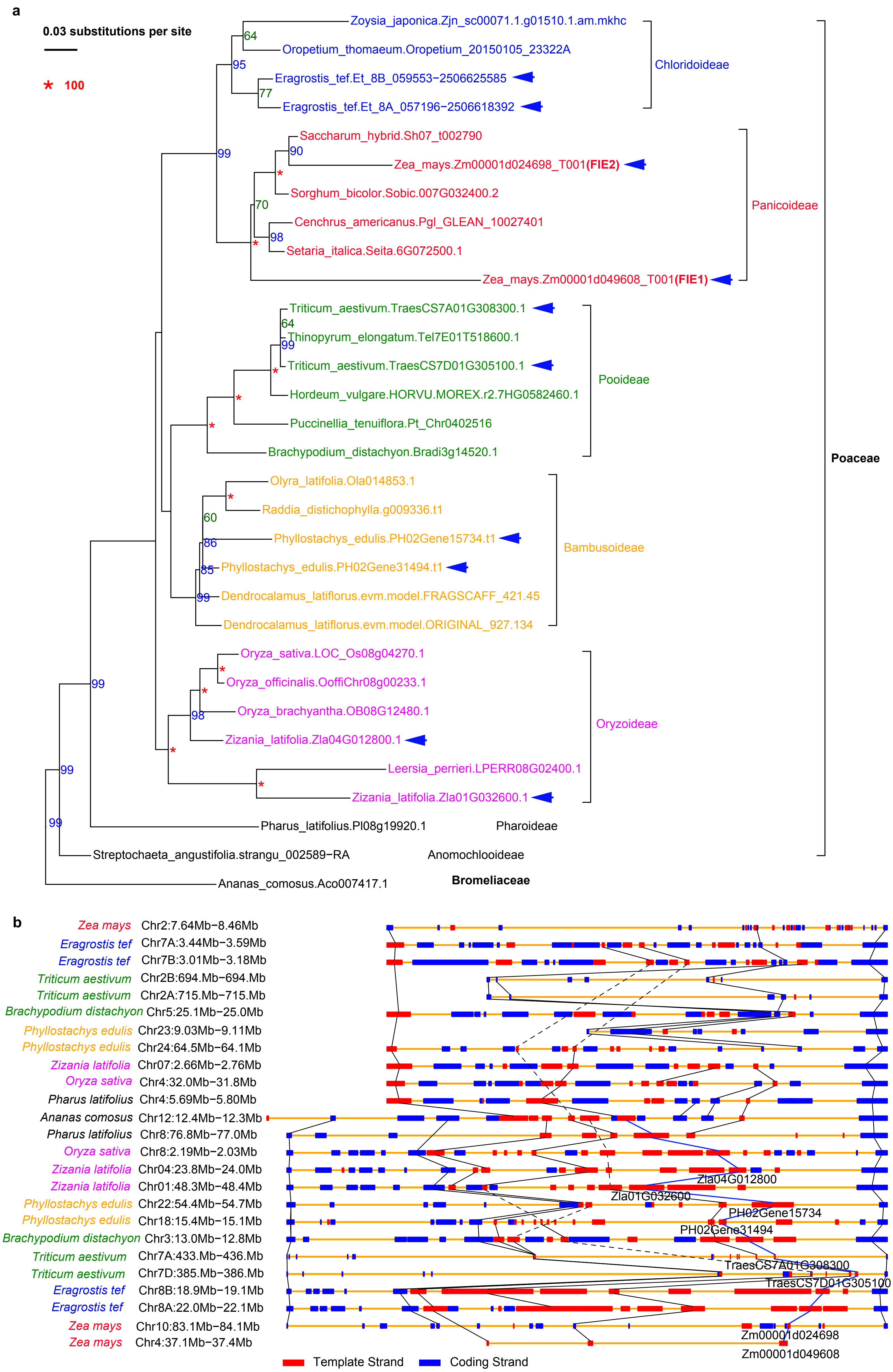

Supplementary Figure 50 A specific gene tree of a type I example of Poaceae orthogroup derived from a single rho-derived copy with lineage specific gene duplications in one or more subfamilies.
(**a**) fertilization independent endosperm (*FIE*) gene tree. The orthogroup includes the maize *FIE1* and *FIE2* genes. The gene tree supports duplications in Bambusoideae, Chloridoideae, Panicoideae, and Pooideae, consistent with lineages WGDs in these subfamilies. Gene ID and numbers to the right of branches in gene tree indicate the same kind of information as those in Supplementary Fig. 26d. See Supplementary Data 5 for the summary of retention and loss of HOG05586 that includes genes encoding FIE. (**b**) A chromosomal collinear block shared among grasses supporting rho. Rectangles and lines represent the same genome features as those in Figure S26c. Blue lines indicate genes in the FIE orthogroup. Source data are provided as a Source Data file.

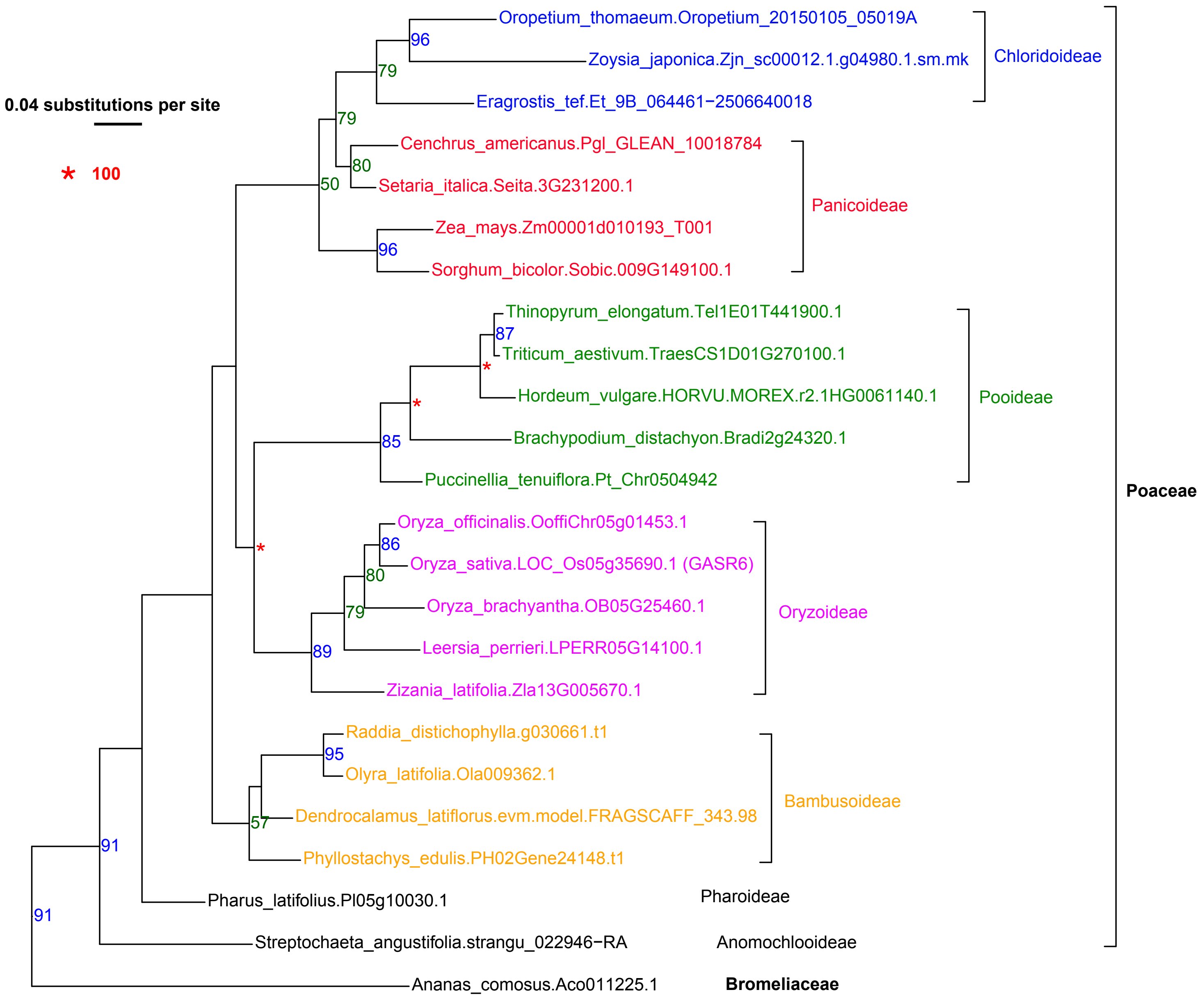

Supplementary Figure 51 A specific gene tree of a type II example, showing a Poaceae orthogroup with single rho-derived copy without detected lineage specific gene duplications in a subfamily.
Gene ID and numbers to the right of branches in gene tree indicate the same kind of information as those in Supplementary Fig. 26d. See Supplementary Data 5 for the summary of retention and loss of HOG00276 that includes genes encoding Gibberellin-regulated GASA/GAST/Snakin family protein precursor.

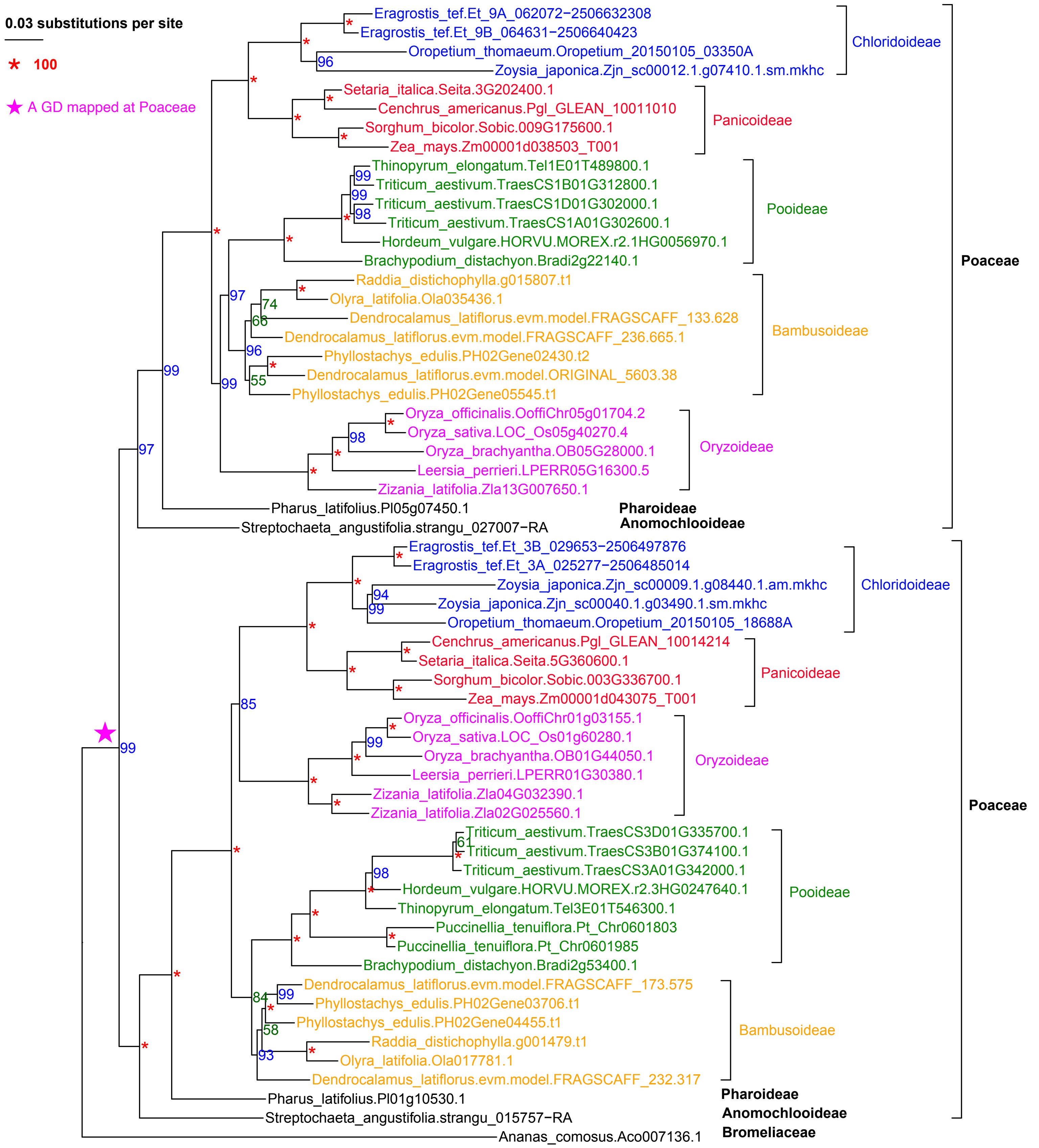

Supplementary Figure 52 A specific gene tree of a type III example showing a Poaceae orthogroup with rho-derived duplicates supported by retention of two copies in each subfamily.
Gene ID and numbers to the right of branches in gene tree indicate the same kind of information as those in Supplementary Fig. 26d. See Supplementary Data 5 for the summary of retention and loss of HOG08744 that includes genes encoding probable LRR receptor-like serine/threonine-protein kinase.

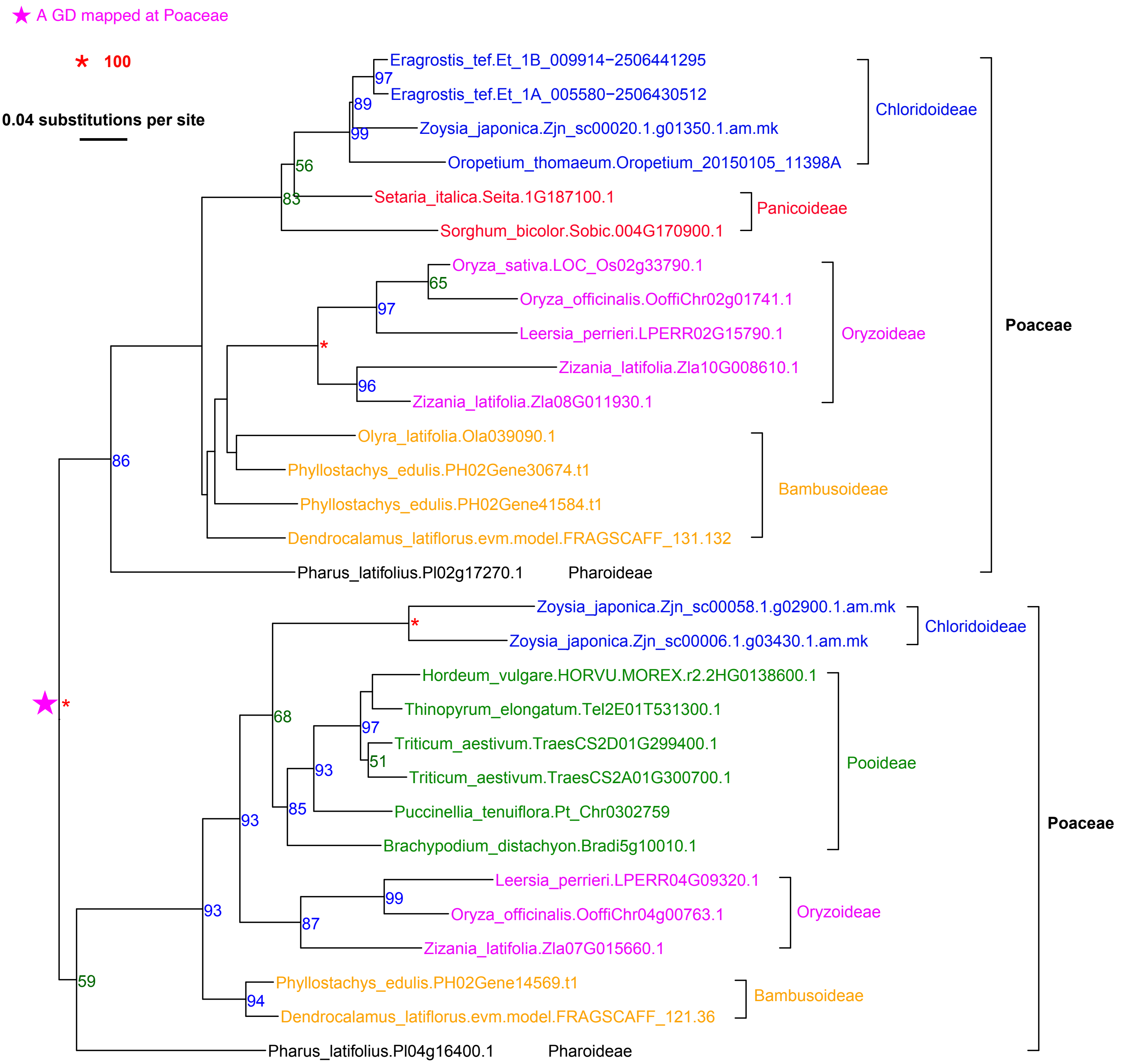

Supplementary Figure 53 A specific gene tree of a type IV example showing a Poaceae orthogroup with rho-derived duplicates in at least one subfamily, but with detected gene losses in at least one other subfamily.
Gene ID and numbers to the right of branches in gene tree indicate the same kind of information as those in Supplementary Fig. 26d. See Supplementary Data 5 for the summary of retention and loss of HOG07941 that includes genes encoding heat shock protein. Chloridoideae and Pooideae retained different copy of the rho-derived duplicates, showing reciprocal loss between subfamilies.

a

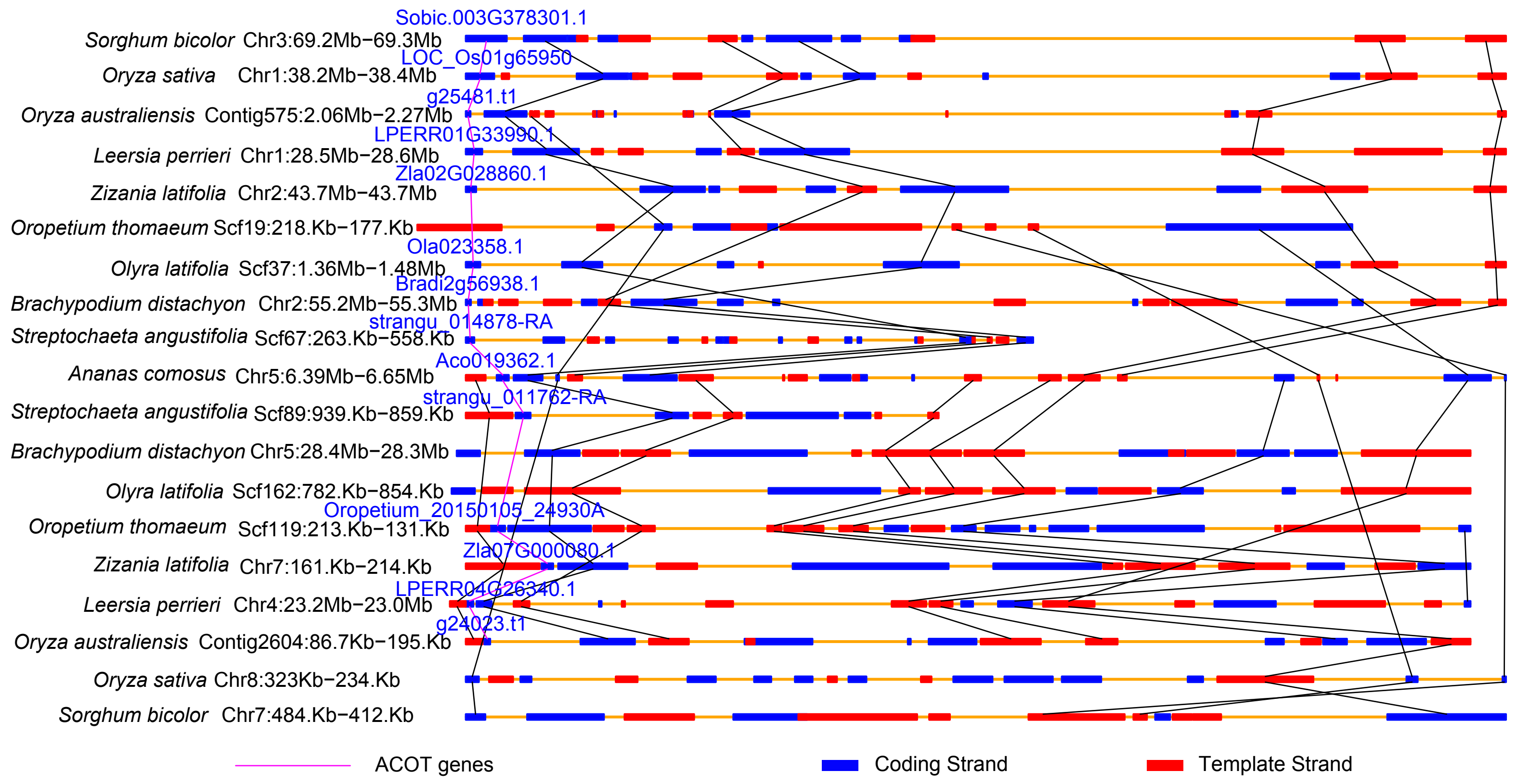

b

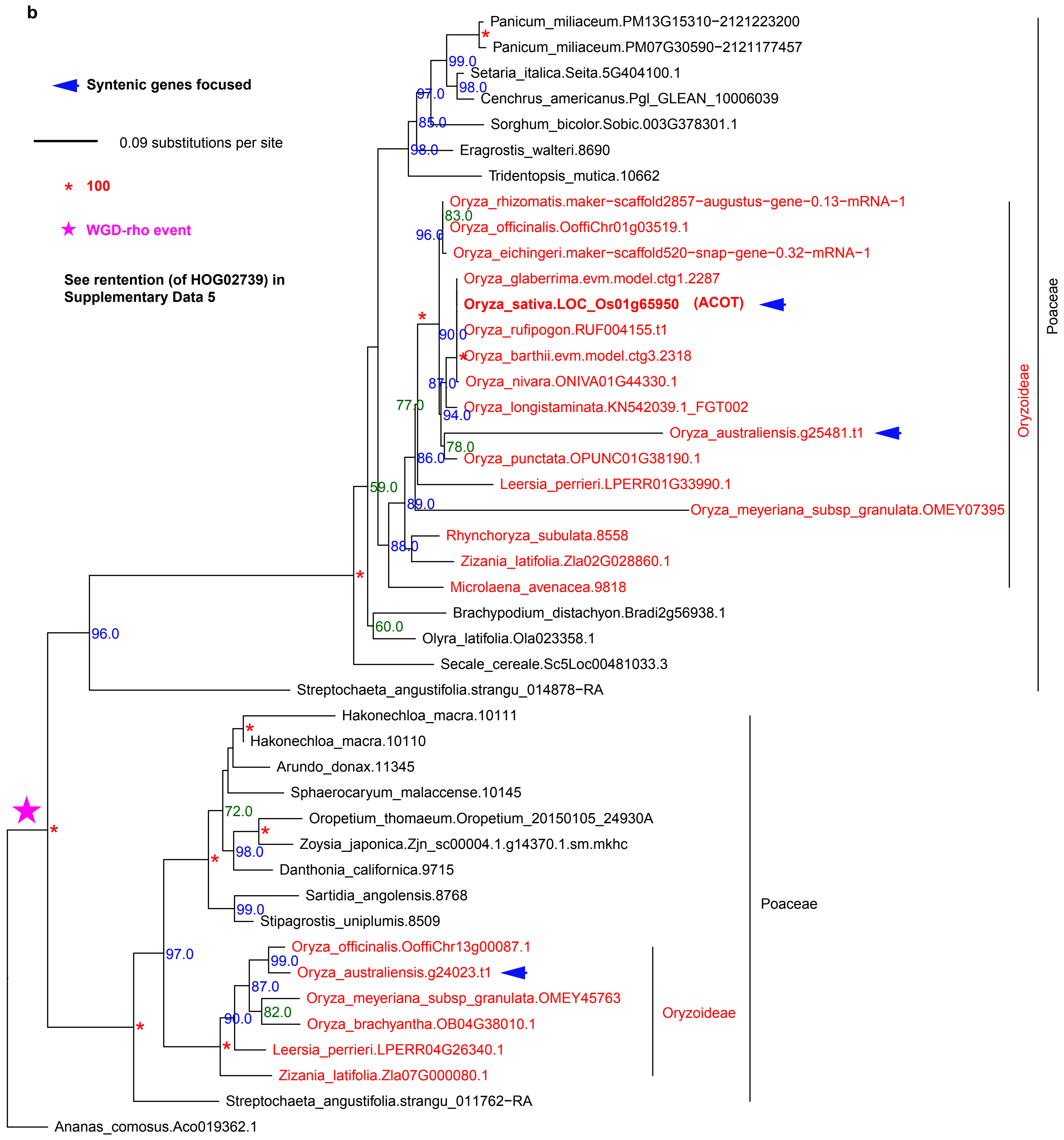

c

Change	Log 2 fold change
in nuclear RNA	1.261805727
in polyA RNA	1.260146794
in Translating Ribosome Affinity Purification (TRAP) polyA RNA	1.841640588
in ribosomes along transcripts (Ribo-seq) RNA	0.737274795

Transcriptional and post-transcriptional change of rice *ACOT* (LOC_Os01g65950) gene under submergence over control

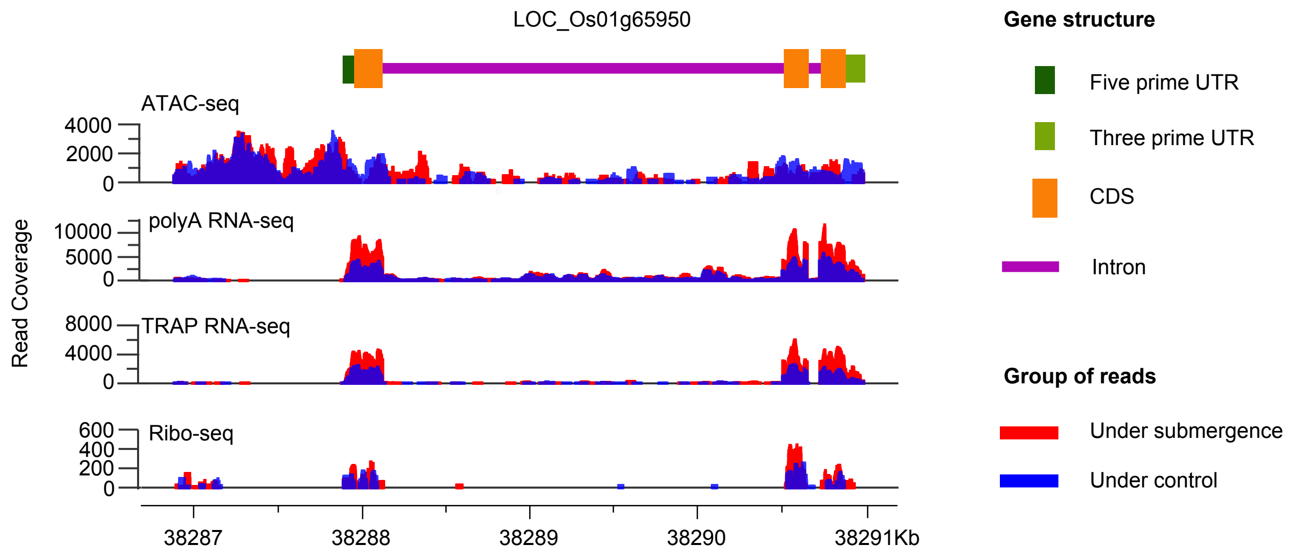

RNA-seq data is retrieved from Reynoso et al.[20] and mapped on the annotated gene (LOC_Os01g65950).

Supplementary Figure 54 Acyl-CoA thioesterase gene evolution in Oryzoideae

(**a**) A chromosomal collinear block shared among grasses supporting rho event. Meanings of rectangles and lines are same as those in Supplementary Fig. 26c. Purple lines indicate the Acyl-CoA thioesterase (*ACOT*) orthogroup. (**b**) A phylogenetic gene tree showing the grass *ACOT* gene duplication mapped at Poaceae (purple star at node). Meanings of the coding of Gene ID and numbers at nodes in gene tree are same as those in Supplementary Fig. 26d. Gene IDs with arrows are focal syntenic gene pairs as shown in part a. (**c**) Summary of the expression abundance of the rice *ACOT* gene (LOC_Os01g65950) in root under submergence and control. (Top) a table showing the changes in nuclear RNA, in polyA RNA, in TRAP polyA RNA, and in Ribo-seq RNA; (Bottom) comparison of the coverage of sequenced reads from four different datasets against the rice *ACOT* gene. See the meaning of rectangles in the right of graph. Source data are provided as a Source Data file.

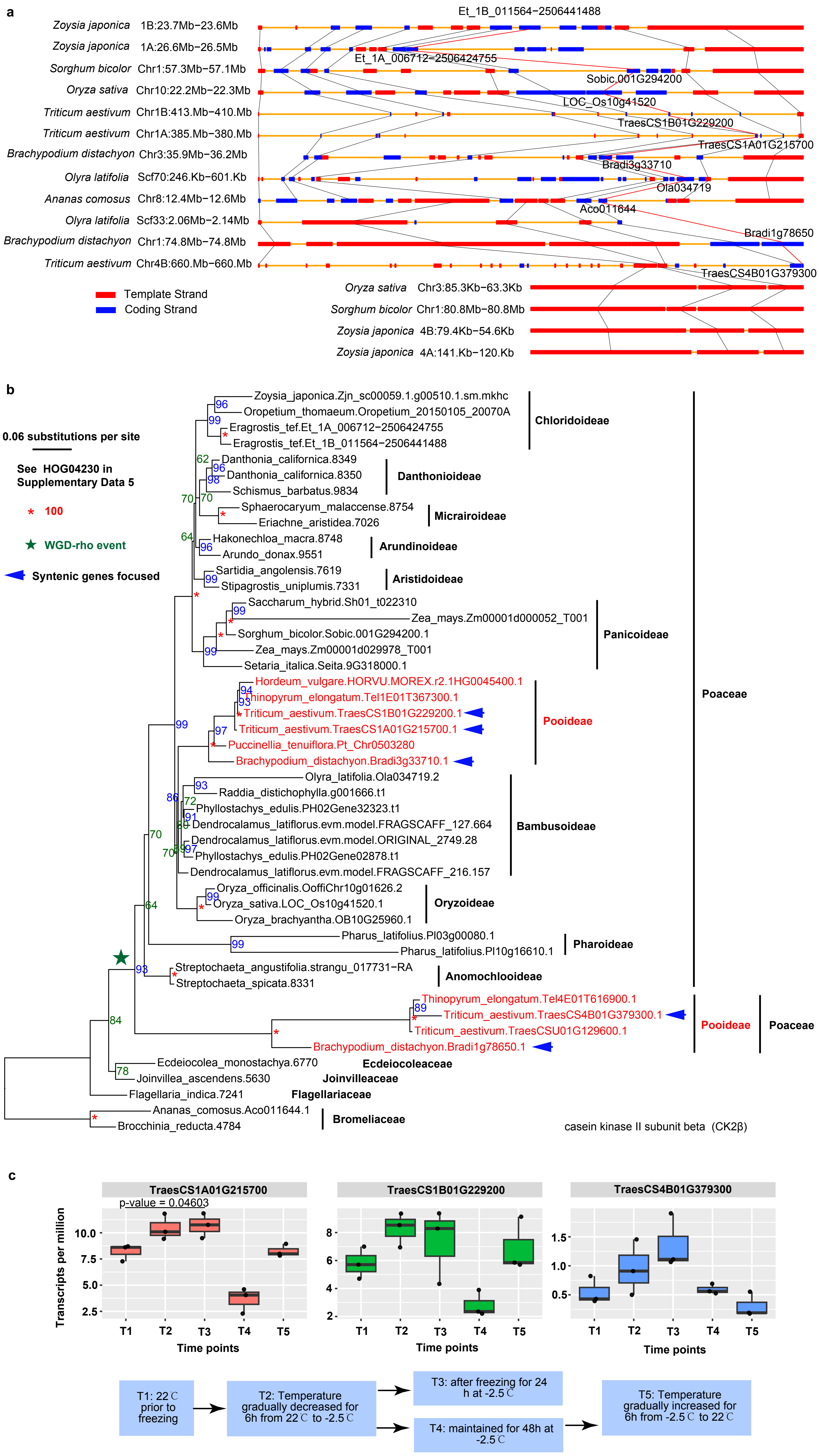

Supplementary Figure 55 Evolution of the casein kinase II gene in Pooideae

(**a**) A chromosomal collinear block shared among grasses supporting rho. Rectangles and lines represent the same genome features as those in Supplementary Fig. 26c. Red lines indicate genes in the casein kinase II subunit beta (CK2β) orthogroup. (**b**) A phylogenetic gene tree showing the grass CK2β gene duplication mapped at Poaceae (green star at node). Gene ID and numbers to the right of branches in the gene tree are same as those in Supplementary Fig. 26d. Gene IDs with arrows are focal syntenic gene pairs as shown in part a. (**c**) Comparison of expression levels of three wheat *CK2*β genes showing similar patterns during temperature decrease. Left: boxplots of gene expression profiling of TraesCS1A01G215700 in wheat stem at five time points showing an up-regulation after temperature was decreased to -2.5 degrees, and following further freezing for 24 h. Middle: boxplots of gene expression profiling of TraesCS1B01G229200 showing an up-regulation pattern similar to the TraesCS1A01G215700. Right: boxplots of gene expression profiling of TraesCS4B01G379300 showing a similar pattern to the TraesCS1A01G215700 after the temperature decrease and the further freezing for 24 h, although the extent of increase was less; in addition, the expression was further reduced at T5 after temperature was increased after freezing, unlike the other two genes. It is possible that CK2β has a positive regulatory role in response to cold stress. The fifteen wheat RNA-Seq datasets in boxplots were retrieved from NCBI (SRR22346048 through SRR22346062); the flow diagram below boxplots illustrates the five time points (see details in Yao et al.[21]). P-value was estimated by t.test (alternative ="two.sided") in R. The center line in box-plot marks the median. Source data are provided as a Source Data file.

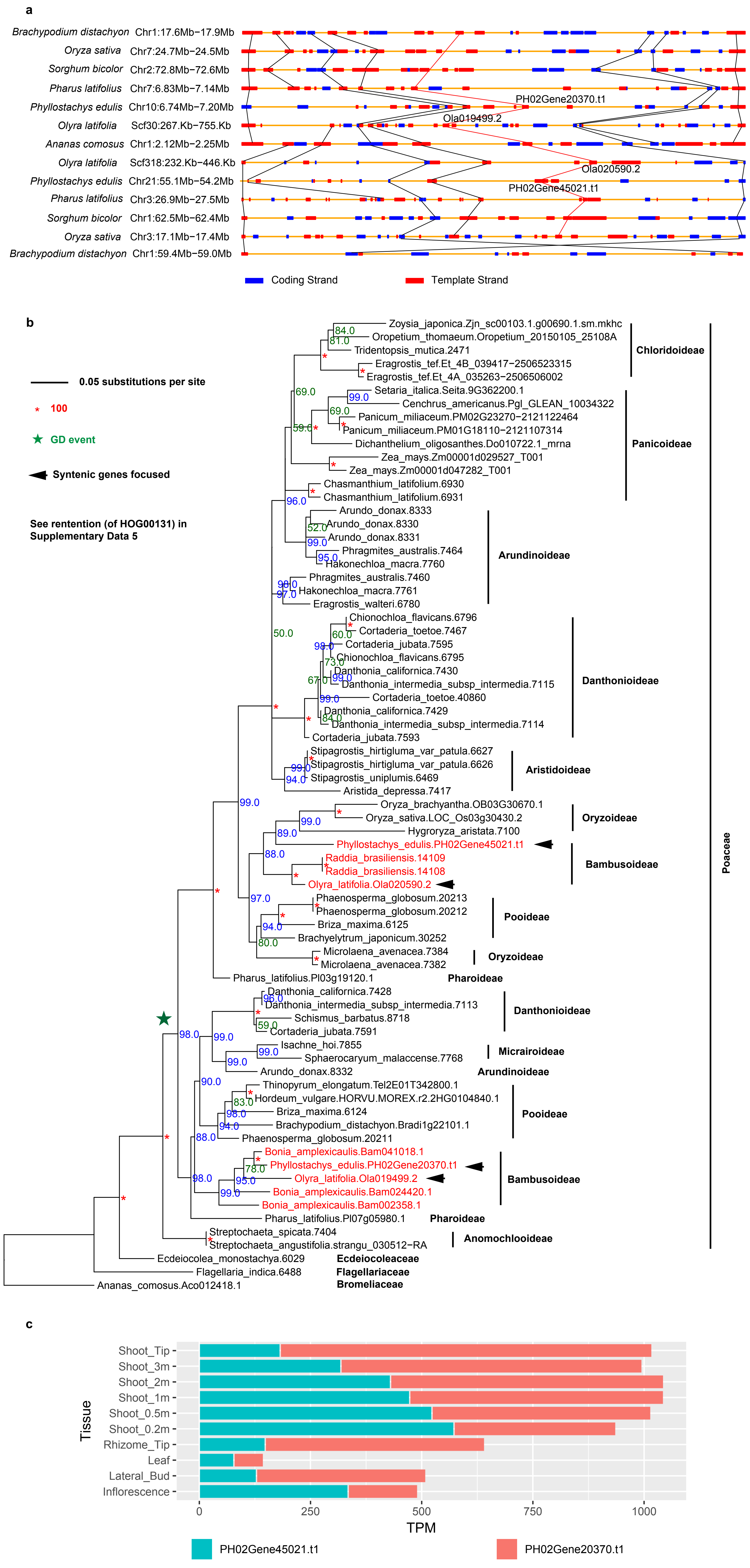

Supplementary Figure 56 SPIRAL1 gene evolution in Bambusoideae
(**a**) A chromosomal collinear block shared among grasses supporting rho event. Meanings of rectangles and lines are same as those in Supplementary Fig. 26c. Red lines indicate the SPIRAL1 (*SRP1*) orthogroup. (**b**) A phylogenetic gene tree showing the grass *SPR1* gene duplication mapped at Poaceae (green star at node). Meanings of the coding of Gene ID and numbers at nodes in gene tree are same as those in Supplementary Fig. 26d. Gene IDs with arrows are focal syntenic gene pairs as shown in part a. (**c**) Comparison of TPM values of *SPR1* genes (*PH02Gene45021.t1* and *PH02Gene20370.t1*) from *Phyllostachys edulis* in different tissues implicating functional divergence of this gene pair. Source data are provided as a Source Data file.

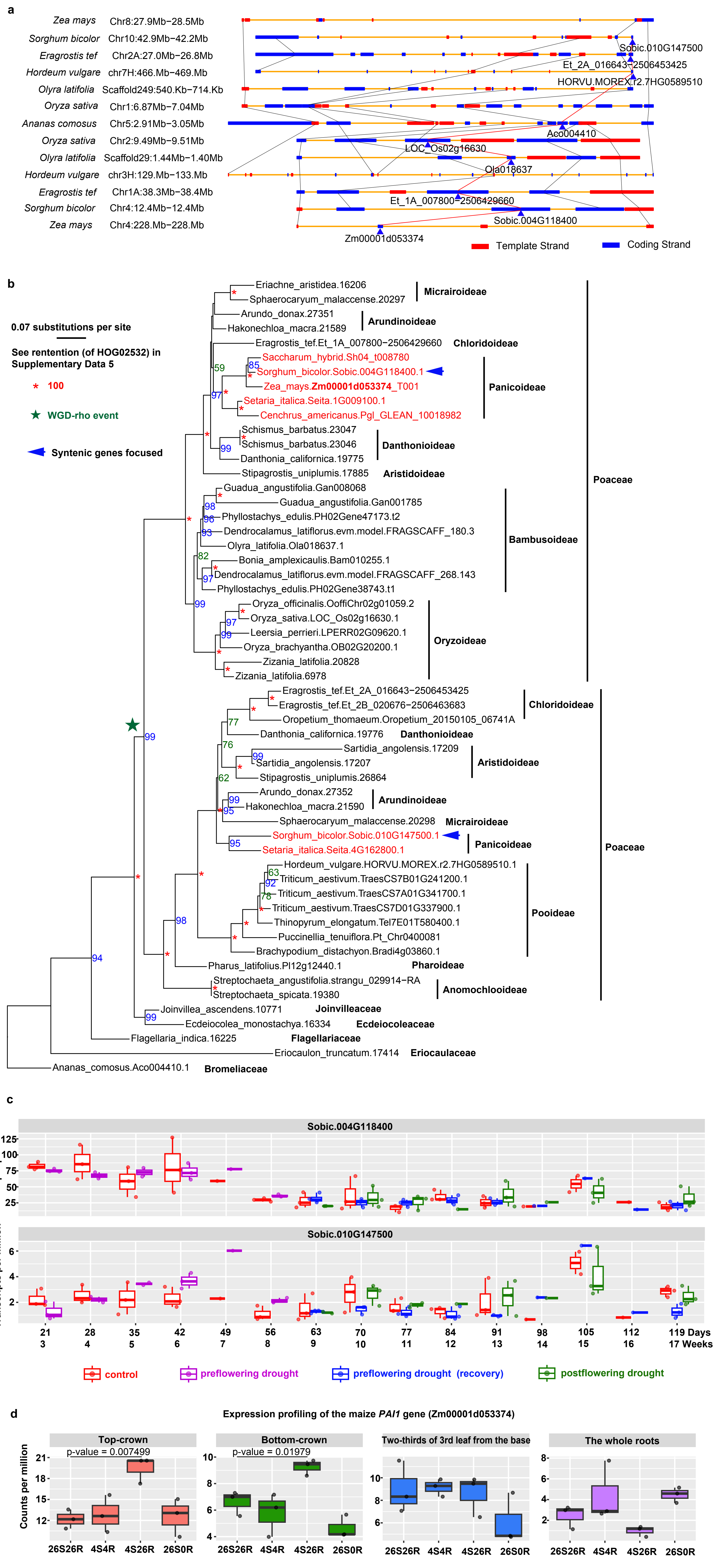

26S26R: normal growth condition at 26 °C. 4S4R: whole-plant chilling at 4 °C. 4S26R: shoot-only chilling with leaves at 4 °C and roots at 26 °C. 26S0R: root-only chilling with leaves at 26 °C and roots at 0 °C.

Supplementary Figure 57 Evolution of the phosphoribosylanthranilate isomerase 1 gene in Panicoideae
(**a**) A chromosomal collinear block shared among grasses supporting rho. Rectangles and lines represent the same genome features as those in Supplementary Fig. 26c. Red lines indicate genes in the phosphoribosylanthranilate isomerase 1 (*PAI1*) orthogroup. (**b**) A phylogenetic gene tree showing the grass *PAI1* gene duplication mapped at Poaceae (green star at node). Gene ID and numbers to the right of branches in the gene tree are same as those in Supplementary Fig. 26d. Gene IDs with arrows are focal syntenic gene pairs as shown in part a. (**c**) Boxplots of the expression profiling of the *PAI1* gene in sorghum leaves under control and drought treatment for 15 weeks. The RNA-Seq data were retrieved from the NCBI database (SRR8742861 through SRR8742957)[22]. Red plots: control (from 3rd week to 17th week); purple plots: preflowering drought (from 3rd week to 8th week); blue plots: preflowering drought but recovery after flowering (from 9th week to 17th week); green plots: postflowering drought (from 9th week to 17th week). Top: boxplots of the expression profiling of *Sobic.004G118400*. Bottom: boxplots of the expression profiling of *Sobic.010G147500*. The sorghum *PAI1* syntenic paralogs show different expression patterns under drought. (**d**) Boxplots of the expression profiling of the maize *PAI1* gene (*Zm00001d053374*; see GSE167881 in GEO series of NCBI) under chilling show an upregulated expression pattern in the top and bottom crown, which includes meristematic tissues for formation of new shoot and root tissues[23]. P-value was estimated by t.test (alternative ="two.sided") in R. The center line in box-plot marks the median. Together, the patterns in sorghum and maize imply that *PAI1* gene might contribute to plant acclimation under drought and chilling stresses. Source data are provided as a Source Data file.

Supplementary References

1. Li, H.-T. *et al.* Origin of angiosperms and the puzzle of the Jurassic gap. *Nat. Plants* **5**, 461-470 (2019).
2. Coiffard, C., Kardjilov, N., Manke, I. & Bernardes-de-Oliveira, M. E. C. Fossil evidence of core monocots in the Early Cretaceous. *Nat. Plants* **5**, 691-696 (2019).
3. Wu, Y., You, H.-L. & Li, X.-Q. Dinosaur-associated Poaceae epidermis and phytoliths from the Early Cretaceous of China. *Natl. Sci. Rev.* **5**, 721-727 (2017).
4. Crepet, W. L. & Feldman, G. D. The earliest remains of grasses in the fossil record. *Am. J. Bot.* **78**, 1010-1014 (1991).
5. Iles, W. J. D., Smith, S. Y., Gandolfo, M. A. & Graham, S. W. Monocot fossils suitable for molecular dating analyses. *Bot. J. Linn. Soc.* **178**, 346-374 (2015).
6. Prasad, V. *et al.* Late Cretaceous origin of the rice tribe provides evidence for early diversification in Poaceae. *Nat. Commun.* **2**, 480 (2011).
7. Strömberg, C. A. E. Decoupled taxonomic radiation and ecological expansion of open-habitat grasses in the Cenozoic of North America. *Proc. Natl. Acad. Sci. U. S. A.* **102**, 11980-11984 (2005).
8. Walther, H. & Kvaček, Z. Early Oligocene flora of Seifhennersdorf (Saxony). *Acta Mus. Nat. Pragae, Ser. B, Hist. Nat.* **63**, 85–174 (2007).
9. Manchester, S. R. Update on the megafossil flora of Florissant, Colorado. *Denver Museum of Nature and Science* **4**, 137 (2001).
10. Leng, Q. & Friis, E. M. Angiosperm leaves associated with Sinocarpus infructescences from the Yixian Formation (mid-Early Cretaceous) of NE China. *Plant Syst. Evol.* **262**, 173-187 (2006).
11. Soreng, R. J. *et al.* A worldwide phylogenetic classification of the Poaceae (Gramineae) III: An update. *J. Syst. Evol.* **60**, 476-521 (2022).
12. Zhang, L. *et al.* Phylotranscriptomics resolves the phylogeny of Pooideae and uncovers factors for their adaptive evolution. *Mol. Biol. Evol.* **39**, msac026 (2022).
13. Glémin, S. *et al.* Pervasive hybridizations in the history of wheat relatives. *Sci. Adv.* **5**, eaav9188 (2019).
14. Li, L.-F. *et al.* Genome sequences of five Sitopsis species of *Aegilops* and the origin of polyploid wheat B subgenome. *Mol. Plant* **15**, 488-503 (2022).
15. Duret, L. & Galtier, N. Biased gene conversion and the evolution of Mammalian genomic landscapes. *Annu. Rev. Genomics Hum. Genet.* **10**, 285-311 (2009).
16. Mori, M. *et al.* Isolation and characterization of a rice dwarf mutant with a defect in brassinosteroid biosynthesis. *Plant Physiol.* **130**, 1152-1161 (2002).
17. Makarevitch, I., Thompson, A., Muehlbauer, G. J. & Springer, N. M. *Brd1* gene in maize encodes a Brassinosteroid C-6 oxidase. *PLoS ONE* **7**, e30798 (2012).
18. Niu, M. *et al.* Rice DWARF AND LOW-TILLERING and the homeodomain protein OSH15 interact to regulate internode elongation via orchestrating brassinosteroid signaling and metabolism. *Plant Cell* **34**, 3754-3772 (2022).
19. Zhang, Z. *et al.* Physiological and transcriptomic analyses of brassinosteroid function in moso bamboo (*Phyllostachys edulis*) seedlings. *Planta* **252**, 27 (2020).
20. Reynoso, M. A. *et al.* Evolutionary flexibility in flooding response circuitry in angiosperms. *Science* **365**, 1291-1295 (2019).
21. Yao, D. *et al.* Transcriptomic profiling of wheat stem during meiosis in response to freezing stress. *Front. Plant Sci.* **13**, (2023).
22. Varoquaux, N. *et al.* Transcriptomic analysis of field-droughted sorghum from seedling to maturity reveals biotic and metabolic responses. *Proc. Natl. Acad. Sci. U. S. A.* **116**, 27124-27132 (2019).
23. Xue, C. *et al.* Tissue-level transcriptomic responses to local and distal chilling reveal potential chilling survival mechanisms in maize. *J. Exp. Bot.* **72**, 7610-7625 (2021).

24. Huang, W. *et al.* A well-supported nuclear phylogeny of Poaceae and implications for the evolution of C4 photosynthesis. *Mol. Plant* **15**, 755-777 (2022).

25. VanBuren, R. *et al.* Single-molecule sequencing of the desiccation-tolerant grass *Oropetium thomaeum*. *Nature* **527**, 508-511 (2015).

26. Zhang, H. *et al.* Transcriptome analysis reveals unique relationships among *Eleusine* species and heritage of *Eleusine coracana*. *G3 (Bethesda)* **9**, 2029-2036 (2019).

27. Tanaka, H. *et al.* Sequencing and comparative analyses of the genomes of zoysiagrasses. *DNA Res.* **23**, 171-180 (2016).

28. VanBuren, R. *et al.* Exceptional subgenome stability and functional divergence in the allotetraploid Ethiopian cereal teff. *Nat. Commun.* **11**, 884 (2020).

29. Garsmeur, O. *et al.* A mosaic monoploid reference sequence for the highly complex genome of sugarcane. *Nat. Commun.* **9**, 2638 (2018).

30. Zhang, J. *et al.* Allele-defined genome of the autopolyploid sugarcane *Saccharum spontaneum* L. *Nat. Genet.* **50**, 1565-1573 (2018).

31. Paterson, A. H. *et al.* The *Sorghum bicolor* genome and the diversification of grasses. *Nature* **457**, 551-556 (2009).

32. Schnable, P. S. *et al.* The B73 maize genome: complexity, diversity, and dynamics. *Science* **326**, 1112-1115 (2009).

33. Varshney, R. K. *et al.* Pearl millet genome sequence provides a resource to improve agronomic traits in arid environments. *Nat. Biotechnol.* **35**, 969-976 (2017).

34. Bennetzen, J. L. *et al.* Reference genome sequence of the model plant *Setaria*. *Nat. Biotechnol.* **30**, 555-561 (2012).

35. Zou, C. S. *et al.* The genome of broomcorn millet. *Nat. Commun.* **10**, 436 (2019).

36. Lovell, J. T. *et al.* The genomic landscape of molecular responses to natural drought stress in *Panicum hallii*. *Nat. Commun.* **9**, 5213 (2018).

37. Studer, A. J. *et al.* The draft genome of the C3 panicoid grass species *Dichanthelium oligosanthes*. *Genome Biol.* **17**, 223 (2016).

38. Washburn, J. D. *et al.* Genome-guided phylo-transcriptomic methods and the nuclear phylogenetic tree of the Paniceae grasses. *Sci. Rep.* **7**, 13528 (2017).

39. International Wheat Genome Sequencing Consortium. Shifting the limits in wheat research and breeding using a fully annotated reference genome. *Science* **361**, eaar7191 (2018).

40. Avni, R. *et al.* Wild emmer genome architecture and diversity elucidate wheat evolution and domestication. *Science* **357**, 93-97 (2017).

41. Maccaferri, M. *et al.* Durum wheat genome highlights past domestication signatures and future improvement targets. *Nat. Genet.* **51**, 885-895 (2019).

42. Olivera, P. D., Kolmer, J. A., Anikster, Y. & Steffenson, B. J. Resistance of sharon goatgrass (*Aegilops sharonensis*) to fungal diseases of wheat. *Plant Dis.* **91**, 942-950 (2007).

43. Luo, M.-C. *et al.* Genome sequence of the progenitor of the wheat D genome *Aegilops tauschii*. *Nature* **551**, 498-502 (2017).

44. Sarah, G. *et al.* A large set of 26 new reference transcriptomes dedicated to comparative population genomics in crops and wild relatives. *Mol. Ecol. Resour.* **17**, 565-580 (2017).

45. Bauer, E. *et al.* Towards a whole-genome sequence for rye (*Secale cereale* L.). *Plant J.* **89**, 853-869 (2017).

46. Wang, H. *et al.* Horizontal gene transfer of *Fhb7* from fungus underlies *Fusarium* head blight resistance in wheat. *Science* **368**, eaba5435 (2020).

47. Mascher, M. *et al.* A chromosome conformation capture ordered sequence of the barley genome. *Nature* **544**, 427-433 (2017).

48. Vogel, J. P. *et al.* Genome sequencing and analysis of the model grass *Brachypodium distachyon*. *Nature* **463**, 763-768 (2010).

49. Zheng, Y. *et al.* Allele-aware chromosome-scale assembly of the allopolyploid genome of hexaploid Ma bamboo (*Dendrocalamus latiflorus* Munro). *J. Integr. Plant Biol.* **64**, 649-670 (2022).

50. Guo, Z.-H. *et al.* Genome sequences provide insights into the reticulate origin and unique traits of woody bamboos. *Mol. Plant* **12**, 1353-1365 (2019).

51. Zhao, H. *et al.* Analysis of 427 genomes reveals moso bamboo population structure and genetic basis of property traits. *Nat. Commun.* **12**, 5466 (2021).

52. Mao, L. *et al.* RiceRelativesGD: a genomic database of rice relatives for rice research. *Database* **2019**, (2019).

53. Ouyang, S. *et al.* The TIGR Rice Genome Annotation Resource: improvements and new features. *Nucleic Acids Res.* **35**, D883-D887 (2006).

54. Shang, L. G. *et al.* A super pan-genomic landscape of rice. *Cell Res.* **32**, 878-896 (2022).

55. Zhang, Y. *et al.* Genome and comparative transcriptomics of African wild rice *Oryza longistaminata* provide insights into molecular mechanism of rhizomatousness and self-incompatibility. *Mol. Plant* **8**, 1683-1686 (2015).

56. Stein, J. C. *et al.* Genomes of 13 domesticated and wild rice relatives highlight genetic conservation, turnover and innovation across the genus *Oryza*. *Nat. Genet.* **50**, 285-296 (2018).

57. Phillips, A. L. *et al.* The first long-read nuclear genome assembly of *Oryza australiensis*, a wild rice from northern Australia. *Sci. Rep.* **12**, 10823 (2022).

58. Shenton, M. *et al.* Evolution and diversity of the wild rice *Oryza officinalis* complex, across continents, genome types, andploidy levels. *Genome Biol. Evol.* **12**, 413-428 (2020).

59. Bansal, J., Gupta, K., Rajkumar, M. S., Garg, R. & Jain, M. Draft genome and transcriptome analyses of halophyte rice *Oryza coarctata* provide resources for salinity and submergence stress response factors. *Physiol. Plant.* **173**, 1309-1322 (2021).

60. Wu, Z. *et al.* De novo genome assembly of *Oryza granulata* reveals rapid genome expansion and adaptive evolution. *Commun. Biol.* **1**, 84 (2018).

61. Yan, N. *et al.* Chromosome-level genome assembly of *Zizania latifolia* provides insights into its seed shattering and phytocassane biosynthesis. *Commun. Biol.* **5**, 36 (2022).

62. Ma, P.-F. *et al.* The *Pharus latifolius* genome bridges the gap of early grass evolution. *Plant Cell* **33**, 846-864 (2021).

63. Seetharam, A. S. *et al.* The *Streptochaeta* genome and the evolution of the grasses. *Front. Plant Sci.* **12**, 710383 (2021).

64. Leebens-Mack, J. H. *et al.* One thousand plant transcriptomes and the phylogenomics of green plants. *Nature* **574**, 679-685 (2019).

65. Xiang, Y. *et al.* Angiosperm-wide analysis of fruit and ovary evolution aided by a new nuclear phylogeny supports association of the same ovary type with both dry and fleshy fruits. *J. Integr. Plant Biol.* **66**, 228-251 (2024).

66. Can, M. *et al.* Genome sequence of *Kobresia littledalei*, the first chromosome-level genome in the family Cyperaceae. *Sci. Data* **7**, 175 (2020).

67. Ming, R. *et al.* The pineapple genome and the evolution of CAM photosynthesis. *Nat. Genet.* **47**, 1435-1442 (2015).

68. Singh, R. *et al.* Oil palm genome sequence reveals divergence of interfertile species in Old and New worlds. *Nature* **500**, 335-339 (2013).

69. D’Hont, A. *et al.* The banana (*Musa acuminata*) genome and the evolution of monocotyledonous plants. *Nature* **488**, 213-217 (2012).

70. Zhang, G.-Q. *et al.* The *Apostasia* genome and the evolution of orchids. *Nature* **549**, 379-383 (2017).

71. Siadjeu, C., Pucker, B., Viehöver, P., Albach, D. C. & Weisshaar, B. High contiguity de novo genome sequence assembly of trifoliate yam (*Dioscorea dumetorum*) using long read sequencing. *Genes* **11**, 274 (2020).

72. Olsen, J. L. *et al.* The genome of the seagrass *Zostera marina* reveals angiosperm adaptation to the sea. *Nature* **530**, 331-335 (2016).

73. Shi, T. *et al.* The slow-evolving *Acorus tatarinowii* genome sheds light on ancestral monocot evolution. *Nat. Plants* **8**, 764-777 (2022).

74. Yuan, Z. *et al.* The pomegranate (*Punica granatum* L.) genome provides insights into fruit quality and ovule developmental biology. *Plant Biotechnol. J.* **16**, 1363-1374 (2018).

75. Guo, L. *et al.* The opium poppy genome and morphinan production. *Science* **362**, 343-347 (2018).

76. Zhang, L. *et al.* The water lily genome and the early evolution of flowering plants. *Nature* **577**, 79-84 (2020).

77. Albert, V. A. *et al.* The amborella genome and the evolution of flowering plants. *Science* **342**, 1241089 (2013).

78. Peng, Z. *et al.* Transcriptome sequencing and analysis of the fast growing shoots of moso bamboo (*Phyllostachys edulis*). *PLoS ONE* **8**, e78944 (2013).

79. Zhao, H. *et al.* Transcriptome and comparative gene expression analysis of *Phyllostachys edulis* in response to high light. *BMC Plant Biol.* **16**, 34 (2016).

80. Wang, T. *et al.* Comprehensive profiling of rhizome-associated alternative splicing and alternative polyadenylation in moso bamboo (*Phyllostachys edulis*). *Plant J.* **91**, 684-699 (2017).

81. Wang, Y. *et al.* Genome-Wide profiling of circular RNAs in the rapidly growing shoots of moso bamboo (*Phyllostachys edulis*). *Plant Cell Physiol.* **60**, 1354-1373 (2019).

82. Zhang, W. *et al.* A high-quality genome sequence of alkaligrass provides insights into halophyte stress tolerance. *Sci. China Life Sci.* **63**, 1269-1282 (2020).

83. Li, W. *et al.* Draft genome of the herbaceous bamboo *Raddia distichophylla*. *G3 (Bethesda)* **11**, jkaa049 (2021).

84. Rohini, G. *et al.* Deep transcriptome sequencing of wild halophyte rice, *Porteresia coarctata*, provides novel insights into the salinity and submergence tolerance factors. *DNA Res.* **21**, 69-84 (2014).

85. Lamesch, P. *et al.* The Arabidopsis Information Resource (TAIR): improved gene annotation and new tools. *Nucleic Acids Res.* **40**, D1202-D1210 (2011).

86. Daccord, N. *et al.* High-quality de novo assembly of the apple genome and methylome dynamics of early fruit development. *Nat. Genet.* **49**, 1099 (2017).

87. Jaillon, O. *et al.* The grapevine genome sequence suggests ancestral hexaploidization in major angiosperm phyla. *Nature* **449**, 463-467 (2007).

88. Tomato Genome Consortium. The tomato genome sequence provides insights into fleshy fruit evolution. *Nature* **485**, 635-641 (2012).

89. Gui, S. *et al.* Improving *Nelumbo nucifera* genome assemblies using high-resolution genetic maps and BioNano genome mapping reveals ancient chromosome rearrangements. *Plant J.* **94**, 721-734 (2018).
