# Supplementary material for: Phylogenomic profiles of whole-genome duplications in Poaceae and landscape of differential duplicate retention and losses among major Poaceae lineages
Source: Nat Commun. 2024 Apr 17;15:3305. doi: 10.1038/s41467-024-47428-9 (PMC11024178; doi:10.1038/s41467-024-47428-9)
Supplement: Supplementary file 3 — Description of Additional Supplementary Files [file 41467_2024_47428_MOESM3_ESM.pdf]

Description of Additional Supplementary Files

Supplementary Data 1 Summary of published datasets used in our study.

Supplementary Data 2 Summary of assemblies.

Supplementary Data 3 Dating of WGDs in Poaceae and other Poales species.

Supplementary Data 4 Summary of K and L genes shown in Fig. 5b.

Supplementary Data 5 Summary of retention and loss events of the rho-derived genes in different subfamilies.

Supplementary Data 6 GO enrichment of genes derived from rho.
