# Supplementary material for: Phylogenomic profiles of whole-genome duplications in Poaceae and landscape of differential duplicate retention and losses among major Poaceae lineages
Source: Nat Commun. 2024 Apr 17;15:3305. doi: 10.1038/s41467-024-47428-9 (PMC11024178; doi:10.1038/s41467-024-47428-9)
Supplement: Supplementary file 10 — Reporting Summary [file 41467_2024_47428_MOESM10_ESM.pdf]

nature portfolio

Corresponding author(s): Hong Ma

Last updated by author(s): Mar 28, 2024

Reporting Summary

Nature Portfolio wishes to improve the reproducibility of the work that we publish. This form provides structure for consistency and transparency in reporting. For further information on Nature Portfolio policies, see our Editorial Policies and the Editorial Policy Checklist.

Statistics

For all statistical analyses, confirm that the following items are present in the figure legend, table legend, main text, or Methods section.

n/a	Confirmed	
☐	☒	The exact sample size (*n*) for each experimental group/condition, given as a discrete number and unit of measurement
☒	☐	A statement on whether measurements were taken from distinct samples or whether the same sample was measured repeatedly
☐	☒	The statistical test(s) used AND whether they are one- or two-sided<br>*Only common tests should be described solely by name; describe more complex techniques in the Methods section.*
☒	☐	A description of all covariates tested
☐	☒	A description of any assumptions or corrections, such as tests of normality and adjustment for multiple comparisons
☐	☒	A full description of the statistical parameters including central tendency (e.g. means) or other basic estimates (e.g. regression coefficient) AND variation (e.g. standard deviation) or associated estimates of uncertainty (e.g. confidence intervals)
☐	☒	For null hypothesis testing, the test statistic (e.g. *F*, *t*, *r*) with confidence intervals, effect sizes, degrees of freedom and *P* value noted<br>*Give P values as exact values whenever suitable.*
☐	☒	For Bayesian analysis, information on the choice of priors and Markov chain Monte Carlo settings
☒	☐	For hierarchical and complex designs, identification of the appropriate level for tests and full reporting of outcomes
☒	☐	Estimates of effect sizes (e.g. Cohen's *d*, Pearson's *r*), indicating how they were calculated

Our web collection on statistics for biologists contains articles on many of the points above.

Software and code

Policy information about availability of computer code

Data collection	Data available in NCBI were retrieved based on the accession numbers listed in Supplementary Data 1. Other published and public available data were downloaded from the web portals of the data sources used in Supplementary Data 1.
Data analysis	The software, tools, and packages used for data analyses are described and their sources are cited in the Methods section. The following software, tools, and packages were used: BLAST v2.10.0, Trinity v2.2.0 and its in-built tool Trimmomatic v0.32, TransDecoder v5.5.0, SOAPdenovo v2.04-r240, Diamond v2.0.4.142, GeneWise v2-4-1, TransMCL v1 and its in-built tool IsoSVM v2004, BUSCO v5.2.2, IQ-TREE v2.1.2, ASTRAL-Pro v1.3.1.0, the baseml and MCMCTree tools in PAML v4.9, deeptime package v1.0.1, ggtree v1.14.6, PhyloMCL v2.0, PASTA v1.8.5, FastTree v2, MAFFT v7.372, MUSCLE v3.8.425, script of PAL2NAL.pl v14, trimAl v1.4.rev22, Tree2GD v1.0.40, WGDgc v1.3, the GenPhyloData in JPrIME v0.3.7, MAPS (https://bitbucket.org/barkerlab/maps/), the hmmemit tool in HMMER package v3.4, the online InterPro program (https://www.ebi.ac.uk/interpro/search/sequence/), clusterProfiler v3.10.1, GOATOOLS v1.3.9, the MCScan (Python version) in JCVI v1.1.15, script of getRBH.pl (https://github.com/Computational-conSequences/SequenceTools/), kallisto v0.46.1, and mad v2.2. Our custom scripts are available at https://github.com/TaikuiZhang/GrassPhylogenomics and https://doi.org/10.24433/CO.1170454.v1. The rationale for each analysis, the input data and the parameters used are described in the Methods section.

For manuscripts utilizing custom algorithms or software that are central to the research but not yet described in published literature, software must be made available to editors and reviewers. We strongly encourage code deposition in a community repository (e.g. GitHub). See the Nature Portfolio guidelines for submitting code & software for further information.

Data

Policy information about availability of data

All manuscripts must include a data availability statement. This statement should provide the following information, where applicable:

- Accession codes, unique identifiers, or web links for publicly available datasets
- A description of any restrictions on data availability
- For clinical datasets or third party data, please ensure that the statement adheres to our policy

This study did not generate any newly sequenced data. The sequenced data used here are published and public availability, including 462 transcriptomes, 4 genome skimming datasets, and 68 genomes. Datasets including the sequence alignments for molecular dating, sequence alignments and gene tree files of orthogroups and their reconciliations for Tree2GD analyses, sequence alignments and gene tree files for MAPS analyses, sequence alignments and gene tree files for bamboo genome analyses, gene tree files for Oryza ASTRAL analyses, and sequence alignments and gene tree files for estimating the retention and loss patterns of the rho-derived duplicates, are available at https://doi.org/10.6084/m9.figshare.25377439.v1. The relevant data for Figures 1-8 can be found in the Source Data file. Source Data for Supplementary Figures are also provided in the Source Data file. The accessions in Supplementary Data 1 are available in NCBI and other public databases. Specific databases used in our analyses include the SILVA database (releases/24-Aug-2020; https://www.arb-silva.de/fileadmin/silva_databases/release_138_1/Exports/SILVA_138.1_SSURef_tax_silva_trunc.fasta.gz), the Monocotyledons specific BUSCO database (liliopsida_odb10; https://busco-data.ezlab.org/v4/data/lineages/liliopsida_odb10.2020-09-10.tar.gz), and the basic-go database (releases/2023-11-15; http://purl.obolibrary.org/obo/go/go-basic.obo).

Research involving human participants, their data, or biological material

Policy information about studies with human participants or human data. See also policy information about sex, gender (identity/presentation), and sexual orientation and race, ethnicity and racism.

Reporting on sex and gender	N/A
Reporting on race, ethnicity, or other socially relevant groupings	N/A
Population characteristics	N/A
Recruitment	N/A
Ethics oversight	N/A

Note that full information on the approval of the study protocol must also be provided in the manuscript.

Field-specific reporting

Please select the one below that is the best fit for your research. If you are not sure, read the appropriate sections before making your selection.

☐ Life sciences ☐ Behavioural & social sciences ☒ Ecological, evolutionary & environmental sciences

For a reference copy of the document with all sections, see nature.com/documents/nr-reporting-summary-flat.pdf

Ecological, evolutionary & environmental sciences study design

All studies must disclose on these points even when the disclosure is negative.

Study description	We performed a range of phylogenomic and comparative genomic analyses to detect WGDs in grasses, to investigate relationships between GD clusters at successive nodes and WGDs, to detect evidence for potential hybridizations, and to explore lineage-specific retention of rho-derived gene pairs and those from other WGDs in grasses.
Research sample	Our study utilized published genome and transcriptome datasets for WGD analyses. The data sources are listed in Supplementary Data 1. All species except crops are wild species.
Sampling strategy	Based on the BUSCO estimation of the published 342 transcriptomes and 7 genome skimming datasets that sequenced in our lab, we selected 319 datasets (315 transcriptomes and 4 genome skimming datasets) for our analyses here. These datasets were integrated with 46 genomes and 7 transcriptomes from public database to represent 363 species, covering 45 tribes and all 12 subfamilies of Poaceae. For WGD analyses, published genomes instead of the transcriptomes sequenced in our lab for nine species (Zizania latifolia, Oryza australiensis, Oryza officinalis, Oryza punctata, Oryza rufipogon, Oryza meyeriana ssp granulata, Dendrocalamus latiflorus, Pharus latifolius, and Streptochaeta angustifolia) were used to take advantage of the higher quality and the available information of gene order in chromosomes. Besides the 363 species, we also included 5 other grass genomes and 27 datasets (15 transcriptomes and 12 genomes) from 27 non-grass species. These 27 non-grass species were used as outgroups. Five other genomes were used as other representatives of eudicots. In addition, 125 available transcriptomes were retrieved for the comparison of the genes in Triticum aestivum, Sorghum bicolor, Zea mays, Phyllostachys edulis, and Oryza coarctata.

Data collection	Data were downloaded from public repositories (NCBI and a range of species- and lineage-specific repositories) by the first author.
Timing and spatial scale	Data were retrieved during the period from August 1, 2020 to April 10, 2023. To obtain syntenic genes as strong evidence for WGDs, we replaced our transcriptome sequenced species with sequenced genome when available. No attempt was made to control the spatial scale.
Data exclusions	Our analysis replaced transcriptome sequenced species with available sequenced genome, for the reasons described above.
Reproducibility	1000 ultrafast bootstrap replicates were performed by IQ-tree for estimating all nodes in gene tree reconstruction in most parts of our analyses.
Randomization	As a phylogenomic analysis, the data were not randomized. This is standard community practice in phylogenomics, motivated by the evidence that the best available phylogenetic estimates usually are obtained from representative and broadly-sampled datasets.
Blinding	Blinding was not relevant to our phylogenomic analyses, because the results from sequenced datasets are quantitative and did not require subjective judgment or interpretation.

Did the study involve field work? ☐ Yes ☒ No

Reporting for specific materials, systems and methods

We require information from authors about some types of materials, experimental systems and methods used in many studies. Here, indicate whether each material, system or method listed is relevant to your study. If you are not sure if a list item applies to your research, read the appropriate section before selecting a response.

Materials & experimental systems

n/a	Involved in the study
☒	☐ Antibodies
☒	☐ Eukaryotic cell lines
☒	☐ Palaeontology and archaeology
☒	☐ Animals and other organisms
☒	☐ Clinical data
☒	☐ Dual use research of concern
☒	☐ Plants

Methods

n/a	Involved in the study
☒	☐ ChIP-seq
☒	☐ Flow cytometry
☒	☐ MRI-based neuroimaging
